# Supplementary material for: Vitamin D prescribing in children in UK primary care practices: a population-based cohort study
Source: BMJ Open. 2019 Dec 3;9(12):e031870. doi: 10.1136/bmjopen-2019-031870 (PMC6937102; doi:10.1136/bmjopen-2019-031870)
Supplement: Supplementary data [file bmjopen-2019-031870supp001.pdf]

Supplementary Table S1

Summary of UK guidance for the management of vitamin D deficiency in children

Supplementary Figure S1

Flowchart of study cohort selection

Supplementary Table S2

Drug code related to single-ingredient vitamin D products

Supplementary Table S3

Read codes related to vitamin D deficiency and 25-hydroxyvitamin D tests

Supplementary Table S4

No. of children with a record of diagnosis Read codes suggestive of vitamin D deficiency in the 90 days prior to or on the date of the incident prescription, stratified by pre-supplementation 25-hydroxyvitamin D concentrations

Supplementary Table S5

Multivariable Poisson regression models of incidence of vitamin D supplementation prescribing in children stratified by sex (Complete-cases only).

Supplementary Figure S2

Time trends in vitamin D supplementation prescribing in children by product types

Supplementary Table S6

Doses of vitamin D supplementation prescribed stratified by age group

Supplementary Table S7

Vitamin D supplementation dosing frequencies observed in the study cohort

Wan M, *et al. BMJ Open* 2019; 9:e031870. doi: 10.1136/bmjopen-2019-031870

Supplementary Table S1

Summary of UK guidance for the management of vitamin D deficiency in children

Guideline	Definition of vitamin D deficiency	Recommendations on supplementation for primary prevention	Recommendations on supplement for treatment of vitamin D deficiency
SACN (2007)	<25 nmol/L	0-6 months: 340 IU daily;<br>7 months to 3 years: 280 IU daily	No recommendations
NICE (2008)	<25 nmol/L	6 months to 4 years: 300 IU daily	No recommendations
DHSC (2012)	Not explicitly defined	6 months to 5 years: 280 - 340 IU daily<br>Those infants who are fed infant formula will not need vitamin drops until they are receiving less than 500ml of infant formula a day. Breastfed infants from one month of age if their mother has not taken vitamin D supplements throughout pregnancy.	No recommendations
BPABG (2012)	<25 nmol/L<br><br>25-50 nmol/L defined as insufficiency	6 months to 5 years: as per DHSC (2012) recommendations<br>Exclusively breastfed infants receive vitamin D supplements from soon after birth.	No recommendations
RCPCH (2012)	Not explicitly defined	As per NICE (2008) and DHSC (2012) recommendations	No recommendations
RCPCH (2013)	<25 nmol/L<br><br>25-50 nmol/L defined as insufficiency	Newborn up to 1 month: 300 - 400 IU daily<br>1 month - 18 years: 400 - 1,000 IU daily	Up to 6 months: 1,000 - 3,000 IU daily*<br>6 months - 12 years: 6,000 IU daily*<br>12 -18 years: 10,000 IU daily*<br>*For 4 - 8 weeks
NICE (2014)	<25 nmol/L	0 - 6 months: 340 IU daily<br>Older infants and children up to the age of 5: 280 IU daily	No recommendations
NOS (2015)	<25 nmol/L<br><br>25-50 nmol/L defined as insufficiency	As per DHSC (2012) recommendations	1- 6 months: 3,000 IU daily*<br>6 months - 12 years: 6,000 IU daily*<br>12 -18 years: 10,000 IU daily*<br>*For 8 – 12 weeks
SACN (2016)	<25 nmol/L	0 up to 1 year (including exclusively and partially breast-fed infants, from birth): 340 – 400 IU daily<br>1 year or older: 400 IU daily	No recommendations
NICE CKS (2016)	<25 nmol/L	As per SACN (2016) recommendations	1- 6 months: 3,000 IU daily*<br>6 months - 12 years: 6,000 IU daily*<br>12 -18 years: 10,000 IU daily*<br>*For 8 – 12 weeks

Key:
- SACN (2007): Scientific Advisory Committee on Nutrition - Update on Vitamin D[31]
- NICE (2008): The National Institute for Health and Care Excellence - Improving the nutrition of pregnant and breastfeeding mothers and children in low-income households[32]
- DHSC (2012): Department of Health and Social Care - Vitamin D - advice on supplements for at risk groups[33]
- BPABG (2012): British Paediatric and Adolescent Bone Group's position statement on vitamin D deficiency[34]
- RCPCH (2012): Royal College of Paediatrics and Child Health - Vitamin D: Position statement[35]
- RCPCH (2013): Royal College of Paediatrics and Child Health - Guide for Vitamin D in Childhood[36]
- NICE (2014): The National Institute for Health and Care Excellence - Vitamin D: increasing supplement use in at-risk groups[37]
- NOS (2015): National Osteoporosis Society - Vitamin D and Bone Health[38]
- SACN (2016): Scientific Advisory Committee on Nutrition - vitamin D and health report[39]
- NICE CKS (2016): The National Institute for Health and Care Excellence - Clinical Knowledge Summaries - Vitamin D deficiency in children[40]

Wan M, *et al. BMJ Open* 2019; 9:e031870. doi: 10.1136/bmjopen-2019-031870

Supplementary Figure S1

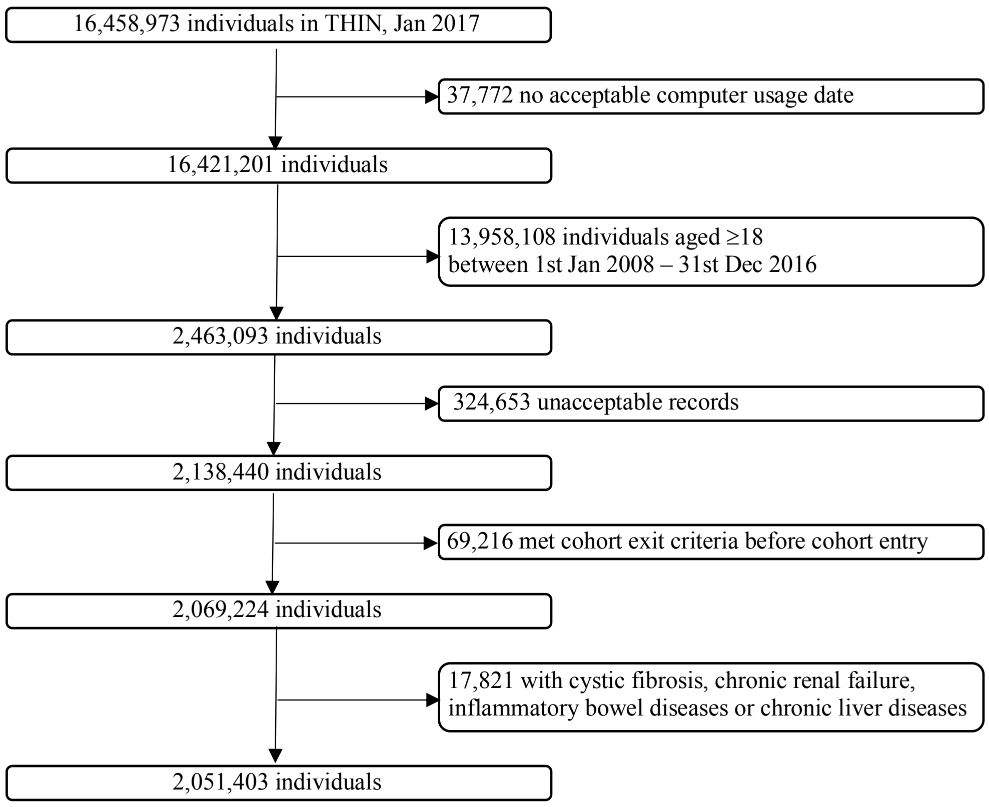

Figure S1: Flowchart of study cohort selection. The diagram displays the numbers of patients excluded from the THIN cohort and reasons for exclusion, leading to selection of the study cohort.

Wan M, *et al. BMJ Open* 2019; 9:e031870. doi: 10.1136/bmjopen-2019-031870

Supplementary Table S2

Drug code related to single-ingredient vitamin D products

2a. Drug code related to single-ingredient vitamin D products

Drug code	Description
29742978	colecalciferol 800unit capsules
30212978	colecalciferol 2,500units/drop oral drops sugar free
30582978	colecalciferol 40,000unit tablets
30643978	colecalciferol 800unit capsules
32236978	colecalciferol 4,000unit tablets
32237978	colecalciferol 4,000unit tablets
32265978	colecalciferol 4,000unit capsules
32267978	colecalciferol 1,000unit tablets
32317978	colecalciferol 50,000unit capsules
34135978	colecalciferol 3,000unit tablets
34138978	colecalciferol 800unit capsules
34144978	colecalciferol 1,000unit capsules
38846978	colecalciferol 20,000unit tablets
39354978	colecalciferol 50,000units/1ml oral solution unit dose ampoules sugar free
39355978	colecalciferol 50,000units/1ml oral solution unit dose ampoules sugar free
47464978	colecalciferol 2,740units/ml oral drops sugar free
47465978	colecalciferol 2,740units/ml oral drops sugar free
48011978	colecalciferol 15,000units/5ml oral solution
51017978	colecalciferol 15,000units/5ml oral solution
51018978	colecalciferol 15,000units/5ml oral solution
51473978	colecalciferol 200units/drop oral drops sugar free
51484978	colecalciferol 1,000unit capsules
52073979	colecalciferol 40,000unit capsules
52074979	colecalciferol 40,000unit capsules
52154979	colecalciferol 20,000unit capsules
53089979	colecalciferol 15,000units/5ml oral solution
53090979	colecalciferol 15,000units/5ml oral solution
53091979	colecalciferol 15,000units/5ml oral solution
53092979	colecalciferol 15,000units/5ml oral solution
53321979	ergocalciferol 125microgram tablets
53322979	ergocalciferol 125microgram tablets
53352979	colecalciferol 20,000unit tablets
53353979	colecalciferol 5,000unit tablets
53355979	colecalciferol 400unit tablets
53356979	colecalciferol 30,000unit capsules
53357979	colecalciferol 5,000unit capsules
53358979	colecalciferol 3,000unit capsules
53359979	ergocalciferol 12.5microgram tablets
53362979	colecalciferol 400unit capsules
53365979	colecalciferol 1,000unit capsules
53366979	colecalciferol 600unit capsules
53367979	colecalciferol 2,200unit capsules
53368979	colecalciferol 10,000unit capsules
53369979	colecalciferol 20,000unit capsules
53370979	colecalciferol 20,000unit capsules
53371979	colecalciferol 50,000unit capsules
53377978	colecalciferol 2,400units/ml oral drops sugar free
53378978	colecalciferol 2,400units/ml oral drops sugar free
53461978	ergocalciferol 12.5microgram tablets
53464978	colecalciferol 20,000unit capsules
53641979	ergocalciferol 250microgram tablets
53650979	ergocalciferol 1.25mg capsules
54637979	colecalciferol 500unit capsules
54638979	colecalciferol 500unit capsules
54716979	colecalciferol 1,000unit capsules
54790979	ergocalciferol 1.25mg capsules
54889979	colecalciferol 3,000units/ml oral solution
54890979	colecalciferol 2,000units/ml oral solution

Wan M, *et al. BMJ Open* 2019; 9:e031870. doi: 10.1136/bmjopen-2019-031870

2a. Drug code related to single-ingredient vitamin D products - continued

Drug code	Description
54891979	colecalciferol 2,000units/ml oral solution
54916979	colecalciferol 800unit tablets
54917979	colecalciferol 800unit tablets
55053979	colecalciferol 3,000unit tablets
55054979	colecalciferol 20,000unit tablets
55055979	colecalciferol 2,200unit tablets
55056979	colecalciferol 10,000unit tablets
55060979	colecalciferol 5,000unit tablets
55061979	ergocalciferol 3,000units/ml oral solution
55062979	ergocalciferol 3,000units/ml oral solution sugar free
55064979	colecalciferol 3,000units/ml oral solution sugar free
55065979	colecalciferol 20,000units/ml oral solution sugar free
55066979	colecalciferol 30,000unit capsules
55067979	colecalciferol 30,000unit capsules
55068979	colecalciferol 5,000unit capsules
55070979	colecalciferol 400unit capsules
55072979	colecalciferol 1,000unit capsules
55074979	colecalciferol 2,200unit capsules
55076979	colecalciferol 10,000unit capsules
55077979	colecalciferol 20,000unit capsules
55078979	colecalciferol 50,000unit capsules
55079979	colecalciferol 50,000unit capsules
55194978	colecalciferol 10,000units/ml oral solution sugar free
55195978	colecalciferol 10,000units/ml oral drops sugar free
55196978	colecalciferol 10,000units/ml oral drops sugar free
55197978	colecalciferol 10,000units/ml oral drops sugar free
55257978	colecalciferol 20,000unit capsules
55558979	ergocalciferol 50,000unit capsules
56123979	ergocalciferol 1.25mg capsules
56242979	colecalciferol 3,000unit tablets
56244979	colecalciferol 20,000unit tablets
56246979	colecalciferol 2,200unit tablets
56248979	colecalciferol 10,000unit tablets
56310979	colecalciferol 400unit tablets
56313979	colecalciferol 1,000unit tablets
56949978	colecalciferol 25,000unit tablets
56950978	colecalciferol 25,000unit tablets
57024978	colecalciferol 1,000unit tablets
57965979	colecalciferol 20,000unit capsules
58139979	colecalciferol 5,000unit tablets
58209979	ergocalciferol 3,000units/ml oral solution
58346979	colecalciferol 800unit capsules
58349979	colecalciferol 5,000unit capsules
58353979	colecalciferol 1,000unit capsules
58355979	colecalciferol 20,000unit capsules
58356979	colecalciferol 20,000unit capsules
58768979	colecalciferol 2,000units/ml oral solution sugar free
58769979	colecalciferol 2,000units/ml oral solution sugar free
58782979	colecalciferol 3,000units/ml oral solution sugar free
59522978	colecalciferol 2,000unit tablets
59525978	colecalciferol 40,000unit capsules
59527978	colecalciferol 5,000unit tablets
59528978	colecalciferol 20,000unit capsules
59769979	colecalciferol 800units/ml oral drops sugar free
59770979	colecalciferol 800units/ml oral drops sugar free
59771979	colecalciferol 400units/dose oral spray sugar free
59772979	colecalciferol 400units/dose oral spray sugar free
59843979	colecalciferol 20,000unit capsules
59981979	ergocalciferol 15,000units/5ml oral solution
60056979	ergocalciferol 300,000units/1ml solution for injection ampoules

2a. Drug code related to single-ingredient vitamin D products - continued

Drug code	Description
60224979	colecalciferol 300,000units/1ml solution for injection ampoules
60225979	ergocalciferol 300,000units/1ml solution for injection ampoules
60373979	fultium-d3 800unit capsules
60374979	colecalciferol 800unit capsules
60566979	vitamin d2 3,000units/ml oral solution sugar free
60568979	ergocalciferol 20,000units/ml oral solution sugar free
60572979	colecalciferol 20,000units/ml oral solution sugar free
60594979	colecalciferol 1,000unit tablets
61563979	colecalciferol 30,000units/5ml oral solution
61592979	colecalciferol 2,000units/ml oral drops sugar free
61593979	colecalciferol 2,000units/ml oral drops sugar free
61910979	colecalciferol 400unit tablets
61911979	colecalciferol 400unit tablets
62001979	colecalciferol 1,000units/ml oral solution
62002979	colecalciferol 1,000units/ml oral solution
62087979	colecalciferol 10,000units/ml oral solution
62088979	colecalciferol 10,000units/ml oral solution
62116979	colecalciferol 5,000unit capsules
62118979	colecalciferol 3,000unit capsules
62152979	colecalciferol 6,000units/5ml oral suspension
62324979	colecalciferol 20,000units/ml oral drops
62356979	ergocalciferol 12.5microgram tablets
62357979	ergocalciferol 12.5microgram tablets
62536979	colecalciferol 400unit capsules
62622979	colecalciferol 1,000unit tablets
62626979	colecalciferol 1,000unit capsules
62643979	colecalciferol 600unit capsules
62645979	colecalciferol 2,200unit capsules
62986979	colecalciferol 20,000unit capsules
63201979	colecalciferol 2,000unit capsules
64131979	colecalciferol 4,000units/5ml oral suspension
68364979	ergocalciferol 400units/5ml oral solution
68382979	ergocalciferol 20,000units/5ml oral suspension
68384979	ergocalciferol 20,000units/5ml oral solution
68392979	ergocalciferol 120units/5ml oral solution
68396979	ergocalciferol 100,000units/5ml oral solution
68780978	colecalciferol 20,000unit capsules
68781978	colecalciferol 20,000unit capsules
69915979	colecalciferol 400units/5ml oral suspension
69917979	colecalciferol 20,000units/5ml oral suspension
69920979	colecalciferol 1,000units/5ml oral suspension
70249978	ergocalciferol 1.25mg capsules
73011978	colecalciferol 25,000units/1ml oral solution unit dose ampoules sugar free
73012978	colecalciferol 25,000units/1ml oral solution unit dose ampoules sugar free
73102978	colecalciferol 3,200unit capsules
73128978	colecalciferol 10,000unit capsules
73129978	colecalciferol 20,000unit capsules
73301978	colecalciferol 20,000units/ml oral solution
73391978	colecalciferol 3,200unit capsules
73392978	colecalciferol 3,200unit capsules
77093978	colecalciferol 15,000units/5ml oral solution
78387978	colecalciferol 500unit orodispersible tablets sugar free
78388978	colecalciferol 500unit orodispersible tablets sugar free
78389978	colecalciferol 2,000unit orodispersible tablets sugar free
78390978	colecalciferol 2,000unit orodispersible tablets sugar free
78426978	colecalciferol 2,200unit tablets
78428978	colecalciferol 5,000unit tablets
78441978	colecalciferol 1,000unit capsules
78719978	colecalciferol 2,000unit tablets
78720978	colecalciferol 2,000unit tablets

Wan M, *et al. BMJ Open* 2019; 9:e031870. doi: 10.1136/bmjopen-2019-031870

2a. Drug code related to single-ingredient vitamin D products - continued

Drug code	Description
79321978	colecalciferol 200units/drop oral drops sugar free
79322978	colecalciferol 200units/drop oral drops sugar free
79338978	ergocalciferol 6,000units/5ml oral solution
79340978	ergocalciferol 10,000units/5ml oral solution
79935978	ergocalciferol 600,000units/1.5ml solution for injection ampoules
79960978	colecalciferol 280unit chewable tablets
79961978	colecalciferol 280unit chewable tablets
79993978	colecalciferol 20,000unit tablets
80016978	colecalciferol 400unit capsules
80018978	colecalciferol 1,000unit tablets
80019978	colecalciferol 1,000unit capsules
80022978	colecalciferol 20,000unit capsules
80147979	ergocalciferol 800units/5ml oral suspension
80151979	ergocalciferol 6,000units/5ml oral suspension
80153979	ergocalciferol 50,000units/5ml oral suspension
80157979	ergocalciferol 30,000units/5ml oral suspension
80159979	ergocalciferol 3,000units/5ml oral suspension
80169979	ergocalciferol 10,000units/5ml oral suspension
80416979	colecalciferol 15,000units/5ml oral suspension
80883998	colecalciferol 3,000units/ml oral solution sugar free
80885998	colecalciferol 3,000units/ml oral solution sugar free
80886998	colecalciferol 20,000units/ml oral solution sugar free
80887998	colecalciferol 20,000units/ml oral solution sugar free
80914998	colecalciferol 2,000units/ml oral drops sugar free
80915998	colecalciferol 2,000units/ml oral drops sugar free
80947998	ergocalciferol 20,000units/ml oral solution sugar free
80952998	colecalciferol 800unit capsules
80958998	colecalciferol 20,000unit capsules
81004998	colecalciferol 30,000unit capsules
81007998	colecalciferol 30,000unit capsules
81009998	colecalciferol 10,000unit capsules
81010998	colecalciferol 10,000unit capsules
81011998	colecalciferol 2,500unit capsules
81012998	colecalciferol 2,500unit capsules
81013998	colecalciferol 1,000unit capsules
81014998	colecalciferol 1,000unit capsules
81015998	colecalciferol 400unit capsules
81074998	colecalciferol 5,000unit capsules
81138998	colecalciferol 500 units tablets
81252998	colecalciferol 1,000unit tablets
81253998	colecalciferol 1,000unit tablets
81328998	colecalciferol 800unit capsules
81329998	colecalciferol 800unit capsules
81335998	colecalciferol 20,000unit capsules
81464998	colecalciferol 400unit capsules
81568998	colecalciferol 10000units/ml oral solution
81612998	colecalciferol 1,000unit tablets
81624998	colecalciferol 10,000units/5ml oral suspension
81631998	colecalciferol 2,200unit capsules
81710998	colecalciferol 50,000unit capsules
82283998	colecalciferol 15,000units/5ml oral solution
82513998	colecalciferol 20,000unit capsules
82514998	ergocalciferol 1.25mg capsules
82946978	colecalciferol 20,000unit tablets
82956978	colecalciferol 20,000unit capsules
83392978	colecalciferol 50,000unit tablets
83393978	colecalciferol 50,000unit tablets
83468978	colecalciferol 20,000unit tablets
83469978	colecalciferol 10,000unit tablets
83480978	colecalciferol 800unit capsules

Wan M, *et al. BMJ Open* 2019; 9:e031870. doi: 10.1136/bmjopen-2019-031870

2a. Drug code related to single-ingredient vitamin D products - continued

Drug code	Description
83743978	ergocalciferol 1,000units/5ml oral solution
83744978	colecalciferol 5,000units/5ml oral solution
83746978	colecalciferol 5,000units/5ml oral solution
83747978	colecalciferol 10,000units/5ml oral solution
83748978	colecalciferol 10,000units/5ml oral solution
83750978	colecalciferol 10,000units/5ml oral solution
84072978	colecalciferol 5,000unit tablets
84414978	ergocalciferol 1,500units/ml oral solution sugar free
84586978	colecalciferol 20,000unit tablets
84592978	colecalciferol 400unit tablets
84600978	colecalciferol 1,000unit tablets
84680998	colecalciferol oral liquid
85005998	ergocalciferol oral liquid
85220998	ergocalciferol 600,000units/2ml solution for injection ampoules
85224998	ergocalciferol 300,000units/1ml solution for injection ampoules
85329978	colecalciferol 1,000unit tablets
86614979	ergocalciferol 300,000units/1ml solution for injection ampoules
86615979	ergocalciferol 300,000units/1ml solution for injection ampoules
93163990	ergocalciferol 600,000units/2ml solution for injection ampoules
93164990	ergocalciferol 300,000units/1ml solution for injection ampoules
93384992	calciferol 10 mcg tab
93390992	calciferol 1,000 iu i/u tab
93401992	calciferol 5000 i/u tab
94809990	ergocalciferol 1.25mg tablets
94810990	ergocalciferol 250microgram tablets
95606992	ergocalciferol 600ku/1.5ml solution
95872992	calciferol 3,000 iu tab
95873992	calciferol 30,000 iu/ml inj
95875992	calciferol soln 400u/5ml in arachis oil sol
95877992	calciferol 40,000 iu/ml inj
95878992	calciferol 400 iu/ml sol
96131992	calciferol 400 iu/ml i/u syr
96132992	calciferol .25 mg cap
96133992	calciferol 1 mg cap
96134992	calciferol 1,000 iu i/u cap
96599992	calciferol 75 mcg inj
96600992	ergocalciferol 250microgram tablets
96601992	calciferol 20,000 iu cap
96788998	ergocalciferol 400000units/ml oral solution
97023992	calciferol 100,000 iu/ml inj
97024992	calciferol 600,000 iu/ml inj
97032992	calciferol 300 iu/ml i/u syr
97033992	calciferol drops liq
97034992	calciferol 400 i/u inj
97035992	calciferol 9,000 iu/ml liq
97036992	calciferol 1,000 iu/5ml sol
97037992	calciferol 300 mg tab
97038992	calciferol 15 mcg tab
97238989	ergocalciferol 1.25mg tablets
97238990	ergocalciferol 250microgram tablets
97767998	calciferol bp 3000units/ml solution
98374992	vitamin d2 tab
98421997	ergocalciferol 1.25mg tablets
98421998	ergocalciferol 250microgram tablets
98422997	ergocalciferol 1,000units/5ml oral suspension
98422998	ergocalciferol 3,000units/ml oral solution sugar free
98925998	ergocalciferol 600,000units/2ml solution for injection ampoules
99762990	ergocalciferol 250microgram tablets
99763988	ergocalciferol 250microgram tablets
99763990	ergocalciferol 300,000units/1ml solution for injection ampoules

2a. Drug code related to combination vitamin D products

Drug code	Description
34026978	colecalciferol 1,000unit / calcium carbonate 2.5g chewable tablets
34027978	colecalciferol 1,000unit / calcium carbonate 2.5g chewable tablets
52015979	colecalciferol 800unit / calcium carbonate 1.25g chewable tablets
52016979	colecalciferol 800unit / calcium carbonate 1.25g tablets
56947978	colecalciferol 800unit / calcium carbonate 2.5g chewable tablets
56948978	colecalciferol 800unit / calcium carbonate 2.5g chewable tablets
58270979	generic caldrink d3 liquid
60543979	colecalciferol 100unit / calcium carbonate 400mg chewable tablets
60544979	colecalciferol 100unit / calcium carbonate 400mg chewable tablets
61594979	colecalciferol 150unit / calcium 250mg tablets
61595979	colecalciferol 150unit / calcium 250mg tablets
64223979	calcium carbonate 400mg / ergocalciferol 2.5microgram tablets
64224979	calcium carbonate 400mg / ergocalciferol 2.5microgram tablets
64830979	calcium and ergocalciferol tablets
64831979	colecalciferol 100unit / calcium carbonate 400mg tablets
68756978	colecalciferol 880unit / calcium carbonate 2.5g chewable tablets
68757978	colecalciferol 880unit / calcium carbonate 2.5g chewable tablets
73294978	generic healthy start children's vitamin drops
73598978	colecalciferol 100unit / calcium carbonate 400mg tablets
80849998	calcium carbonate 400mg with colecalciferol 100 units chewable tablets
81191998	colecalciferol 400unit / calcium carbonate 1.5g tablets
81268998	colecalciferol 200unit / calcium carbonate 750mg tablets
81269998	colecalciferol 200unit / calcium carbonate 750mg tablets
81655998	colecalciferol 800unit / calcium carbonate 1.25g chewable tablets
81656998	colecalciferol 800unit / calcium carbonate 1.25g chewable tablets
82214998	colecalciferol 400unit / calcium carbonate 1.25g tablets
82215998	colecalciferol 400unit / calcium carbonate 1.25g chewable tablets
82229978	generic littlevit multivitamin drops
82344998	colecalciferol 400unit / calcium carbonate 1.5g tablets
82429979	colecalciferol 400unit / calcium carbonate 1.25g chewable tablets
82483998	colecalciferol 400unit / calcium carbonate 1.5g chewable tablets
82546978	colecalciferol 400unit / calcium carbonate 1.5g chewable tablets
82752998	generic sandocal+d 1200 effervescent tablets
82753998	calcium lactate gluconate with calcium carbonate and colecalciferol 1200mg calcium with 800iu effervescent tablets
82966998	generic sandocal+d 600 effervescent tablets
82967998	calcium lactate gluconate with calcium carbonate and colecalciferol 600mg calcium with 400iu effervescent tablets
83420998	colecalciferol 400unit / calcium carbonate 1.25g chewable tablets
83638978	colecalciferol 1,000unit / folic acid 400microgram capsules
83639978	colecalciferol 1,000unit / folic acid 400microgram capsules
84212998	risedronate sodium 35mg with calcium carbonate 2500mg & colecalciferol 22micrograms tablets and granules
84286998	colecalciferol 400unit / calcium carbonate 1.5g effervescent tablets
84287998	colecalciferol 400unit / calcium carbonate 1.5g chewable tablets
84292998	colecalciferol 400unit / calcium carbonate 1.5g chewable tablets
84362998	vitamins c & d3 with folic acid tablets
84363998	vitamins c & d3 with folic acid tablets
86598998	alendronic acid 70mg / colecalciferol 70microgram tablets
86599998	alendronic acid 70mg / colecalciferol 70microgram tablets
88002998	dolomite with vitamin d tablets
88144997	calcium carbonate 600mg with vitamin d % micrograms with minerals chewable tablets
88144998	calcium carbonate 600mg with vitamin d and minerals 5 micrograms tablets
88225998	calcium carbonate 600mg with vitamin d and 5 micrograms tablets
88233979	generic abidec multivitamin drops
89183998	colecalciferol 400unit / calcium carbonate 1.25g chewable tablets
89439998	colecalciferol 440unit / calcium carbonate 1.25g effervescent granules sachets
89637998	colecalciferol 400unit / calcium carbonate 1.5g chewable tablets
89647998	colecalciferol 400unit / calcium carbonate 1.5g chewable tablets
89828997	multivitamin and mineral chewable tablets

Wan M, *et al. BMJ Open* 2019; 9:e031870. doi: 10.1136/bmjopen-2019-031870

2a. Drug code related to combination vitamin D products - continued

Drug code	Description
89828998	calcium carbonate 600mg with vitamin d and minerals 5 micrograms tablets
90498998	nicotinic acid with colecalciferol and calcium gluconate tablet
91053998	colecalciferol 800unit / calcium phosphate 3.1g oral powder sachets
91526998	calcium carbonate 600mg with vitamin d and 5 micrograms tablets
91987979	colecalciferol 400unit / calcium carbonate 1.5g chewable tablets
91989979	colecalciferol 400unit / calcium carbonate 1.25g chewable tablets
91990979	colecalciferol 400unit / calcium carbonate 1.25g chewable tablets
91991979	colecalciferol 400unit / calcium carbonate 1.25g chewable tablets
91993979	colecalciferol 400unit / calcium carbonate 1.25g chewable tablets
91994979	colecalciferol 400unit / calcium carbonate 1.25g chewable tablets
91995979	colecalciferol 200unit / calcium carbonate 1.25g chewable tablets
91996979	colecalciferol 400unit / calcium carbonate 1.25g chewable tablets
91997979	colecalciferol 400unit / calcium carbonate 1.25g chewable tablets
91998979	colecalciferol 200unit / calcium carbonate 1.25g chewable tablets
92000979	colecalciferol 200unit / calcium carbonate 1.25g chewable tablets
92001979	colecalciferol 200unit / calcium carbonate 1.25g chewable tablets
92004979	calcium and ergocalciferol tablets
93127992	calcium 500mgs & vitamin d 400iu tab
93276998	generic abidec multivitamin drops
94021990	calcium and ergocalciferol tablets
94089992	calcium + vitamin d 300 mg tab
94756992	calcium & vitamin d 6000 i/u tab
96015992	vit.a /vit.b1 /vit.b2 /vit.c /vit.d2 /ni pow
96020992	calcium and ergocalciferol tablets
96737998	calciferol with calcium tablets
96789998	calcium and ergocalciferol tablets
96897998	calcium and ergocalciferol tablets
96920998	colecalciferol 800unit / calcium phosphate 3.1g oral powder sachets
96959996	colecalciferol 440unit / calcium carbonate 1.25g effervescent granules sachets
96959997	colecalciferol 400unit / calcium carbonate 1.25g chewable tablets
96959998	colecalciferol 200unit / calcium carbonate 1.25g chewable tablets
97031992	calciferol/calcium gluconate tab
97064992	calcium 500mgs & vitamin d 200iu i/u tab
97138998	calcium and ergocalciferol tablets
97248990	calcium and ergocalciferol tablets
97780990	calcium and ergocalciferol tablets
97815990	calcium and ergocalciferol tablets
97935998	generic abidec multivitamin drops
98247992	vit.a /vit.b1 /vit.b2 /vit.c /vit.d2 /ni eli
98249990	calcium and ergocalciferol tablets
98581990	calcium and ergocalciferol tablets
98700997	colecalciferol 200unit / calcium carbonate 1.25g chewable tablets
98700998	colecalciferol 200unit / calcium carbonate 1.25g chewable tablets
99018990	calcium and ergocalciferol tablets
99733992	ascorbic acid/vitamin a/vitamin d 2 25 mg tab

Wan M, *et al*. *BMJ Open* 2019; 9:e031870. doi: 10.1136/bmjopen-2019-031870

Supplementary Table S3

Read codes related to vitamin D deficiency and 25-hydroxyvitamin D tests

Read code	Description
C28..00	vitamin d deficiency
C28..11	osteomalacia
C28..12	rickets
C280.00	active rickets
C282.00	osteomalacia unspecified
C28z.00	avitaminosis d nos
C2B..00	vitamin d insufficiency
J690.11	coeliac rickets
K080300	renal rickets
44LA.00	serum vitamin d
44LM.00	plasma 25-hydroxyvitamin d3 level
44LP.00	serum 25-hydroxy vitamin d3 level
44Lg.00	serum vitamin d2 level
44Ln.00	serum 25-hydroxyvitamin d2 level
4QB4.00	vitamin d level
4QB4000	vitamin d2 level
4QB4100	vitamin d3 level
4QB4200	25-hydroxyvitamin d2 level
4QB4300	25-hydroxyvitamin d3 level
4QB4400	combined total vitamin d2 and d3 level
4QB4500	25-hydroxyvitamin d level
4QB4600	total 25-hydroxyvitamin d level
4QB4700	serum total 25-hydroxy vitamin d level
5788	radiobioassay - vitamin d

Wan M, *et al. BMJ Open* 2019; 9:e031870. doi: 10.1136/bmjopen-2019-031870

Supplementary Table S4

No. of children with a record of diagnosis Read codes suggestive of vitamin D deficiency in the 90 days prior to or on the date of the incident prescription, stratified by pre-supplementation 25-hydroxyvitamin D concentrations

	25-hydroxyvitamin D concentrations at initiation of supplementation (nmol/L)							
	**<10** (n=3,738)	**10 - 20** (n=3,898)	**>20 - 30** (n=441)	**>30 - 50** (n=31)	**>50** (n=9)	**No test** (n=3,986)	**Undetermined** (n=174)	**Total** (n=12,277)
**No. of children with symptoms, n**								
Musculoskeletal and non-specific pain	750	769	67	4	0	340	23	1,953
Tiredness and fatigue	556	547	46	3	0	106	5	1,263
Failure to thrive	25	33	7	1	0	36	3	105
Bone fracture	13	15	2	1	0	42	6	79
Skeletal deformity	15	26	8	1	0	35	2	87
Numbness or paraesthesia	14	9	1	0	0	7	0	31
Hypocalcaemia	9	2	2	0	0	16	0	29
Seizure or tetany	1	3	0	0	0	16	1	21
Abnormal gait	4	9	1	0	0	6	1	21
Muscle weakness	9	7	2	0	0	3	0	21
Delay in motor development	0	0	0	0	0	5	0	5
Cardiomyopathy	0	0	0	0	0	2	0	2
**Total, n (%)**	1,396 (37.3)	1,420 (36.4)	136 (30.8)	10 (32.3)	0 (0)	614 (15.4)	41 (23.6)	3,617 (29.5)

Wan M, *et al. BMJ Open* 2019; 9:e031870. doi: 10.1136/bmjopen-2019-031870

Supplementary Table S5

Multivariable Poisson regression models of incidence of vitamin D supplementation prescribing in children stratified by sex (complete-case analysis)

	Male	Female
	**IRR[a] (95% CI)**	**IRR[a] (95% CI)**
**Age group**		
Up to 6 months	1.00 (Reference)	1.00 (Reference)
6 months - 4 years	2.33 (1.11 – 4.92)	1.37 (0.73 – 2.56)
5 - 11 years	3.08 (1.47 – 6.49)	2.42 (1.30 – 4.51)
12 - 17 years	5.35 (2.54 – 11.24)	8.15 (4.38 – 15.17)
**Ethnicity**		
White	1.00 (Reference)	1.00 (Reference)
Asian	6.59 (5.92 – 7.33)	6.24 (5.71 – 6.82)
Black	3.67 (3.21 – 4.19)	3.63 (3.26 – 4.05)
Others	3.47 (2.79 – 4.32)	3.79 (3.18 – 4.51)
Mixed	2.32 (1.88 – 2.86)	2.31 (1.94 – 2.75)
**Townsend index**		
1 (least deprived)	1.00 (Reference)	1.00 (Reference)
2	1.03 (0.88 – 1.21)	0.97 (0.85 – 1.11)
3	1.14 (0.98 – 1.33)	1.12 (0.98 – 1.27)
4	1.32 (1.14 – 1.53)	1.31 (1.16 – 1.49)
5 (most deprived)	1.33 (1.13 – 1.56)	1.41 (1.24 – 1.61)
**Country**		
England	1.00 (Reference)	1.00 (Reference)
Wales	0.28 (0.18 – 0.42)	0.31 (0.21 – 0.45)
Scotland	0.31 (0.23 – 0.40)	0.23 (0.17 – 0.30)
Northern Ireland	0.37 (0.23 – 0.61)	0.22 (0.13 – 0.38)
**Year**		
2008	1.00 (Reference)	1.00 (Reference)
2009	2.06 (1.30 – 3.26)	3.03 (1.98 – 4.66)
2010	2.88 (1.87 – 4.45)	4.68 (3.11 – 7.04)
2011	4.70 (3.11 – 7.11)	7.87 (5.30 – 11.68)
2012	9.62 (6.46 – 14.34)	15.19 (10.32 – 22.37)
2013	14.37 (9.68 – 21.32)	20.84 (14.18 – 30.63)
2014	17.62 (11.87 – 26.16)	26.00 (17.69 – 38.22)
2015	18.02 (12.10 – 26.85)	29.35 (19.92 – 43.25)
2016	20.07 (13.43 – 29.98)	31.74 (21.49 – 46.88)

Abbreviations: CI, confidence interval; IRR = incidence rate ratio.
[a]Adjusted for all variables listed in the table. The multilevel models included the general practice as a random effect.

Wan M, *et al. BMJ Open* 2019; 9:e031870. doi: 10.1136/bmjopen-2019-031870

Supplementary Figure S2

Time trends in vitamin D supplementation prescribing in children by product types (n=11,743)

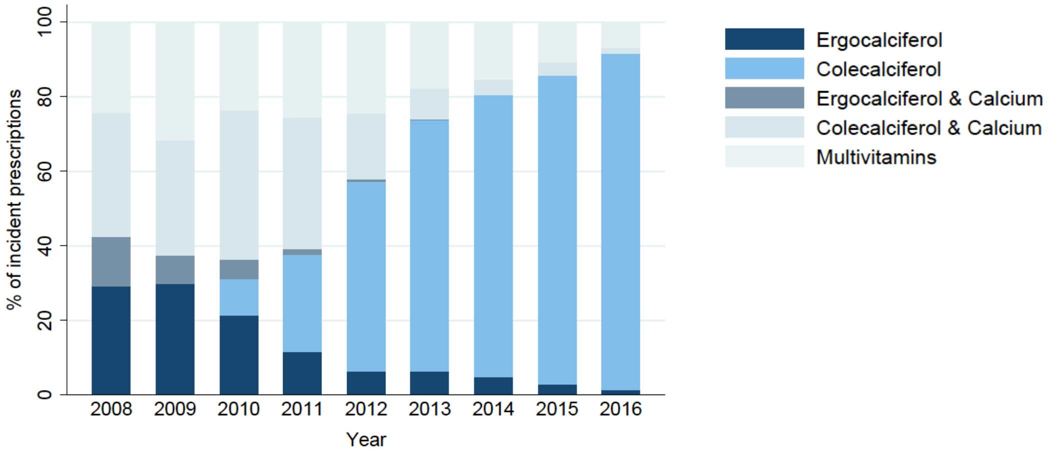

Supplementary Table S6

Doses of vitamin D supplementation prescribed stratified by age group

	**Age group**			
	**Up to 6 months (n = 29)**	**6 months – 4 years (n = 1,670)**	**5 - 11 years (n = 3,504)**	**12 - 17 years (n = 6,540)**
Equivalent daily dose, n (%)	25 (86.2%)	1,460 (87.4%)	3,193 (91.1%)	5,929 (90.7%)
≤400 IU/day	8	707	1,110	640
401 – 1,000 IU/day	1	178	625	2,012
1,001 – 3,000 IU/day	15	170	437	1,073
3,001 – 6,000 IU/day	1	377	869	1,082
6,001 – 10,000 IU/day	0	25	129	1,016
>10,000 IU/day	0	3	23	106
Stoss therapy, n (%)	0	24 (1.7%)	94 (2.7%)	437 (6.7%)
<150,000 IU	0	10	32	86
150,000 – 300,000 IU	0	14	61	290
>300,000 IU	0	0	1	61
Undetermined, n (%)	4 (13.8%)	186 (11.1%)	217 (6.2%)	174 (2.7%)

Wan M, *et al. BMJ Open* 2019; 9:e031870. doi: 10.1136/bmjopen-2019-031870

Supplementary Table S7

Vitamin D supplementation dosing frequencies observed in the study cohort (n=11,743)

Dosing Frequencies	n (%)
Once daily	7,376 (62.8)
Once every 7 days	1,337 (11.4)
Twice a day	1,012 (8.6)
Undetermined	581(5)
Stoss	555 (4.7)
Once 2 times a week	267 (2.3)
Once every 14 days	227 (1.9)
Once 3 times a week	157 (1.3)
Once every 2 days	93 (0.8)
Once every 28 days	41 (0.4)
3 times a day	35 (0.3)
Once every 42 days	19 (0.2)
Others	43 (0.4)

Wan M, *et al. BMJ Open* 2019; 9:e031870. doi: 10.1136/bmjopen-2019-031870
