# Supplementary material for: Vitamin D prescribing in children in UK primary care practices: a population-based cohort study
Source: BMJ Open. 2019 Dec 3;9(12):e031870. doi: 10.1136/bmjopen-2019-031870 (PMC6937102; doi:10.1136/bmjopen-2019-031870)
Supplement: Author's manuscript [file bmjopen-2019-031870.draft_revisions.pdf]

BMJ Open is committed to open peer review. As part of this commitment we make the peer review history of every article we publish publicly available.

When an article is published we post the peer reviewers' comments and the authors' responses online. We also post the versions of the paper that were used during peer review. These are the versions that the peer review comments apply to.

The versions of the paper that follow are the versions that were submitted during the peer review process. They are not the versions of record or the final published versions. They should not be cited or distributed as the published version of this manuscript.

BMJ Open is an open access journal and the full, final, typeset and author-corrected version of record of the manuscript is available on our site with no access controls, subscription charges or pay-per-view fees (http://bmjopen.bmj.com).

If you have any questions on BMJ Open's open peer review process please email info.bmjopen@bmj.com

BMJ Open

Vitamin D prescribing in children in UK primary care practices: a population-based cohort study

Journal:	*BMJ Open*
Manuscript ID	bmjopen-2019-031870
Article Type:	Research
Date Submitted by the Author:	22-May-2019

Complete List of Authors:	Wan, Mandy; Guy's and Saint Thomas' NHS Foundation Trust, Evelina Pharmacy; King's College London, Institute of Pharmaceutical Science<br>Horsfall, Laura; University College London, Research Department of Primary Care and Population Health<br>Basatemur, Emre; University College London, Population, Policy and Practice Programme<br>Patel, Jignesh; King's College London, Institute of Pharmaceutical Science<br>Shroff, Rukshana; Great Ormond Street Hospital For Children NHS Trust,<br>Rait, Greta; University College London, Research Department of Primary Care and Population Health

Keywords:	PAEDIATRICS, Paediatric endocrinology < PAEDIATRICS, EPIDEMIOLOGY

SCHOLARONE™ Manuscripts

Vitamin D prescribing in children in UK primary care practices: a population-based cohort study

Mandy Wan (ORCID ID: 0000-0001-8802-0425), Laura J Horsfall (ORCID ID: 0000-0002-0868-5660), Emre Basatemur, Jignesh P Patel (ORCID ID: 0000-0003-4197-8294), Rukshana Shroff* (ORCID ID: 0000-0001-8501-1072), and Greta Rait* (ORCID ID: 0000-0002-7216-7294)

*These authors share senior authorship

Mandy Wan: Lead Paediatric Research Pharmacist at Guy's and St Thomas' NHS Foundation Trust, Evelina London Children's Hospital, Pharmacy Department, London, UK; and PhD student at Institute of Pharmaceutical Science, King's College London, London, UK.

Laura Horsfall: Senior Epidemiologist and Wellcome Trust Fellow at Research Department of Primary Care and Population Health, University of College London, London, UK.

Emre Basatemur: Clinical Research Fellow at Population, Policy and Practice Programme, University of College London Institute of Child Health, London, UK.

Jignesh P Patel: Reader and Honorary Consultant Pharmacist in Anticoagulation at Institute of Pharmaceutical Science, King's College London, London, UK and Department of Haematological Medicine, King's College Hospital Foundation NHS Trust, London, UK.

Rukshana Shroff: Consultant Paediatric Nephrologist at Renal Unit, Great Ormond Street Hospital NHS Foundation Trust, London, UK.

Greta Rait: Professor of Primary Care and Health Services Research at Research Department of Primary Care and Population Health, University of College London, London, UK.

Corresponding author: Mandy Wan
Email: mandy.wan@gstt.nhs.uk
Organisation: Guy's and St Thomas' NHS Foundation Trust
Evelina London Children's Hospital
Pharmacy Department
Westminster Bridge Road
London
SE1 7EH

For peer review only

Abstract

Objective To examine temporal changes in the incidence and patterns of vitamin D supplementation prescribing between 2008 and 2016.

Design Population-based cohort study.

Setting UK general practice health records from The Health Improvement Network (THIN).

Participants Children aged 0 to 17 years between 2008 and 2016 who were registered with their general practices for at least 3 months.

Outcome measures Annual incidence rates of vitamin D prescriptions were calculated, and rate ratios were estimated using multivariable Poisson regression to explore differences by sociodemographic factors. Data on the type of supplementation, dose, dosing schedule, linked 25-hydroxyvitamin D [25(OH)D] laboratory test results, and clinical symptoms suggestive of vitamin D deficiency were analysed.

Results Among 2 million children, the crude annual incidence of vitamin D prescribing increased by 26-fold between 2008 and 2016 rising from 10.8 (95% CI: 8.9, 13.1) to 276.8 (95% CI: 264.3, 289.9) per 100,000 person-years. Older children, non-white ethnicity, and general practices in England were independently associated with higher rates of prescribing. Analyses of incident prescriptions showed inconsistent supplementation regimens with an absence of recorded pre-supplementation 25(OH)D concentrations in 28.7% to 56.4% of prescriptions annually. There was an increasing trend in prescribing at pharmacological doses irrespective of vitamin D status, deviating in part from UK recommendations. Prescribing at pharmacological doses for children with deficient status increased from 3.8% in 2008 to 79.4% in 2016, but the rise was also observed in children for whom guidelines recommended maintenance doses (0% in 2008 and 53% in 2016). Vitamin D supplementation at pharmacological doses was also prescribed to at least 40% of children with no pre-supplementation 25(OH)D concentrations.

Conclusions There has been a marked and sustained increase in vitamin D supplementation prescribing to children in UK primary care. Current prescribing practices are often unjustified and deviate from national guidelines.

For peer review only - http://bmjopen.bmj.com/site/about/guidelines.xhtml

Strengths and limitations of this study

- This is the first population-based study to examine the extent and variability of vitamin D supplementation prescribing practice using representative primary care data.
- This study does not contain data from secondary care, however, vitamin D deficiency is largely managed in the primary care setting.
- Although several explanations for the factors that might have contributed to changes in vitamin D supplementation prescribing over time are provided, it was not possible to determine the practitioners' rationale for prescribing, nor could we assess clinical appropriateness of prescriptions on an individual level.

For peer review only

Introduction

Vitamin D is an essential prohormone that humans obtain through cutaneous photosynthesis, diet, or supplementation. While few would argue the importance of its physiological role in maintaining calcium homeostasis and bone mineralisation, the beneficial effects of vitamin D supplementation is a topic of much controversy.[1–5] Findings from numerous epidemiological studies have linked low vitamin D status to an increased risk of a myriad of adverse health consequences including diabetes, autoimmune conditions, cancers and infections, among others.[6] Yet, the majority of randomised controlled trials do not show improved outcomes with vitamin D supplementation,[6–9] and meta-analyses are inconclusive and widely debated.[6–15]

Against this backdrop of ongoing debate, vitamin D deficiency is widely prevalent in all age groups and seen as a growing public health concern worldwide.[16] The US,[17] Canada,[18] Australia,[19,20] France,[21] and the UK[22,23] have all reported a significant increase in laboratory vitamin D testing, with as much as a 90-fold increase over a 10-year period.[19,20] In line with this, there has been a marked increase in the annual cost of vitamin D prescriptions from £0.5 to £40 million between 2007 and 2016 in primary care spending in England[24]; a rising trend that was also seen in self-reported use of vitamin D supplements among US adults.[25]

The economic burden of this practice change has not only gained policy makers' attention,[26–28] but also raised questions about the clinical management of vitamin D deficiency. There are numerous international and national guidelines on vitamin D supplementation and health which differ in their definition of vitamin D deficiency and supplementation recommendations.[29–32] Notably, the UK, where there have been at least 10 national guidance on vitamin D in children published between 2007 and 2016, exemplifies this situation.[30–39] While individually they aim to assist practitioners on best practice, having multiple, sometimes conflicting or ambiguous, guidelines is confusing.[32,40–42] Relatively little is known about the prescribing practice of vitamin D, and data in children are scarce. Given the controversies and escalating costs, a comprehensive analysis of vitamin D supplementation offers an

For peer review only - http://bmjopen.bmj.com/site/about/guidelines.xhtml

opportunity to facilitate the appropriate use of vitamin D supplementation for patient benefit and contain healthcare expenditure.

The aim of our study was to examine recent trends in the prescribing pattern of vitamin D supplementation in children. We quantified temporal changes in the incidence of vitamin D prescriptions and examined prescription characteristics, including the type of supplementation, dose, and dosing schedule, in a UK population-based study.

Methods

Data source

We obtained electronic health records from patients registered with UK general practices contributing to The Health Improvement Network (THIN). The THIN database contains anonymised data from 744 general practices of approximately 16 million patients and is broadly representative of the UK population in terms of age, sex, prevalence of medical conditions, and mortality rates.[43] THIN contains information on diagnoses, laboratory tests, symptoms and hospital referrals, as well as basic sociodemographic information. Clinical events and diagnoses are recorded using a hierarchical coding system called Read codes.[44] THIN also includes records of all prescriptions issued and these are linked to the British National Formulary.

Study population

We identified a cohort of children aged 0 to 17 years registered with a THIN general practice from 1st January 2008 to 31st December 2016 (2,138,440 records). Individuals were not eligible to enter the study until three months after their registration with the practice in order to exclude those with prevalent prescriptions.[45] Cohort entry was defined by the latest of: the study start date (1st January 2008), 3 months after the patient's registration with the practice, or the date the practice met two predefined quality indicators (acceptable mortality recording and acceptable computer usage) for electronic data recording (2,069,224 records).[46,47] Children with cystic fibrosis, chronic renal failure, inflammatory bowel diseases or chronic liver diseases were excluded (17,821 records) as these conditions are known

For peer review only - http://bmjopen.bmj.com/site/about/guidelines.xhtml

to affect vitamin D absorption or metabolism and therefore require specialist management. Children were followed until an incident prescription of vitamin D, or censored on death, midpoint of their 18th birthday, end of registration with a practice, the last collection date of data from the practice, or end of the study period (31st December 2016), whichever occurred first. The total number of patient-years between cohort entry and exit defined the denominator for incidence calculations.

Outcome

A vitamin D prescription was defined as a record of either: 1) a single-ingredient product containing calciferol, ergocalciferol or colecalciferol as the only active ingredient; or 2) a combination product containing calciferol, ergocalciferol or colecalciferol with other active ingredients (e.g. ergocalciferol with calcium, multi-vitamin preparations). For the latter, only those that could be linked to a Read code indicating vitamin D deficiency or insufficiency or 25-hydroxyvitamin D [25(OH)D] testing within 90 days either side of the date of the prescription were included. Comprehensive lists of drug codes and Read codes were developed using previously published methodology (Supplementary Table 1 and 2).[48] Incident cases were defined as children with a first ever recording of a vitamin D prescription in THIN during the study period. The date at which the first recording of vitamin D prescription was made was classified as the index date.

Covariates

Age groups were defined as: 0-1, 2-5, 6-11, and 12-17 years. Ethnicity was grouped into the 2011 UK Census 5-caterogry classification; children with multiple ethnicity records belonging to different categories were included in the analysis under the 'missing data' category. Townsend index, an area-based relative measure of material deprivation, was extracted; the value closest to the start of the observation period was used for patients with multiple records.

25-hydroxyvitamin D [25(OH)D] laboratory test results associated with incident prescriptions were identified by temporal proximity. A result was considered to be contemporary and would have formed the basis of prescribing if it occurred 90 days prior to the index date. Based on the elimination half-life

For peer review only - http://bmjopen.bmj.com/site/about/guidelines.xhtml

of 25-hydroxyvitamin D, results recorded between 91-180 days after the index date were used to assess vitamin D status post supplementation. Levels <25 nmol/L were categorised as deficient as per the UK Department of Health and Social Care definition relevant at the time of the study period.[30] Levels were further categorised as insufficient (25-50 nmol/L) as defined by the British Paediatric and Adolescent Bone Group and supported by the British Society of Paediatric Radiology and the Royal College of Paediatrics and Child Health.[31,37,38]

Read codes of symptoms or clinical complications that could potentially be related to vitamin D deficiency within the 90 days prior to the index date were extracted and organised into 12 categories as presented in Supplementary Table S3.

Incident vitamin D prescriptions analyses

Children with multiple vitamin D prescriptions on the index date were excluded from prescription pattern analyses as it was not possible to determine whether all prescriptions were relevant. Prescriptions were categorised according to the type of supplementation as per case definition above. Equivalent daily dose (EDD) was calculated for each incident prescription as determined by the quantity of vitamin D [international unit (IU)] prescribed per dose and the prescribed dosing frequency. Prescriptions with titration dosages (e.g. two tablets daily for 2 weeks and then one tablet monthly) were handled by considering the first dosage only to capture the initial management strategy. Where unit of dosing was missing (e.g. every day), one single dosing unit was assumed for tablet/capsule products only. Generic (e.g. as directed), or ambiguous (e.g. once daily for a liquid product) dosages were categorised under the 'undetermined' category. EDDs were further categorised into dose bands. To assist interpretation, doses of 280 - 400 IU were considered prevention doses across all age groups based on recommendations relevant at the time of the study period.[30,33,36,38] Doses between 1,001 and 10,000 IU/day were considered as pharmacological doses independent of age.[31,34] Prescription quantity supplied in days was calculated based on total prescription quantity issued and corresponding dosage and capped at 365 days to take account of product shelf life. High-dose vitamin D regimens (stoss regimens) prescribed either as intramuscular injections or oral preparations were categorised separately

For peer review only - http://bmjopen.bmj.com/site/about/guidelines.xhtml

and reported as either low dose (total dose <150,000 IU) or high dose (total dose ≥150,000 IU) stoss. As oral stoss regimens may be prescribed as a single dose or spread over several days, prescriptions with explicit duration directions for ≤15 days (e.g. 5,000 IU once daily for 14 days), or where the quantity prescribed were ≤7 days with an equivalent daily dose ≥10,000 IU (e.g. 5 capsules of 20,000 IU with dosing direction "one capsules once daily") were also categorised as stoss.

Statistical analysis

Cohort characteristics are presented as frequencies (%) for categorical data, and medians and interquartile range (IQR) for non-normally distributed continuous data. Data are stratified by gender, ethnicity, socioeconomic quintile, country and calendar year. We calculated crude annual incidence rates by dividing the number of children with a first ever recording of a vitamin D prescription by the Person Years at Risk (PYAR). Confidence intervals (95% CI) were calculated assuming a Poisson distribution. Using Poisson regression, we estimated gender stratified incidence rate ratios with 95% confidence intervals, adjusting for potential confounders. Time period was fitted as both a linear and categorical variable and models compared using the log likelihood ratio test; there was evidence that the categorised time period was a better fit with the data and thus the time period was treated as a categorical variable. A fully adjusted gender stratified model with an interaction between age and ethnicity was fitted and examined using likelihood ratio test; while the interaction term was significant (P<0.01), the quantitative impact of interactions was modest and for this reason was not included in the final model. The final model with the inclusion of the GP practice as a random effect to account for any data clustering was used to calculate adjusted incidence rate ratios (IRRs). Data analysis was conducted using Stata 15 (Stata Corp, College Station, Texas).

Three sensitivity analyses were performed. Firstly, we considered a narrower case definition for vitamin D prescription, where only single-ingredient products were included. Secondly, we repeated the incidence analysis using complete cases only, that is among children who had complete data on gender, age, ethnicity, socioeconomic status, country and calendar year. Finally,–we considered a broader definition for prevention doses, where equivalent daily doses of ≤1,000 IU were included, given

For peer review only - http://bmjopen.bmj.com/site/about/guidelines.xhtml

divergent views exist within the medical community with regards to supplementation strategies for primary prevention.[30,31,33,34]

Patient and public involvement

Patient and parent/carer representatives were involved in developing study plans and approving the lay summaries for the grant supporting this study. Patients were not involved in setting the specific research question, the outcome measure, the design, or implementation of this study. No patients were asked to advise on interpretation or writing up of results. It is not possible to disseminate the results of the research to study participants, but views from patient and parent/carer representatives will be sought in disseminating the research findings.

Ethics

The THIN data collection scheme to obtain and provide anonymised patient data was approved by the National Health Service South-East Multicentre Research Ethics Committee (REC Reference: 04/MRE01/9) in 2002. The study protocol was approved by an independent Scientific Review Committee (Reference: 16THIN035).

Results

A total of 2,051,403 eligible children from 723 general practices contributed data between Jan 1, 2008 and Dec 31, 2016, giving a total of 8 million person-years of follow-up. Of those children, 12,277 had an incident vitamin D prescription during the study period. Patient characteristics stratified by gender, ethnicity, country of general practice, and socioeconomic status are shown in the Table 1 for the overall cohort and the incident cohort.

Crude incidence

The crude annual incidence of vitamin D prescribing in children increased by 26-fold between 2008 and 2016 rising from 10.8 (95% CI: 8.9, 13.1) to 276.8 (95% CI: 264.3, 289.9) per 100,000 person-years. Figure 1 shows temporal trends in vitamin D prescribing in children alongside key national

For peer review only - http://bmjopen.bmj.com/site/about/guidelines.xhtml

guidelines or reports on vitamin D deficiency.[30–39,49] Prescribing rates for females were higher than males, with an average ratio of male to female prescribing rate of 1:1.5 across all time periods. When vitamin D prescription was defined using single-ingredient vitamin D products only, we found similar trends to the main analysis (Figure 1).

Adjusted incidence rates

In multivariable analysis, increasing age, non-white ethnicity, and social deprivation were associated with higher incidence rates of vitamin D prescribing (Table 2). Among children aged 6-11 and 12-17 years, gender difference in incidence rate ratios was observed, with 1.3-fold and 2.5-fold higher vitamin D supplementation in girls than boys, respectively. General practices in England were also associated with higher incidence rate ratios as compared to Wales, Scotland and Northern Ireland. For both males and females, adjusted incidence rate ratios were higher than their corresponding crude values, with a 32-fold and 39-fold increase in the incidence of vitamin D prescribing between 2008 and 2016, respectively. A sensitivity analysis using complete cases only was comparable to the main analysis, albeit the magnitude of the effects of ethnicity was greater for both genders, and a smaller temporal difference in annual incidence rates was seen in males.

25-hydroxyvitamin D testing and presence of symptoms at initiation of supplementation

25(OH)D levels recorded before starting vitamin D supplementation are presented in Figure 2A. Overall, 28.7 to 56.4% of prescriptions annually had no linked 25(OH)D results recorded in the 3 months prior to the index date. The proportion of children with laboratory confirmed vitamin D deficiency increased from 25.7% to 40.9% between 2008 and 2011 before showing a gradual decrease to 27.2% in 2016, coinciding with the issue of public health guidelines on primary prevention of vitamin D deficiency for at risk individuals. Over the study period, increased prescribing of vitamin D supplementation was observed in children with insufficient status, increasing from 15.8 - 21.7% between 2008 and 2011 to 27.9 - 37.1% during the period between 2012 to 2016.

For peer review only - http://bmjopen.bmj.com/site/about/guidelines.xhtml

29.5% of children had symptoms related to vitamin D deficiency (Supplementary Table S3). The most commonly recorded symptoms were those related to musculoskeletal or non-specific pain, tiredness or fatigue. In the group of children with no recorded pre-supplementation 25(OH)D levels, only 15.4% had symptoms.

Formulations

Overall, 109 different pharmaceutical formulations of at least 32 dosage strengths (e.g. 3,000 IU/mL, 20,000 IU/tablet) were prescribed. A temporal change in supplementation form was observed; colecalciferol products accounted for 9.9% of the prescriptions in 2010 and increased to 90.2% in 2016, explaining the trends in prescribing patterns shown in Figure 1 (Supplementary Figure S1).

Doses and frequencies of administration

The EDD prescribed ranged from 27 to 160,000 IU/day (prescribed doses stratified by age group is summarised in Supplementary Table S4) with a median duration of supply of 56 days (IQR: 30, 83 days). There were 237 (2.0%) prescriptions with doses less than the lowest recommended primary prevention dose (<280 IU/day), and 132 (1.1%) prescriptions with doses above the highest recommended pharmacological dose (>10,000 IU/day). A total of 24 different dosing frequencies were noted, with 37.2% of children on regimens other than once daily dosing (Supplementary Table S5).

Trend analysis of supplementation regimens stratified by pre-supplementation 25(OH)D levels is presented in Figure 3. Among children with deficiency, prescriptions with EDD >1,000 IU/day, considered as pharmacological doses, showed a yearly increased from 3.8% in 2008 to 79.4% in 2016, in line with recommendations. These temporal changes were also found among children with insufficient status; the corresponding temporal decrease of prescriptions at prevention doses was noted irrespective of the cut-off value used to define prevention doses (≤400 or ≤1,000 IU). Overall, vitamin D supplementation at pharmacological doses (>1,000 IU/day or stoss) was prescribed to 42.8% of children with insufficient status, outside of UK recommendations.

For peer review only - http://bmjopen.bmj.com/site/about/guidelines.xhtml

25-hydroxyvitamin D testing at follow-up

In the first 90 days post initiation of vitamin D supplementation, 9% (n=1,109) of children had 25(OH)D levels recorded, of which 24.9% were recorded within 1 month after the index date, outside of guideline recommendations. Post-supplementation testing at the recommended time intervals was low; 25(OH)D levels in the period between 91 to 180 days after incident prescriptions were available for only 8.4% (n=1,035) of children (Figure 2B). Over the study period, the proportion of children with post-supplementation 25(OH)D levels >50 nmol/L increased from 11.1% to 76.3%.

For peer review only

Discussion

In a large cohort representative of children in UK primary care, there has been a marked temporal increase in vitamin D prescribing, even after adjustment for changes in population demographics. The adjusted incidence rates increased by 32-fold and 39-fold between 2008 and 2016 for males and females, respectively. Vitamin D levels for guiding supplementation were not available in over 30% of children annually, and an increasing trend in prescribing vitamin D supplementation at pharmacological doses irrespective of vitamin D status was noted with wide variations in supplementation regimens prescribed, deviating in part from UK recommendations. Furthermore, with no clear evidence to support the use of vitamin D supplementation on health outcomes, other than for the prevention or treatment of rickets and osteomalacia, this unjustified and excessive prescribing practice incurs a huge, potentially avoidable, expense to our health care system.

This is the first study to report UK estimates of incidence rates of vitamin D prescribing in children using a comprehensive case definition for vitamin D prescription. An earlier study based in England included only 156 general practices and reported a marked increase in the diagnosis of vitamin D deficiency and prescription costs of single-ingredient vitamin D products in children between 2000 and 2014.[50,51] Our study reports similar trends but provides data from a much larger number of practices across the whole of the UK, with detailed socio-demographic prescribing patterns and extending the data to 2016.

For peer review only - http://bmjopen.bmj.com/site/about/guidelines.xhtml

Vitamin D prescribing in children increased sharply between 2011 and 2014, coinciding with the publication of guidelines and reports from the UK government and national guideline committees that emphasise the importance of vitamin D supplementation in certain groups of vulnerable children and young people.[30–39] These have, in part, raised awareness of vitamin D deficiency among primary care practitioners, and explain the increase in vitamin D prescribing practices. The observed changed in the type of supplementation prescribed, from ergocalciferol and combination products to colecalciferol products, is congruent with increase product availability of the latter[24] and may potentially also relate to data suggesting colecalciferol is more efficacious at raising serum 25(OH)D concentrations than ergocalciferol.[52]

Patient age, gender and ethnicity were identified as drivers for prescribing, in keeping with the higher prevalence rates of vitamin D deficiency reported in teenage girls, and non-white ethnic groups in the UK.[33] Interestingly, we also observed increased rates of prescribing among practices in England as compared to those in the devolved nations, suggesting other contextual factors influencing practitioners' prescribing behaviour; disparities may be due to variations in regional strategies in implementing national guidelines, or differences in practitioners' experience and attitudes towards vitamin D deficiency, and require further exploration.[40,42,53]

The initiation of vitamin D supplementation should be guided by 25(OH)D levels, except when used for primary prevention, as indicated in guideline recommendations.[31,34] Yet, our results show an absence of laboratory confirmed vitamin D status in over 30% of prescriptions annually since 2009, a practice also reported by others.[42] It is possible that a proportion of children may have 25(OH)D levels checked and recorded by hospital clinicians; although children with conditions known to affect vitamin D absorption and metabolism and thus likely to receive secondary input were already excluded in our analysis. We also recognised that a proportion of these prescriptions may also have been intended for primary prevention. However, applying the most conservative primary prevention dose definition of ≤1,000 IU/day, our finding that at least 40% of prescriptions exceeded 1,000/day, and together with

For peer review only - http://bmjopen.bmj.com/site/about/guidelines.xhtml

only a small number of children with recorded symptoms possibly related to vitamin D deficiency, would be suggestive of aberrant prescribing.

Our analysis also found an upward trend in prescribing of vitamin D supplementation at pharmacological doses independent of vitamin D status. To some extent, this pattern observed for children with deficiency suggests that practitioners are responsive to prescribing guidelines[31,34], at least on a population level. On the other hand, a comparable increase was also seen in the prescription of pharmacological doses of vitamin D to children with vitamin D insufficiency; this is not recommended in UK guidelines, and perhaps reflects confusion arising from multiple, sometimes conflicting, recommendations. At the same time, these deviations from recommendations might suggest a somewhat less cautious attitude towards the use of higher than recommended doses of vitamin D among practitioners. Vitamin D has a wide therapeutic window and risk of toxicity is low, nevertheless the potential risk of vitamin D toxicity exists and requires further exploration given its widespread, and sometimes unregulated use.

The major strength of our study is the large sample size that is representative of real-life clinical practice across the UK. Prescribing details in THIN are well recorded because all prescriptions from general practice are generated via a standardised computerised system. The inclusion of combination vitamin D products in the case definition also captures real life clinical practice, but some cases could be missed if combination vitamin D product was prescribed but a diagnosis of vitamin D status not entered in the primary care record. There are some limitations to our study too. The first vitamin D prescription recorded in THIN may not be the first ever prescription issued to the patient as initial prescribing may have occurred in secondary care and continued in general practice. However, most prescriptions issued by UK hospitals are limited to 7-14 days for clinically urgent conditions only, so the analysed sample is very likely to be representative of true practice. The study design did not allow us to determine the practitioners' rationale for prescribing, nor could we assess clinical appropriateness of prescriptions on an individual level. Despite these limitations, our study explored the major clinical indicator for vitamin D supplementation, namely 25(OH)D levels and presence of symptoms.

For peer review only - http://bmjopen.bmj.com/site/about/guidelines.xhtml

Our study highlights two key implications for practice. Firstly, diverse clinical practice highlights the need for a broader understanding of the multitude factors influencing general practitioners' prescribing decisions for vitamin D supplementation in order to reduce inappropriate prescribing.[40,53] Further guidelines alone, even in the absence of ambiguity, are unlikely to be effective. Secondly, vitamin D supplementation exceeding recommended doses, and arguably, prescriptions at prevention doses may not be the best use of the limited health resources. On this basis, at least 32.3% of our prescriptions would fall into this category. Using the most conservative estimate from an earlier study[51] (£1.65 million for the total direct cost of vitamin D prescriptions in children per year), and without costing for consultation time with health care professionals, dispensing or tests, we estimate an annual saving of £0.5 million. This may not be considered a sizeable financial burden in view of the total UK health spending, but if replicated in adults, this would amount to a considerable amount given the annual spending on single-ingredients vitamin D products alone in England was reported to be £40 million in 2016.

Conclusion

There has been a marked and sustained increase in vitamin D prescribing to children in primary care in the UK. While this may reflect the success of the Department of Health and Social Care's efforts in raising awareness of vitamin D deficiency, findings from our study would suggest that vitamin D prescribing practices among general practitioners is not in line with UK recommendations, in terms of the number of patients treated, the doses used for supplementation, as well as the practice of prescribing vitamin D without appropriate testing. More recent NHS England guidance[27], that recommends prescribing pharmacological doses of vitamin D supplementation only to those diagnosed with vitamin D deficiency, has the potential to reduce unnecessary prescriptions and NHS expenditure, but its impact on prescribing practice would require further evaluation as previous guidelines have not always translated into practice.

For peer review only - http://bmjopen.bmj.com/site/about/guidelines.xhtml

Contributors

MW, GR, RS and JP were involved in the conception of the study and obtaining funding. All authors contributed to the study protocol and interpretation of data. MW had full access to the database, programming code and performed the data extraction and analysis. MW drafted the manuscript which all authors contributed to and revised critically before approval of the final manuscript. MW is the guarantor. The corresponding author attests that all listed authors meet authorship criteria and that no others meeting the criteria have been omitted.

Funding

This study was funded by a Clinical Doctoral Research Fellowship grant (ICA-CDRF-2016-02-057) from the UK National Institute for Health Research (NIHR). MW is a doctoral student supported by this grant. Independent expert peer reviewers provided feedback on the grant application underpinning this study but had no further role in study design, data collection, analysis, interpretation, or drafting of the manuscript. LH is fully funded by the Wellcome Trust (209207/Z/17/Z). RS holds a Career Development Fellowship with the National Institute for Health Research. A part of the work took place in the Biomedical Research Centre at Great Ormond Street Hospital for Children NHS Foundation Trust and University College London. The views expressed are those of the authors and not necessarily those of the NHS, the NIHR, or the Department of Health and Social Care.

Competing interests

All authors have completed the ICMJE uniform disclosure form at www.icmje.org/coi_disclosure.pdf and declare: no support from any organisation for the submitted work; no financial relationships with any organisations that might have an interest in the submitted worked in the previous three years; no other relationships or activities that could appear to have influenced the submitted work

Data sharing

The General Practices in The Health Improvement Network (THIN) is run by the Department of Health and Social Care. All the data are available via an application to THIN. Data acquisition is associated with a fee.

For peer review only - http://bmjopen.bmj.com/site/about/guidelines.xhtml

References

1 Boucher BJ. Causal link between vitamin D deficiency and ill health still possible. *BMJ* 2014; 348: g2923.

2 Welsh P, Sattar N. Vitamin D and chronic disease prevention. *BMJ* 2014; 348: g2280.

3 Stokes CS, Lammert F. Vitamin D supplementation: less controversy, more guidance needed. *F1000Res* 2016; 5(F1000 Faculty Rev):2017.

4 Bischoff-Ferrari HA, Orav EJ, Abderhalden L, Dawson-Hughes B, Willett WC. Vitamin D supplementation and musculoskeletal health. *The Lancet Diabetes & Endocrinology* 2019; 7: 85.

5 Bolland MJ, Grey A, Avenell A. Vitamin D supplementation and musculoskeletal health – Authors' reply. *The Lancet Diabetes & Endocrinology* 2019; 7: 88–89.

6 Theodoratou E, Tzoulaki I, Zgaga L, Ioannidis JPA. Vitamin D and multiple health outcomes: umbrella review of systematic reviews and meta-analyses of observational studies and randomised trials. *BMJ* 2014; 348: g2035.

7 Winzenberg TM, Powell S, Shaw KA, Jones G. Vitamin D supplementation for improving bone mineral density in children. *Cochrane Database of Systematic Reviews* 2010; 10: CD006944.

8 Autier P, Mullie P, Macacu A, Dragomir M, Boniol M, Coppens K *et al.* Effect of vitamin D supplementation on non-skeletal disorders: a systematic review of meta-analyses and randomised trials. *The Lancet Diabetes & Endocrinology* 2017; 5: 986–1004.

9 Bolland MJ, Grey A, Avenell A. Effects of vitamin D supplementation on musculoskeletal health: a systematic review, meta-analysis, and trial sequential analysis. *The Lancet Diabetes & Endocrinology* 2018; 6: 847–858.

10 Bischoff-Ferrari HA, Willett WC, Wong JB, Giovannucci E, Dietrich T, Dawson-Hughes B. Fracture prevention with vitamin D supplementation: a meta-analysis of randomized controlled trials. *JAMA* 2005; 293: 2257–2264.

11 Jolliffe DA, Griffiths CJ, Martineau AR. Vitamin D in the prevention of acute respiratory infection: Systematic review of clinical studies. *The Journal of Steroid Biochemistry and Molecular Biology* 2013; 136: 321–329.

12 Martineau AR, Jolliffe DA, Hooper RL, Greenberg L, Aloia JF, Bergman P *et al.* Vitamin D supplementation to prevent acute respiratory tract infections: systematic review and meta-analysis of individual participant data. *BMJ* 2017; 356: i6583.

13 World Health Organisation. Vitamin D for prevention of respiratory tract infections. WHO. http://www.who.int/elena/titles/commentary/vitamind_pneumonia_children/en/ (accessed Feb 28, 2019).

14 Jolliffe DA, Greenberg L, Hooper RL, Griffiths CJ, Camargo CA, Kerley CP *et al.* Vitamin D supplementation to prevent asthma exacerbations: a systematic review and meta-analysis of individual participant data. *The Lancet Respiratory Medicine* 2017; 5: 881–890.

15 Martineau A. Vitamin D supplementation to prevent asthma exacerbations – Authors' reply. *The Lancet Respiratory Medicine* 2018; 6: e26–e27.

16 Palacios C, Gonzalez L. Is vitamin D deficiency a major global public health problem? *J Steroid Biochem Mol Biol* 2014; 144PA: 138–145.

For peer review only

17 Shahangian S, Alspach TD, Astles JR, Yesupriya A, Dettwyler WK. Trends in laboratory test volumes for Medicare Part B reimbursements, 2000-2010. *Arch Pathol Lab Med* 2014; 138: 189–203.

18 Ontario Health Technology Advisory Committee. Clinical Utility of Vitamin D Testing. *Ont Health Technol Assess Ser* 2010; 10: 1–93.

19 Bilinski KL, Boyages SC. The rising cost of vitamin D testing in Australia: time to establish guidelines for testing. *The Medical Journal of Australia* 2012; 197: 90.

20 Bilinski K, Boyages S. Evidence of overtesting for vitamin D in Australia: an analysis of 4.5 years of Medicare Benefits Schedule (MBS) data. *BMJ Open* 2013; 3: e002955.

21 Caillet P, Goyer-Joos A, Viprey M, Schott A-M. Increase of vitamin D assays prescriptions and associated factors: a population-based cohort study. *Scientific Reports* 2017; 7: 10361.

22 Sattar N, Welsh P, Panarelli M, Forouhi NG. Increasing requests for vitamin D measurement: costly, confusing, and without credibility. *The Lancet* 2012; 379: 95–96.

23 Zhao S, Gardner K, Taylor W, Marks E, Goodson N. Vitamin D assessment in primary care: changing patterns of testing. *London J Prim Care (Abingdon)* 2015; 7: 15–22.

24 NHS Digital. Prescription Cost Analysis. NHS Digital. https://digital.nhs.uk/data-and-information/publications/statistical/prescription-cost-analysis (accessed Feb 28, 2019).

25 Kantor ED, Rehm CD, Du M, White E, Giovannucci EL. Trends in Dietary Supplement Use Among US Adults From 1999-2012. *JAMA* 2016; 316: 1464–1474.

26 *Vitamin D Testing in the General Population: A Review of the Clinical and Cost-Effectiveness and Guidelines*. Canadian Agency for Drugs and Technologies in Health: Ottawa (ON), 2015. http://www.ncbi.nlm.nih.gov/books/NBK274106/ (accessed Feb 28, 2019).

27 NHS England. Conditions for which over the counter items should not routinely be prescribed in primary care: Guidance for CCGs. 2018. https://www.england.nhs.uk/wp-content/uploads/2018/03/otc-guidance-for-ccgs.pdf (accessed Feb 28, 2019).

28 Boyages SC. Vitamin D testing: new targeted guidelines stem the overtesting tide. *Med J Aust* 2016; 204: 18.

29 Rosen CJ, Abrams SA, Aloia JF, Brannon PM, Clinton SK, Durazo-Arvizu RA *et al.* IOM Committee Members Respond to Endocrine Society Vitamin D Guideline. *J Clin Endocrinol Metab* 2012; 97: 1146–1152.

30 Scientific Advisory Committee on Nutrition. Update on Vitamin D - Position statement by the Scientific Advisory Committee on Nutrition. 2007. https://assets.publishing.service.gov.uk/government/uploads/system/uploads/attachment_data/file/339349/SACN_Update_on_Vitamin_D_2007.pdf (accessed Feb 28, 2019).

31 Royal College of Paediatrics and Child Health. Guide for Vitamin D in Childhood. 2013. https://www.rcpch.ac.uk/sites/default/files/2018-03/guide_to_vitamin_d_in_childhood.pdf. (accessed Feb 28, 2019).

32 National Institute for Health and Care Excellence. Vitamin D: supplement use in specific population groups (PH56). 2014. https://www.nice.org.uk/guidance/ph56/resources/vitamin-d-supplement-use-in-specific-population-groups-pdf-1996421765317 (accessed Feb 28, 2019).

For peer review only

33 Scientific Advisory Committee on Nutrition. SACN vitamin D and health report. 2016.

34 National Osteoporosis Society. Vitamin D and Bone Health: A Practical Clinical Guideline for Management in Children and Young People. 2015. https://nos.org.uk/media/2074/vitamin-d-and-bone-health-children.pdf (accessed Feb 28, 2019).

35 National Institute for Health and Care Excellence. Improving the nutrition of pregnant and breastfeeding mothers and children in low-income households. (PH11). 2008. https://webarchive.nationalarchives.gov.uk/20080912094204/http://www.gserve.nice.org.uk/nicemedia/pdf/PH011guidance.pdf (accessed Feb 28, 2019).

36 UK Chief Medical Officers. Vitamin D - advice on supplements for at risk groups - letter from UK Chief Medical Officers. 2012. https://www.gov.uk/government/publications/vitamin-d-advice-on-supplements-for-at-risk-groups (accessed Feb 28, 2019).

37 Arundel P, Ahmed SF, Allgrove J, Bishop NJ, Burren CP, Jacobs B *et al.* British Paediatric and Adolescent Bone Group's position statement on vitamin D deficiency. *BMJ* 2012; 345: e8182.

38 Royal College of Paediatrics and Child Health. Vitamin D: position statement. 2012.https://www.rcpch.ac.uk/resources/vitamin-d-position-statement (accessed Feb 28, 2019).

39 National Institute for Health and Care Excellence Clinical Knowledge Summaries. Vitamin D deficiency in children. 2016https://cks.nice.org.uk/vitamin-d-deficiency-in-children#!references (accessed Feb 28, 2019).

40 Epling JW, Mader EM, Roseamelia CA, Morley CP. Emerging Practice Concerning Vitamin D in Primary Care. *Qual Health Res* 2015; 25: 1005–1012.

41 Cashman KD, Kiely M. Nutrition: New guidelines on vitamin D-ficiency—clear or confusing? *Nature Reviews Endocrinology* 2011; 7: 566–568.

42 DelGiudice NJ, Street N, Torchia RJ, Sawyer SS, Bernard SA, Holick MF. Vitamin D Prescribing Practices in Primary Care Pediatrics: Underpinnings From the Health Belief Model and Use of Web-Based Delphi Technique for Instrument Validity. *Journal of Pediatric Health Care* 2018; 32: 536–547.

43 Blak B, Thompson M, Dattani H, Bourke A. Generalisability of The Health Improvement Network (THIN) database: demographics, chronic disease prevalence and mortality rates. *Journal of Innovation in Health Informatics* 2011; 19: 251–255.

44 Chisholm J. The Read clinical classification. *BMJ* 1990; 300: 1092.

45 Lewis JD, Bilker WB, Weinstein RB, Strom BL. The relationship between time since registration and measured incidence rates in the General Practice Research Database. *Pharmacoepidem Drug Safe* 2005; 14: 443–451.

46 Maguire A, Blak BT, Thompson M. The importance of defining periods of complete mortality reporting for research using automated data from primary care. *Pharmacoepidem Drug Safe* 2009; 18: 76–83.

47 Horsfall L, Walters K, Petersen I. Identifying periods of acceptable computer usage in primary care research databases. *Pharmacoepidemiol Drug Saf* 2013; 22: 64–69.

48 Davé S, Petersen I. Creating medical and drug code lists to identify cases in primary care databases. *Pharmacoepidem Drug Safe* 2009; 18: 704–707.

For peer review only - http://bmjopen.bmj.com/site/about/guidelines.xhtml

49 Department of Health & Food Standards Agency, Department of Health & Food Standards Agency. National Diet and Nutrition Survey. Headline results from Years 1 and 2 (combined) of the Rolling Programme (2008/2009 – 2009/10). 2011. https://www.gov.uk/government/publications/national-diet-and-nutrition-survey-headline-results-from-years-1-and-2-combined-of-the-rolling-programme-2008-9-2009-10 (accessed Feb 28, 2019).

50 Basatemur E, Horsfall L, Marston L, Rait G, Sutcliffe A. Trends in the Diagnosis of Vitamin D Deficiency. *Pediatrics* 2017; 139: e20162748.

51 Basatemur E, Hunter R, Horsfall L, Sutcliffe A, Rait G. Costs of vitamin D testing and prescribing among children in primary care. *Eur J Pediatr* 2017; 176: 1405–1409.

52 Tripkovic L, Lambert H, Hart K, Smith CP, Bucca G, Penson S *et al.* Comparison of vitamin D2 and vitamin D3 supplementation in raising serum 25-hydroxyvitamin D status: a systematic review and meta-analysis123. *Am J Clin Nutr* 2012; 95: 1357–1364.

53 Rockwell M, Kraak V, Hulver M, Epling J. Clinical Management of Low Vitamin D: A Scoping Review of Physicians' Practices. *Nutrients* 2018; 10: 493.

For peer review only

Table 1: Descriptive characteristics of the overall study cohort and incident cohort

	**Overall cohort (n=2,051,403)**	**Incident cohort (n=12,277)**
Median age at entry, years (IQR)	5.5 (0.8 – 11.5)	7.6 (3.5 – 10.5)
Follow up time, years (IQR)	3.5 (1.5 – 6.3)	4.2 (2 – 6.1)
Gender, n (%)		
Male	1,055,395 (51.5)	4,936 (40.2)
Female	996,008 (48·6)	7,341 (59·8)
Ethnicity, n (%)		
White	878,360 (42.8)	2,836 (23.1)
Asian/Asian British	76,840 (3.8)	2,928 (23.9)
Black/African/Caribbean/Black British	47,206 (2.3)	1,275 (10.4)
Other ethnic group	17,656 (0.9)	312 (2.5)
Mixed/Multiple ethnic group	28,605 (1.4)	295 (2.4)
Missing	1,002,736 (48.9)	4,631 (37.7)
Country of GP practice, n (%)		
England	1,451,923 (70.8)	10,921 (88.9)
Northern Ireland	84,171 (4.1)	177 (1.4)
Scotland	310,142 (15.1)	597 (4.9)
Wales	205,167 (10)	582 (4.7)
Townsend deprivation index quintile, n (%)		
1 (least deprived)	397,057 (19.4)	1,452 (11.8)
2	357,567 (17.4)	1,377 (11.2)
3	396,234 (19.3)	2,128 (17.3)
4	379,485 (18.5)	2,740 (22.3)
5 (most deprived)	291,502 (14.2)	2,866 (23.3)
Missing	229,558 (11.2)	1,714 (14)

IQR = Interquartile range; Children with no recorded data on ethnicity or Townsend index were categorised as ‘missing’.

For peer review only

Table 2: Multivariable Poisson regression models of incidence of vitamin D supplementation prescribing in children stratified by gender.

	Male	Female
	**IRR[a] (95% CI)**	**IRR[a] (95% CI)**
**Age group**		
0 - 1 year	1.00 (Reference)	1.00 (Reference)
2 - 5 years	1.56 (1.36 – 1.79)	1.40 (1.21 – 1.63)
6 - 11 years	1.76 (1.54 – 2.01)	2.39 (2.08 – 2.74)
12 - 17 years	2.93 (2.57 – 3.33)	7.37 (6.45 – 8.42)
**Ethnicity**		
White	1.00 (Reference)	1.00 (Reference)
Asian	5.05 (4.60 – 5.54)	4.45 (4.12 – 4.81)
Black	2.83 (2.52 – 3.18)	2.82 (2.57 – 3.09)
Others	2.68 (2.20 – 3.25)	2.71 (2.32 – 3.17)
Mixed	1.93 (1.59 – 2.33)	1.85 (1.57 – 2.16)
Missing	1.49 (1.38 – 1.62)	1.41 (1.32 – 1.51)
**Townsend index**		
1 (least deprived)	1.00 (Reference)	1.00 (Reference)
2	1.03 (0.92 – 1.17)	1.03 (0.94 – 1.14)
3	1.19 (1.06 – 1.33)	1.20 (1.09 – 1.32)
4	1.37 (1.22 – 1.53)	1.37 (1.25 – 1.51)
5 (most deprived)	1.41 (1.25 – 1.59)	1.45 (1.31 – 1.60)
Missing	1.38 (1.19 – 1.61)	1.44 (1.27 – 1.63)
**Country**		
England	1.00 (Reference)	1.00 (Reference)
Wales	0.34 (0.25 – 0.46)	0.33 (0.25 – 0.46)
Scotland	0.24 (0.19 – 0.32)	0.18 (0.14 – 0.23)
Northern Ireland	0.24 (0.16 – 0.37)	0.17 (0.11 – 0.27)
**Year**		
2008	1.00 (Reference)	1.00 (Reference)
2009	2.47 (1.74 – 3.51)	2.61(1.93 – 3.52)
2010	3.69 (2.64 – 5.16)	4.97 (3.75 – 6.58)
2011	6.72 (4.88 – 9.25)	8.04 (6.13 – 10.56)
2012	14.61 (10.72 – 19.90)	16.16 (12.39 – 2.08)
2013	21.09 (15.52 – 28.66)	22.81 (17.52 – 29.70)
2014	25.80 (18.98 – 35.07)	28.11 (21.58 – 36.62)
2015	29.18 (21.43 – 39.75)	33.49 (25.67 – 43.69)
2016	32.26 (23.64 – 44.03)	38.83 (29.71 – 50.74)

Abbreviations: CI, confidence interval; IRR = incidence rate ratio.
[a]Adjusted for all variables listed in the table. The multilevel models included the general practice as a random effect.

For peer review only

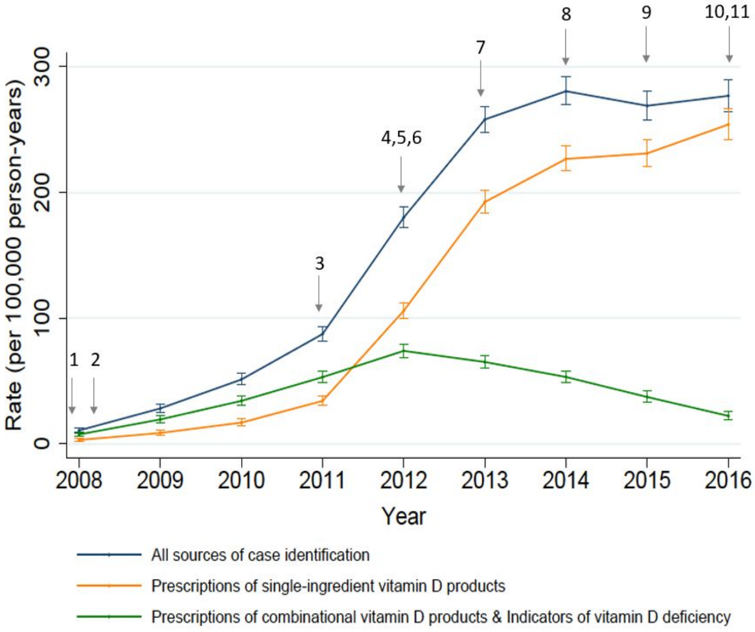

Figure 1: Time trends in vitamin D supplementation prescribing in children using all components of the case definition together and each source of case identification independently. Crude incidence rates with 95% confidence intervals represented by vertical bars.

Key: SACN = Scientific Advisory Committee on Nutrition; NICE = The National Institute for Health and Care Excellence; RCPCH = Royal College of Paediatrics and Child Health; NOS = National Osteoporosis Society

1) 2007: SACN: Update on Vitamin D[30]
2) 2008: NICE: Improving the nutrition of pregnant and breastfeeding mothers and children in low-income households[35]
3) 2011 - The National Diet and Nutrition Survey (NDNS) rolling programme: Data from years 1 & 2[49]
4) 2012: Department of Health: Vitamin D - advice on supplements for at risk groups[36]
5) 2012: British Paediatric and Adolescent Bone Group's position statement on vitamin D deficiency[37]
6) 2012: RCPCH: Vitamin D: Position statement[38]
7) 2013: RCPCH: Guide for Vitamin D in Childhood[31]
8) 2014: NICE: Vitamin D: increasing supplement use in at-risk groups[32]
9) 2015: NOS: Vitamin D and Bone Health[34]
10) 2016: SACN: vitamin D and health report[33]
11) 2016: NICE Clinical Knowledge Summaries: Vitamin D deficiency in children[39]

For peer review only

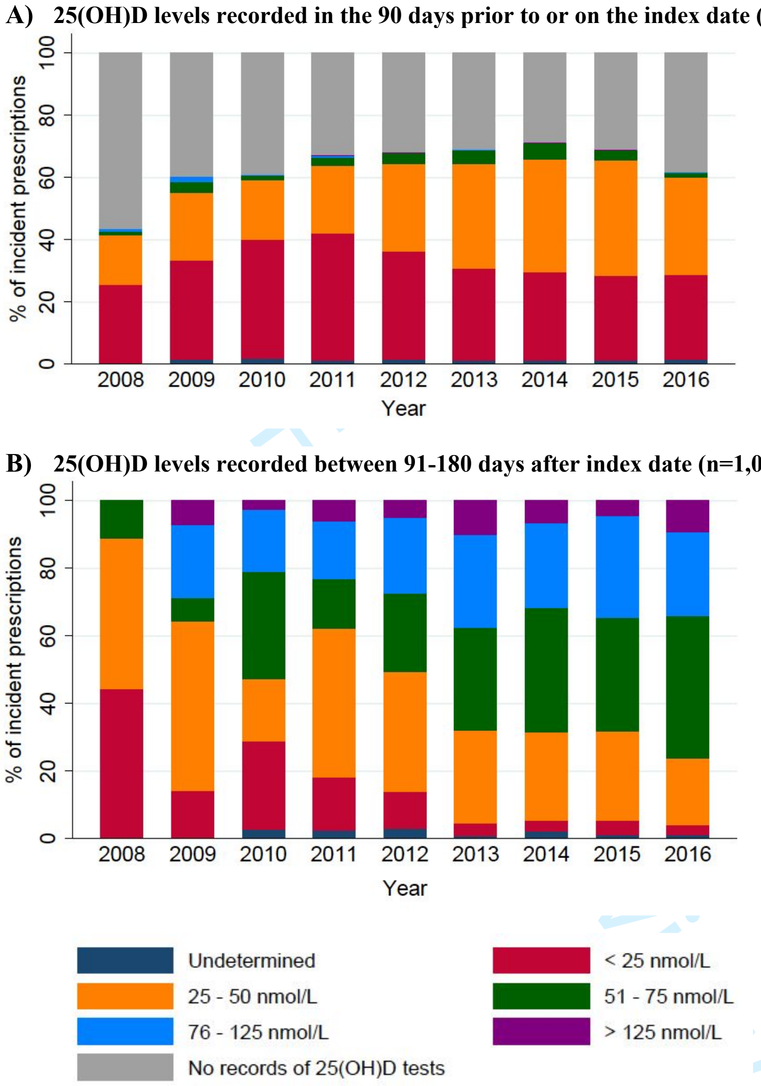

Figure 2: A) 25-hydroxyvitamin D levels recorded in the 90 days prior to or on the index date (n=12,277); B) 25-hydroxyvitamon D levels recorded between 91-180 days after index date (only children with known levels, n=1,035). Records with ambiguous unit of measurements were categorised as 'undetermined'.

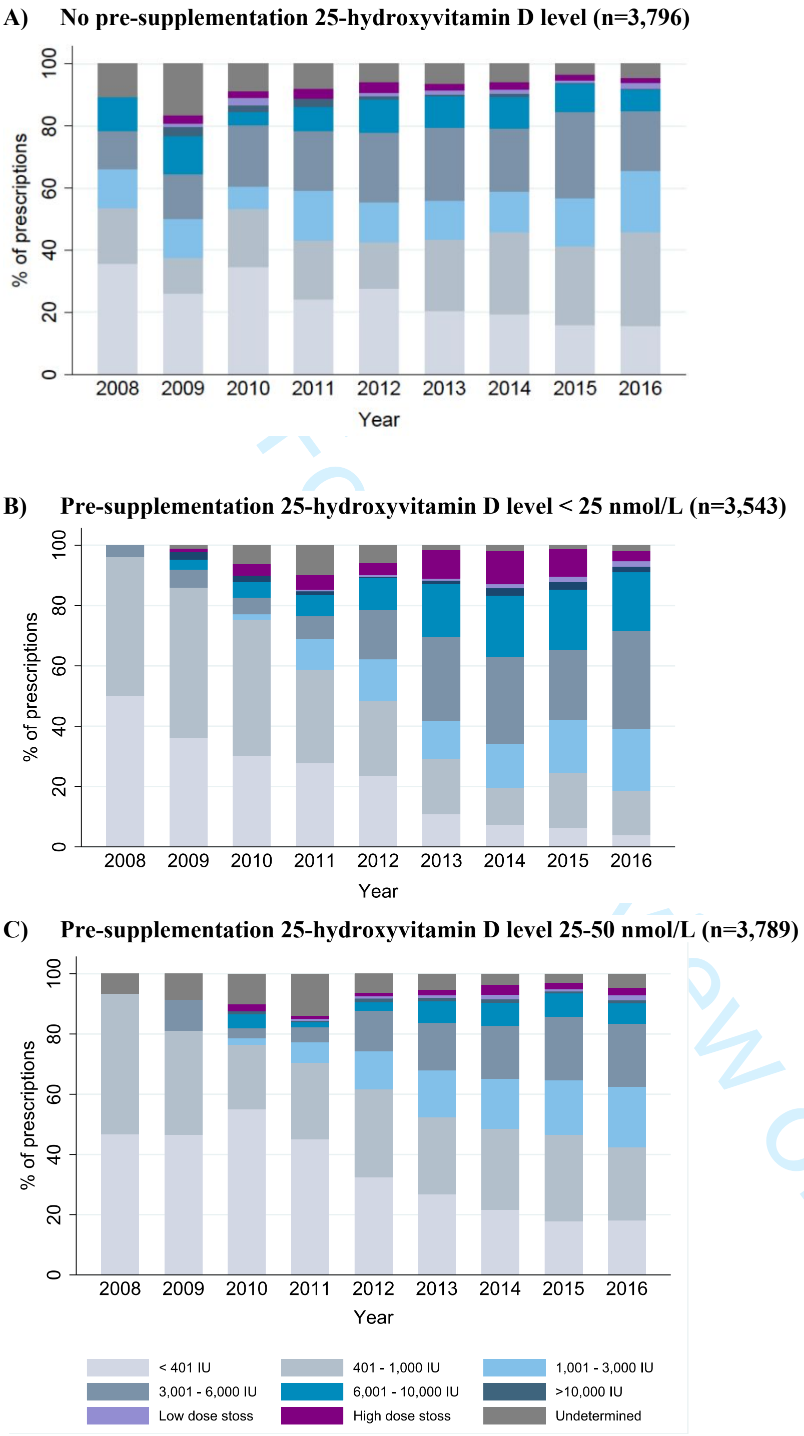

Figure 3: Dosages of incident vitamin D prescriptions presented in equivalent daily dose among children with A) no pre-supplementation 25(OH)D level, B) 25(OH)D level less than 25 nmol/L, and C) 25(OH)D level between 25 - 50 nmol/L in the 90 days prior to their incident prescriptions.

Supplementary Table S1

Drug code related to single-ingredient vitamin D products

Supplementary Table S2

Read codes related to vitamin D deficiency and 25-hydroxyvitamin D tests

Supplementary Table S3

No. of children with a record of diagnosis Read codes suggestive of vitamin D deficiency in the 90 days prior to or on the date of the incident prescription, stratified by pre-supplementation 25-hydroxyvitamin D testing status

Supplementary Figure S1

Time trends in vitamin D supplementation prescribing in children by product types

Supplementary Table S4

Doses of vitamin D supplementation prescribed stratified by age group

Supplementary Table S5

Vitamin D supplementation dosing frequencies observed in the study cohort

For peer review only

Supplementary Table S1

Drug code related to single-ingredient vitamin D products

1a. Drug code related to single-ingredient vitamin D products

Drug code	Description
29742978	colecalciferol 800unit capsules
30212978	colecalciferol 2,500units/drop oral drops sugar free
30582978	colecalciferol 40,000unit tablets
30643978	colecalciferol 800unit capsules
32236978	colecalciferol 4,000unit tablets
32237978	colecalciferol 4,000unit tablets
32265978	colecalciferol 4,000unit capsules
32267978	colecalciferol 1,000unit tablets
32317978	colecalciferol 50,000unit capsules
34135978	colecalciferol 3,000unit tablets
34138978	colecalciferol 800unit capsules
34144978	colecalciferol 1,000unit capsules
38846978	colecalciferol 20,000unit tablets
39354978	colecalciferol 50,000units/1ml oral solution unit dose ampoules sugar free
39355978	colecalciferol 50,000units/1ml oral solution unit dose ampoules sugar free
47464978	colecalciferol 2,740units/ml oral drops sugar free
47465978	colecalciferol 2,740units/ml oral drops sugar free
48011978	colecalciferol 15,000units/5ml oral solution
51017978	colecalciferol 15,000units/5ml oral solution
51018978	colecalciferol 15,000units/5ml oral solution
51473978	colecalciferol 200units/drop oral drops sugar free
51484978	colecalciferol 1,000unit capsules
52073979	colecalciferol 40,000unit capsules
52074979	colecalciferol 40,000unit capsules
52154979	colecalciferol 20,000unit capsules
53089979	colecalciferol 15,000units/5ml oral solution
53090979	colecalciferol 15,000units/5ml oral solution
53091979	colecalciferol 15,000units/5ml oral solution
53092979	colecalciferol 15,000units/5ml oral solution
53321979	ergocalciferol 125microgram tablets
53322979	ergocalciferol 125microgram tablets
53352979	colecalciferol 20,000unit tablets
53353979	colecalciferol 5,000unit tablets
53355979	colecalciferol 400unit tablets
53356979	colecalciferol 30,000unit capsules
53357979	colecalciferol 5,000unit capsules
53358979	colecalciferol 3,000unit capsules
53359979	ergocalciferol 12.5microgram tablets
53362979	colecalciferol 400unit capsules
53365979	colecalciferol 1,000unit capsules
53366979	colecalciferol 600unit capsules
53367979	colecalciferol 2,200unit capsules
53368979	colecalciferol 10,000unit capsules
53369979	colecalciferol 20,000unit capsules
53370979	colecalciferol 20,000unit capsules
53371979	colecalciferol 50,000unit capsules
53377978	colecalciferol 2,400units/ml oral drops sugar free
53378978	colecalciferol 2,400units/ml oral drops sugar free
53461978	ergocalciferol 12.5microgram tablets
53464978	colecalciferol 20,000unit capsules
53641979	ergocalciferol 250microgram tablets
53650979	ergocalciferol 1.25mg capsules
54637979	colecalciferol 500unit capsules
54638979	colecalciferol 500unit capsules
54716979	colecalciferol 1,000unit capsules
54790979	ergocalciferol 1.25mg capsules
54889979	colecalciferol 3,000units/ml oral solution
54890979	colecalciferol 2,000units/ml oral solution

For peer review only

1a. Drug code related to single-ingredient vitamin D products - continued

Drug code	Description
54891979	colecalciferol 2,000units/ml oral solution
54916979	colecalciferol 800unit tablets
54917979	colecalciferol 800unit tablets
55053979	colecalciferol 3,000unit tablets
55054979	colecalciferol 20,000unit tablets
55055979	colecalciferol 2,200unit tablets
55056979	colecalciferol 10,000unit tablets
55060979	colecalciferol 5,000unit tablets
55061979	ergocalciferol 3,000units/ml oral solution
55062979	ergocalciferol 3,000units/ml oral solution sugar free
55064979	colecalciferol 3,000units/ml oral solution sugar free
55065979	colecalciferol 20,000units/ml oral solution sugar free
55066979	colecalciferol 30,000unit capsules
55067979	colecalciferol 30,000unit capsules
55068979	colecalciferol 5,000unit capsules
55070979	colecalciferol 400unit capsules
55072979	colecalciferol 1,000unit capsules
55074979	colecalciferol 2,200unit capsules
55076979	colecalciferol 10,000unit capsules
55077979	colecalciferol 20,000unit capsules
55078979	colecalciferol 50,000unit capsules
55079979	colecalciferol 50,000unit capsules
55194978	colecalciferol 10,000units/ml oral solution sugar free
55195978	colecalciferol 10,000units/ml oral drops sugar free
55196978	colecalciferol 10,000units/ml oral drops sugar free
55197978	colecalciferol 10,000units/ml oral drops sugar free
55257978	colecalciferol 20,000unit capsules
55558979	ergocalciferol 50,000unit capsules
56123979	ergocalciferol 1.25mg capsules
56242979	colecalciferol 3,000unit tablets
56244979	colecalciferol 20,000unit tablets
56246979	colecalciferol 2,200unit tablets
56248979	colecalciferol 10,000unit tablets
56310979	colecalciferol 400unit tablets
56313979	colecalciferol 1,000unit tablets
56949978	colecalciferol 25,000unit tablets
56950978	colecalciferol 25,000unit tablets
57024978	colecalciferol 1,000unit tablets
57965979	colecalciferol 20,000unit capsules
58139979	colecalciferol 5,000unit tablets
58209979	ergocalciferol 3,000units/ml oral solution
58346979	colecalciferol 800unit capsules
58349979	colecalciferol 5,000unit capsules
58353979	colecalciferol 1,000unit capsules
58355979	colecalciferol 20,000unit capsules
58356979	colecalciferol 20,000unit capsules
58768979	colecalciferol 2,000units/ml oral solution sugar free
58769979	colecalciferol 2,000units/ml oral solution sugar free
58782979	colecalciferol 3,000units/ml oral solution sugar free
59522978	colecalciferol 2,000unit tablets
59525978	colecalciferol 40,000unit capsules
59527978	colecalciferol 5,000unit tablets
59528978	colecalciferol 20,000unit capsules
59769979	colecalciferol 800units/ml oral drops sugar free
59770979	colecalciferol 800units/ml oral drops sugar free
59771979	colecalciferol 400units/dose oral spray sugar free
59772979	colecalciferol 400units/dose oral spray sugar free
59843979	colecalciferol 20,000unit capsules
59981979	ergocalciferol 15,000units/5ml oral solution
60056979	ergocalciferol 300,000units/1ml solution for injection ampoules

For peer review only

1a. Drug code related to single-ingredient vitamin D products - continued

Drug code	Description
60224979	colecalciferol 300,000units/1ml solution for injection ampoules
60225979	ergocalciferol 300,000units/1ml solution for injection ampoules
60373979	fultium-d3 800unit capsules
60374979	colecalciferol 800unit capsules
60566979	vitamin d2 3,000units/ml oral solution sugar free
60568979	ergocalciferol 20,000units/ml oral solution sugar free
60572979	colecalciferol 20,000units/ml oral solution sugar free
60594979	colecalciferol 1,000unit tablets
61563979	colecalciferol 30,000units/5ml oral solution
61592979	colecalciferol 2,000units/ml oral drops sugar free
61593979	colecalciferol 2,000units/ml oral drops sugar free
61910979	colecalciferol 400unit tablets
61911979	colecalciferol 400unit tablets
62001979	colecalciferol 1,000units/ml oral solution
62002979	colecalciferol 1,000units/ml oral solution
62087979	colecalciferol 10,000units/ml oral solution
62088979	colecalciferol 10,000units/ml oral solution
62116979	colecalciferol 5,000unit capsules
62118979	colecalciferol 3,000unit capsules
62152979	colecalciferol 6,000units/5ml oral suspension
62324979	colecalciferol 20,000units/ml oral drops
62356979	ergocalciferol 12.5microgram tablets
62357979	ergocalciferol 12.5microgram tablets
62536979	colecalciferol 400unit capsules
62622979	colecalciferol 1,000unit tablets
62626979	colecalciferol 1,000unit capsules
62643979	colecalciferol 600unit capsules
62645979	colecalciferol 2,200unit capsules
62986979	colecalciferol 20,000unit capsules
63201979	colecalciferol 2,000unit capsules
64131979	colecalciferol 4,000units/5ml oral suspension
68364979	ergocalciferol 400units/5ml oral solution
68382979	ergocalciferol 20,000units/5ml oral suspension
68384979	ergocalciferol 20,000units/5ml oral solution
68392979	ergocalciferol 120units/5ml oral solution
68396979	ergocalciferol 100,000units/5ml oral solution
68780978	colecalciferol 20,000unit capsules
68781978	colecalciferol 20,000unit capsules
69915979	colecalciferol 400units/5ml oral suspension
69917979	colecalciferol 20,000units/5ml oral suspension
69920979	colecalciferol 1,000units/5ml oral suspension
70249978	ergocalciferol 1.25mg capsules
73011978	colecalciferol 25,000units/1ml oral solution unit dose ampoules sugar free
73012978	colecalciferol 25,000units/1ml oral solution unit dose ampoules sugar free
73102978	colecalciferol 3,200unit capsules
73128978	colecalciferol 10,000unit capsules
73129978	colecalciferol 20,000unit capsules
73301978	colecalciferol 20,000units/ml oral solution
73391978	colecalciferol 3,200unit capsules
73392978	colecalciferol 3,200unit capsules
77093978	colecalciferol 15,000units/5ml oral solution
78387978	colecalciferol 500unit orodispersible tablets sugar free
78388978	colecalciferol 500unit orodispersible tablets sugar free
78389978	colecalciferol 2,000unit orodispersible tablets sugar free
78390978	colecalciferol 2,000unit orodispersible tablets sugar free
78426978	colecalciferol 2,200unit tablets
78428978	colecalciferol 5,000unit tablets
78441978	colecalciferol 1,000unit capsules
78719978	colecalciferol 2,000unit tablets
78720978	colecalciferol 2,000unit tablets

For peer review only

1a. Drug code related to single-ingredient vitamin D products - continued

Drug code	Description
79321978	colecalciferol 200units/drop oral drops sugar free
79322978	colecalciferol 200units/drop oral drops sugar free
79338978	ergocalciferol 6,000units/5ml oral solution
79340978	ergocalciferol 10,000units/5ml oral solution
79935978	ergocalciferol 600,000units/1.5ml solution for injection ampoules
79960978	colecalciferol 280unit chewable tablets
79961978	colecalciferol 280unit chewable tablets
79993978	colecalciferol 20,000unit tablets
80016978	colecalciferol 400unit capsules
80018978	colecalciferol 1,000unit tablets
80019978	colecalciferol 1,000unit capsules
80022978	colecalciferol 20,000unit capsules
80147979	ergocalciferol 800units/5ml oral suspension
80151979	ergocalciferol 6,000units/5ml oral suspension
80153979	ergocalciferol 50,000units/5ml oral suspension
80157979	ergocalciferol 30,000units/5ml oral suspension
80159979	ergocalciferol 3,000units/5ml oral suspension
80169979	ergocalciferol 10,000units/5ml oral suspension
80416979	colecalciferol 15,000units/5ml oral suspension
80883998	colecalciferol 3,000units/ml oral solution sugar free
80885998	colecalciferol 3,000units/ml oral solution sugar free
80886998	colecalciferol 20,000units/ml oral solution sugar free
80887998	colecalciferol 20,000units/ml oral solution sugar free
80914998	colecalciferol 2,000units/ml oral drops sugar free
80915998	colecalciferol 2,000units/ml oral drops sugar free
80947998	ergocalciferol 20,000units/ml oral solution sugar free
80952998	colecalciferol 800unit capsules
80958998	colecalciferol 20,000unit capsules
81004998	colecalciferol 30,000unit capsules
81007998	colecalciferol 30,000unit capsules
81009998	colecalciferol 10,000unit capsules
81010998	colecalciferol 10,000unit capsules
81011998	colecalciferol 2,500unit capsules
81012998	colecalciferol 2,500unit capsules
81013998	colecalciferol 1,000unit capsules
81014998	colecalciferol 1,000unit capsules
81015998	colecalciferol 400unit capsules
81074998	colecalciferol 5,000unit capsules
81138998	colecalciferol 500 units tablets
81252998	colecalciferol 1,000unit tablets
81253998	colecalciferol 1,000unit tablets
81328998	colecalciferol 800unit capsules
81329998	colecalciferol 800unit capsules
81335998	colecalciferol 20,000unit capsules
81464998	colecalciferol 400unit capsules
81568998	colecalciferol 10000units/ml oral solution
81612998	colecalciferol 1,000unit tablets
81624998	colecalciferol 10,000units/5ml oral suspension
81631998	colecalciferol 2,200unit capsules
81710998	colecalciferol 50,000unit capsules
82283998	colecalciferol 15,000units/5ml oral solution
82513998	colecalciferol 20,000unit capsules
82514998	ergocalciferol 1.25mg capsules
82946978	colecalciferol 20,000unit tablets
82956978	colecalciferol 20,000unit capsules
83392978	colecalciferol 50,000unit tablets
83393978	colecalciferol 50,000unit tablets
83468978	colecalciferol 20,000unit tablets
83469978	colecalciferol 10,000unit tablets
83480978	colecalciferol 800unit capsules

For peer review only

1a. Drug code related to single-ingredient vitamin D products - continued

Drug code	Description
83743978	ergocalciferol 1,000units/5ml oral solution
83744978	colecalciferol 5,000units/5ml oral solution
83746978	colecalciferol 5,000units/5ml oral solution
83747978	colecalciferol 10,000units/5ml oral solution
83748978	colecalciferol 10,000units/5ml oral solution
83750978	colecalciferol 10,000units/5ml oral solution
84072978	colecalciferol 5,000unit tablets
84414978	ergocalciferol 1,500units/ml oral solution sugar free
84586978	colecalciferol 20,000unit tablets
84592978	colecalciferol 400unit tablets
84600978	colecalciferol 1,000unit tablets
84680998	colecalciferol oral liquid
85005998	ergocalciferol oral liquid
85220998	ergocalciferol 600,000units/2ml solution for injection ampoules
85224998	ergocalciferol 300,000units/1ml solution for injection ampoules
85329978	colecalciferol 1,000unit tablets
86614979	ergocalciferol 300,000units/1ml solution for injection ampoules
86615979	ergocalciferol 300,000units/1ml solution for injection ampoules
93163990	ergocalciferol 600,000units/2ml solution for injection ampoules
93164990	ergocalciferol 300,000units/1ml solution for injection ampoules
93384992	calciferol 10 mcg tab
93390992	calciferol 1,000 iu i/u tab
93401992	calciferol 5000 i/u tab
94809990	ergocalciferol 1.25mg tablets
94810990	ergocalciferol 250microgram tablets
95606992	ergocalciferol 600ku/1.5ml solution
95872992	calciferol 3,000 iu tab
95873992	calciferol 30,000 iu/ml inj
95875992	calciferol soln 400u/5ml in arachis oil sol
95877992	calciferol 40,000 iu/ml inj
95878992	calciferol 400 iu/ml sol
96131992	calciferol 400 iu/ml i/u syr
96132992	calciferol .25 mg cap
96133992	calciferol 1 mg cap
96134992	calciferol 1,000 iu i/u cap
96599992	calciferol 75 mcg inj
96600992	ergocalciferol 250microgram tablets
96601992	calciferol 20,000 iu cap
96788998	ergocalciferol 400000units/ml oral solution
97023992	calciferol 100,000 iu/ml inj
97024992	calciferol 600,000 iu/ml inj
97032992	calciferol 300 iu/ml i/u syr
97033992	calciferol drops liq
97034992	calciferol 400 i/u inj
97035992	calciferol 9,000 iu/ml liq
97036992	calciferol 1,000 iu/5ml sol
97037992	calciferol 300 mg tab
97038992	calciferol 15 mcg tab
97238989	ergocalciferol 1.25mg tablets
97238990	ergocalciferol 250microgram tablets
97767998	calciferol bp 3000units/ml solution
98374992	vitamin d2 tab
98421997	ergocalciferol 1.25mg tablets
98421998	ergocalciferol 250microgram tablets
98422997	ergocalciferol 1,000units/5ml oral suspension
98422998	ergocalciferol 3,000units/ml oral solution sugar free
98925998	ergocalciferol 600,000units/2ml solution for injection ampoules
99762990	ergocalciferol 250microgram tablets
99763988	ergocalciferol 250microgram tablets
99763990	ergocalciferol 300,000units/1ml solution for injection ampoules

For peer review only

1b. Drug code related to combination vitamin D products

Drug code	Description
34026978	colecalciferol 1,000unit / calcium carbonate 2.5g chewable tablets
34027978	colecalciferol 1,000unit / calcium carbonate 2.5g chewable tablets
52015979	colecalciferol 800unit / calcium carbonate 1.25g chewable tablets
52016979	colecalciferol 800unit / calcium carbonate 1.25g tablets
56947978	colecalciferol 800unit / calcium carbonate 2.5g chewable tablets
56948978	colecalciferol 800unit / calcium carbonate 2.5g chewable tablets
58270979	generic caldrink d3 liquid
60543979	colecalciferol 100unit / calcium carbonate 400mg chewable tablets
60544979	colecalciferol 100unit / calcium carbonate 400mg chewable tablets
61594979	colecalciferol 150unit / calcium 250mg tablets
61595979	colecalciferol 150unit / calcium 250mg tablets
64223979	calcium carbonate 400mg / ergocalciferol 2.5microgram tablets
64224979	calcium carbonate 400mg / ergocalciferol 2.5microgram tablets
64830979	calcium and ergocalciferol tablets
64831979	colecalciferol 100unit / calcium carbonate 400mg tablets
68756978	colecalciferol 880unit / calcium carbonate 2.5g chewable tablets
68757978	colecalciferol 880unit / calcium carbonate 2.5g chewable tablets
73294978	generic healthy start children's vitamin drops
73598978	colecalciferol 100unit / calcium carbonate 400mg tablets
80849998	calcium carbonate 400mg with colecalciferol 100 units chewable tablets
81191998	colecalciferol 400unit / calcium carbonate 1.5g tablets
81268998	colecalciferol 200unit / calcium carbonate 750mg tablets
81269998	colecalciferol 200unit / calcium carbonate 750mg tablets
81655998	colecalciferol 800unit / calcium carbonate 1.25g chewable tablets
81656998	colecalciferol 800unit / calcium carbonate 1.25g chewable tablets
82214998	colecalciferol 400unit / calcium carbonate 1.25g tablets
82215998	colecalciferol 400unit / calcium carbonate 1.25g chewable tablets
82229978	generic littlevit multivitamin drops
82344998	colecalciferol 400unit / calcium carbonate 1.5g tablets
82429979	colecalciferol 400unit / calcium carbonate 1.25g chewable tablets
82483998	colecalciferol 400unit / calcium carbonate 1.5g chewable tablets
82546978	colecalciferol 400unit / calcium carbonate 1.5g chewable tablets
82752998	generic sandocal+d 1200 effervescent tablets
82753998	calcium lactate gluconate with calcium carbonate and colecalciferol 1200mg calcium with 800iu effervescent tablets
82966998	generic sandocal+d 600 effervescent tablets
82967998	calcium lactate gluconate with calcium carbonate and colecalciferol 600mg calcium with 400iu effervescent tablets
83420998	colecalciferol 400unit / calcium carbonate 1.25g chewable tablets
83638978	colecalciferol 1,000unit / folic acid 400microgram capsules
83639978	colecalciferol 1,000unit / folic acid 400microgram capsules
84212998	risedronate sodium 35mg with calcium carbonate 2500mg & colecalciferol 22micrograms tablets and granules
84286998	colecalciferol 400unit / calcium carbonate 1.5g effervescent tablets
84287998	colecalciferol 400unit / calcium carbonate 1.5g chewable tablets
84292998	colecalciferol 400unit / calcium carbonate 1.5g chewable tablets
84362998	vitamins c & d3 with folic acid tablets
84363998	vitamins c & d3 with folic acid tablets
86598998	alendronic acid 70mg / colecalciferol 70microgram tablets
86599998	alendronic acid 70mg / colecalciferol 70microgram tablets
88002998	dolomite with vitamin d tablets
88144997	calcium carbonate 600mg with vitamin d % micrograms with minerals chewable tablets
88144998	calcium carbonate 600mg with vitamin d and minerals 5 micrograms tablets
88225998	calcium carbonate 600mg with vitamin d and 5 micrograms tablets
88233979	generic abidec multivitamin drops
89183998	colecalciferol 400unit / calcium carbonate 1.25g chewable tablets
89439998	colecalciferol 440unit / calcium carbonate 1.25g effervescent granules sachets
89637998	colecalciferol 400unit / calcium carbonate 1.5g chewable tablets
89647998	colecalciferol 400unit / calcium carbonate 1.5g chewable tablets
89828997	multivitamin and mineral chewable tablets

1b. Drug code related to combination vitamin D products - continued

Drug code	Description
89828998	calcium carbonate 600mg with vitamin d and minerals 5 micrograms tablets
90498998	nicotinic acid with colecalciferol and calcium gluconate tablet
91053998	colecalciferol 800unit / calcium phosphate 3.1g oral powder sachets
91526998	calcium carbonate 600mg with vitamin d and 5 micrograms tablets
91987979	colecalciferol 400unit / calcium carbonate 1.5g chewable tablets
91989979	colecalciferol 400unit / calcium carbonate 1.25g chewable tablets
91990979	colecalciferol 400unit / calcium carbonate 1.25g chewable tablets
91991979	colecalciferol 400unit / calcium carbonate 1.25g chewable tablets
91993979	colecalciferol 400unit / calcium carbonate 1.25g chewable tablets
91994979	colecalciferol 400unit / calcium carbonate 1.25g chewable tablets
91995979	colecalciferol 200unit / calcium carbonate 1.25g chewable tablets
91996979	colecalciferol 400unit / calcium carbonate 1.25g chewable tablets
91997979	colecalciferol 400unit / calcium carbonate 1.25g chewable tablets
91998979	colecalciferol 200unit / calcium carbonate 1.25g chewable tablets
92000979	colecalciferol 200unit / calcium carbonate 1.25g chewable tablets
92001979	colecalciferol 200unit / calcium carbonate 1.25g chewable tablets
92004979	calcium and ergocalciferol tablets
93127992	calcium 500mgs & vitamin d 400iu tab
93276998	generic abidec multivitamin drops
94021990	calcium and ergocalciferol tablets
94089992	calcium + vitamin d 300 mg tab
94756992	calcium & vitamin d 6000 i/u tab
96015992	vit.a /vit.b1 /vit.b2 /vit.c /vit.d2 /ni pow
96020992	calcium and ergocalciferol tablets
96737998	calciferol with calcium tablets
96789998	calcium and ergocalciferol tablets
96897998	calcium and ergocalciferol tablets
96920998	colecalciferol 800unit / calcium phosphate 3.1g oral powder sachets
96959996	colecalciferol 440unit / calcium carbonate 1.25g effervescent granules sachets
96959997	colecalciferol 400unit / calcium carbonate 1.25g chewable tablets
96959998	colecalciferol 200unit / calcium carbonate 1.25g chewable tablets
97031992	calciferol/calcium gluconate tab
97064992	calcium 500mgs & vitamin d 200iu i/u tab
97138998	calcium and ergocalciferol tablets
97248990	calcium and ergocalciferol tablets
97780990	calcium and ergocalciferol tablets
97815990	calcium and ergocalciferol tablets
97935998	generic abidec multivitamin drops
98247992	vit.a /vit.b1 /vit.b2 /vit.c /vit.d2 /ni eli
98249990	calcium and ergocalciferol tablets
98581990	calcium and ergocalciferol tablets
98700997	colecalciferol 200unit / calcium carbonate 1.25g chewable tablets
98700998	colecalciferol 200unit / calcium carbonate 1.25g chewable tablets
99018990	calcium and ergocalciferol tablets
99733992	ascorbic acid/vitamin a/vitamin d 2 25 mg tab

For peer review only

Supplementary Table S2

Read codes related to vitamin D deficiency and 25-hydroxyvitamin D tests

Read code	Description
C28..00	vitamin d deficiency
C28..11	osteomalacia
C28..12	rickets
C280.00	active rickets
C282.00	osteomalacia unspecified
C28z.00	avitaminosis d nos
C2B..00	vitamin d insufficiency
J690.11	coeliac rickets
K080300	renal rickets
44LA.00	serum vitamin d
44LM.00	plasma 25-hydroxyvitamin d3 level
44LP.00	serum 25-hydroxy vitamin d3 level
44Lg.00	serum vitamin d2 level
44Ln.00	serum 25-hydroxyvitamin d2 level
4QB4.00	vitamin d level
4QB4000	vitamin d2 level
4QB4100	vitamin d3 level
4QB4200	25-hydroxyvitamin d2 level
4QB4300	25-hydroxyvitamin d3 level
4QB4400	combined total vitamin d2 and d3 level
4QB4500	25-hydroxyvitamin d level
4QB4600	total 25-hydroxyvitamin d level
4QB4700	serum total 25-hydroxy vitamin d level
5788	radiobioassay - vitamin d

For peer review only

Supplementary Table S3

No. of children with a record of diagnosis Read codes suggestive of vitamin D deficiency in the 90 days prior to or on the date of the incident prescription, stratified by pre-supplementation 25-hydroxyvitamin D testing status

	25-hydroxyvitamin D testing at initiation of supplementation (ng/mL)							
	**<10** (n=3,738)	**10 - 20** (n=3,898)	**>20 - 30** (n=441)	**>30 - 50** (n=31)	**>50** (n=9)	**No test** (n=3,986)	**Undetermined** (n=174)	**Total** (n=12,277)
**No. of children with symptoms, n**								
Musculoskeletal and non-specific pain	750	769	67	4	0	340	23	1,953
Tiredness and fatigue	556	547	46	3	0	106	5	1,263
Failure to thrive	25	33	7	1	0	36	3	105
Bone fracture	13	15	2	1	0	42	6	79
Skeletal deformity	15	26	8	1	0	35	2	87
Numbness or paraesthesia	14	9	1	0	0	7	0	31
Hypocalcaemia	9	2	2	0	0	16	0	29
Seizure or tetany	1	3	0	0	0	16	1	21
Abnormal gait	4	9	1	0	0	6	1	21
Muscle weakness	9	7	2	0	0	3	0	21
Delay in motor development	0	0	0	0	0	5	0	5
Cardiomyopathy	0	0	0	0	0	2	0	2
**Total, n (%)**	1,396 (37.3)	1,420 (36.4)	136 (30.8)	10 (32.3)	0 (0)	614 (15.4)	41 (23.6)	3,617 (29.5)

SI conversion factors: To convert 25-hydroxyvitamin D to nmol/L, multiply values by 2.496.

For peer review only

Supplementary Figure S1

Time trends in vitamin D supplementation prescribing in children by product types (n=11,743)

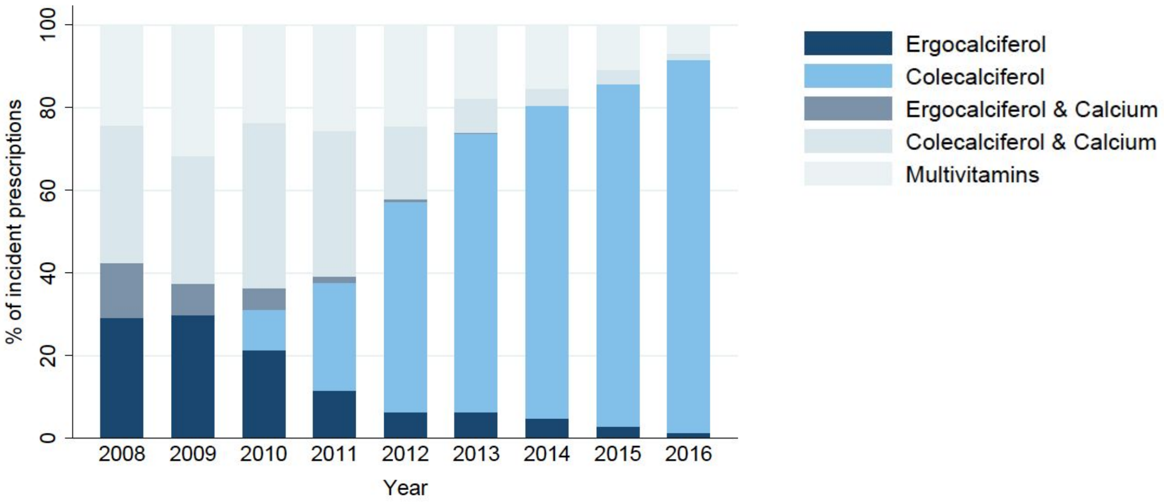

peer review only

Supplementary Table S4

Doses of vitamin D supplementation prescribed stratified by age group

	**Age group**			
	**0 - 1 year (n = 453)**	**2 - 5 years (n = 1,665)**	**6 - 11 years (n = 3,085)**	**12 - 17 years (n = 6,540)**
Equivalent daily dose, n (%)	392 (86.5%)	1,460 (87.7%)	2,826 (91.6%)	5,928 (90.6%)
≤400 IU/day	193	690	942	640
401 – 1,000 IU/day	37	193	574	2,012
1,001 – 3,000 IU/day	65	162	395	1,073
3,001 – 6,000 IU/day	88	385	774	1,081
6,001 – 10,000 IU/day	8	26	120	1,016
>10,000 IU/day	1	4	21	106
Stoss therapy, n (%)	5 (1.1%)	29 (1.7%)	84 (2.7%)	437 (6.7%)
<150,000 IU	2	9	31	86
150,000 – 300,000 IU	3	20	52	290
>300,000 IU	0		1	61
Undetermined, n (%)	56 (12.3%)	176 (10.6%)	175 (5.7%)	174 (2.7%)

For peer review only

For peer review only - http://bmjopen.bmj.com/site/about/guidelines.xhtml

Supplementary Table S5

Vitamin D supplementation dosing frequencies observed in the study cohort (n=11,743)

Dosing Frequencies	n (%)
Once daily	7,376 (62.8)
Once every 7 days	1,337 (11.4)
Twice a day	1,012 (8.6)
Undetermined	581(5)
Stoss	555 (4.7)
Once 2 times a week	267 (2.3)
Once every 14 days	227 (1.9)
Once 3 times a week	157 (1.3)
Once every 2 days	93 (0.8)
Once every 28 days	41 (0.4)
3 times a day	35 (0.3)
Once every 42 days	19 (0.2)
Others	43 (0.4)

For peer review only

For peer review only - http://bmjopen.bmj.com/site/about/guidelines.xhtml

The RECORD statement – checklist of items, extended from the STROBE statement, that should be reported in observational studies using routinely collected health data.

	Item No.	STROBE items	Location in manuscript where items are reported	RECORD items	Location in manuscript where items are reported
**Title and abstract**					
	1	(a) Indicate the study's design with a commonly used term in the title or the abstract (b) Provide in the abstract an informative and balanced summary of what was done and what was found		RECORD 1.1: The type of data used should be specified in the title or abstract. When possible, the name of the databases used should be included.<br><br>RECORD 1.2: If applicable, the geographic region and timeframe within which the study took place should be reported in the title or abstract.<br><br>RECORD 1.3: If linkage between databases was conducted for the study, this should be clearly stated in the title or abstract.	1,2<br><br>1,2<br><br>N/A
**Introduction**					
Background rationale	2	Explain the scientific background and rationale for the investigation being reported			4,5
Objectives	3	State specific objectives, including any prespecified hypotheses			4,5
**Methods**					
Study Design	4	Present key elements of study design early in the paper			5,6
Setting	5	Describe the setting, locations, and relevant dates, including periods of recruitment, exposure, follow-up, and data collection			5,6

For peer review only

Participants	6	*(a) Cohort study* - Give the eligibility criteria, and the sources and methods of selection of participants. Describe methods of follow-up<br>*Case-control study* - Give the eligibility criteria, and the sources and methods of case ascertainment and control selection. Give the rationale for the choice of cases and controls<br>*Cross-sectional study* - Give the eligibility criteria, and the sources and methods of selection of participants<br><br>*(b) Cohort study* - For matched studies, give matching criteria and number of exposed and unexposed<br>*Case-control study* - For matched studies, give matching criteria and the number of controls per case		RECORD 6.1: The methods of study population selection (such as codes or algorithms used to identify subjects) should be listed in detail. If this is not possible, an explanation should be provided.<br><br>RECORD 6.2: Any validation studies of the codes or algorithms used to select the population should be referenced. If validation was conducted for this study and not published elsewhere, detailed methods and results should be provided.<br><br>RECORD 6.3: If the study involved linkage of databases, consider use of a flow diagram or other graphical display to demonstrate the data linkage process, including the number of individuals with linked data at each stage.	6, Supplementary<br><br>N/A<br><br>N/A
Variables	7	Clearly define all outcomes, exposures, predictors, potential confounders, and effect modifiers. Give diagnostic criteria, if applicable.		RECORD 7.1: A complete list of codes and algorithms used to classify exposures, outcomes, confounders, and effect modifiers should be provided. If these cannot be reported, an explanation should be provided.	6<br>Supplementary
Data sources/ measurement	8	For each variable of interest, give sources of data and details of methods of assessment (measurement).<br>Describe comparability of assessment methods if there is more than one group			6,7

For peer review only

Bias	9	Describe any efforts to address potential sources of bias			6,7,8
Study size	10	Explain how the study size was arrived at			5,6
Quantitative variables	11	Explain how quantitative variables were handled in the analyses. If applicable, describe which groupings were chosen, and why			6,7,8
Statistical methods	12	(a) Describe all statistical methods, including those used to control for confounding<br>(b) Describe any methods used to examine subgroups and interactions<br>(c) Explain how missing data were addressed<br>(d) *Cohort study* - If applicable, explain how loss to follow-up was addressed<br>*Case-control study* - If applicable, explain how matching of cases and controls was addressed<br>*Cross-sectional study* - If applicable, describe analytical methods taking account of sampling strategy<br>(e) Describe any sensitivity analyses			7,8
Data access and cleaning methods		..		RECORD 12.1: Authors should describe the extent to which the investigators had access to the database population used to create the study population.	16

For peer review only

				RECORD 12.2: Authors should provide information on the data cleaning methods used in the study.	
Linkage		..		RECORD 12.3: State whether the study included person-level, institutional-level, or other data linkage across two or more databases. The methods of linkage and methods of linkage quality evaluation should be provided.	N/A
**Results**					
Participants	13	(a) Report the numbers of individuals at each stage of the study (*e.g.*, numbers potentially eligible, examined for eligibility, confirmed eligible, included in the study, completing follow-up, and analysed)<br>(b) Give reasons for non-participation at each stage.<br>(c) Consider use of a flow diagram		RECORD 13.1: Describe in detail the selection of the persons included in the study (*i.e.,* study population selection) including filtering based on data quality, data availability and linkage. The selection of included persons can be described in the text and/or by means of the study flow diagram.	5,6
Descriptive data	14	(a) Give characteristics of study participants (*e.g.*, demographic, clinical, social) and information on exposures and potential confounders<br>(b) Indicate the number of participants with missing data for each variable of interest<br>(c) *Cohort study* - summarise follow-up time (*e.g.*, average and total amount)			9<br>Table 1
Outcome data	15	*Cohort study* - Report numbers of outcome events or summary measures over time<br>*Case-control study* - Report numbers in each exposure			9,10,11,12<br>Table 2<br>Figure 1<br>Figure 2<br>Figure 3

For peer review only

		category, or summary measures of exposure<br>*Cross-sectional study* - Report numbers of outcome events or summary measures			
Main results	16	(a) Give unadjusted estimates and, if applicable, confounder-adjusted estimates and their precision (e.g., 95% confidence interval). Make clear which confounders were adjusted for and why they were included<br>(b) Report category boundaries when continuous variables were categorized<br>(c) If relevant, consider translating estimates of relative risk into absolute risk for a meaningful time period			9,10,11,12
Other analyses	17	Report other analyses done—e.g., analyses of subgroups and interactions, and sensitivity analyses			9,10,11,12
**Discussion**					
Key results	18	Summarise key results with reference to study objectives			12
Limitations	19	Discuss limitations of the study, taking into account sources of potential bias or imprecision. Discuss both direction and magnitude of any potential bias		RECORD 19.1: Discuss the implications of using data that were not created or collected to answer the specific research question(s). Include discussion of misclassification bias, unmeasured confounding, missing data, and changing eligibility over time, as they pertain to the study being reported.	13,14
Interpretation	20	Give a cautious overall interpretation of results considering objectives,			12,13

For peer review only

		limitations, multiplicity of analyses, results from similar studies, and other relevant evidence			
Generalisability	21	Discuss the generalisability (external validity) of the study results			15
**Other Information**					
Funding	22	Give the source of funding and the role of the funders for the present study and, if applicable, for the original study on which the present article is based			16
Accessibility of protocol, raw data, and programming code		..		RECORD 22.1: Authors should provide information on how to access any supplemental information such as the study protocol, raw data, or programming code.	16

*Reference: Benchimol EI, Smeeth L, Guttmann A, Harron K, Moher D, Petersen I, Sørensen HT, von Elm E, Langan SM, the RECORD Working Committee. The REporting of studies Conducted using Observational Routinely-collected health Data (RECORD) Statement. *PLoS Medicine* 2015; in press.

*Checklist is protected under Creative Commons Attribution (CC BY) license.

BMJ Open

Vitamin D prescribing in children in UK primary care practices: a population-based cohort study

Journal:	*BMJ Open*
Manuscript ID	bmjopen-2019-031870.R1
Article Type:	Original research
Date Submitted by the Author:	20-Aug-2019
Complete List of Authors:	Wan, Mandy; Guy's and Saint Thomas' NHS Foundation Trust, Evelina Pharmacy; King's College London, Institute of Pharmaceutical Science<br>Horsfall, Laura; University College London, Research Department of Primary Care and Population Health<br>Basatemur, Emre; University College London, Population, Policy and Practice Programme<br>Patel, Jignesh; King's College London, Institute of Pharmaceutical Science<br>Shroff, Rukshana; Great Ormond Street Hospital For Children NHS Trust,<br>Rait, Greta; University College London, Research Department of Primary Care and Population Health
**Primary Subject Heading**:	Paediatrics
Secondary Subject Heading:	Epidemiology
Keywords:	PAEDIATRICS, Paediatric endocrinology < PAEDIATRICS, EPIDEMIOLOGY

SCHOLARONE™
Manuscripts

Vitamin D prescribing in children in UK primary care practices: a population-based cohort study

Mandy Wan (ORCID ID: 0000-0001-8802-0425), Laura J Horsfall (ORCID ID: 0000-0002-0868-5660), Emre Basatemur, Jignesh P Patel (ORCID ID: 0000-0003-4197-8294), Rukshana Shroff* (ORCID ID: 0000-0001-8501-1072), and Greta Rait* (ORCID ID: 0000-0002-7216-7294)

*These authors share senior authorship

Mandy Wan: Lead Paediatric Research Pharmacist at Guy's and St Thomas' NHS Foundation Trust, Evelina London Children's Hospital, Pharmacy Department, London, UK; and PhD student at Institute of Pharmaceutical Science, King's College London, London, UK.

Laura Horsfall: Senior Epidemiologist and Wellcome Trust Fellow at Research Department of Primary Care and Population Health, University of College London, London, UK.

Emre Basatemur: Clinical Research Fellow at Population, Policy and Practice Programme, University of College London Institute of Child Health, London, UK.

Jignesh P Patel: Reader and Honorary Consultant Pharmacist in Anticoagulation at Institute of Pharmaceutical Science, King's College London, London, UK and Department of Haematological Medicine, King's College Hospital Foundation NHS Trust, London, UK.

Rukshana Shroff: Consultant Paediatric Nephrologist at Renal Unit, Great Ormond Street Hospital NHS Foundation Trust, London, UK.

Greta Rait: Professor of Primary Care and Health Services Research at Research Department of Primary Care and Population Health, University of College London, London, UK.

Corresponding author: Mandy Wan
Email: mandy.wan@gstt.nhs.uk
Organisation: Guy's and St Thomas' NHS Foundation Trust
Evelina London Children's Hospital
Pharmacy Department
Westminster Bridge Road
London
SE1 7EH

For peer review only - http://bmjopen.bmj.com/site/about/guidelines.xhtml

Abstract

Objective To examine temporal changes in the incidence and patterns of vitamin D supplementation prescribing by general practitioners (GPs) between 2008 and 2016.

Design Population-based cohort study.

Setting UK general practice health records from The Health Improvement Network (THIN).

Participants Children aged 0 to 17 years who were registered with their general practices for at least 3 months.

Outcome measures Annual incidence rates of vitamin D prescriptions were calculated, and rate ratios were estimated using multivariable Poisson regression to explore differences by sociodemographic factors. Data on the type of supplementation, dose, dosing schedule, linked 25-hydroxyvitamin D [25(OH)D] laboratory test results, and clinical symptoms suggestive of vitamin D deficiency were analysed.

Results Among 2 million children, the crude annual incidence of vitamin D prescribing increased by 26-fold between 2008 and 2016 rising from 10.8 (95% CI: 8.9, 13.1) to 276.8 (95% CI: 264.3, 289.9) per 100,000 person-years. Older children, non-white ethnicity, and general practices in England (compared to Wales/Scotland/Norther Ireland) were independently associated with higher rates of prescribing. Analyses of incident prescriptions showed inconsistent supplementation regimens with an absence of recorded pre-supplementation 25(OH)D concentrations in 28.7 to 56.4% of prescriptions annually. There was an increasing trend in prescribing at pharmacological doses irrespective of 25(OH)D concentrations, deviating in part from UK recommendations. Prescribing at pharmacological doses for children with deficient status increased from 3.8 to 79.4%, but the rise was also observed in children for whom guidelines recommended prevention doses (0 to 53%). Vitamin D supplementation at pharmacological doses was also prescribed to at least 40% of children with no pre-supplementation 25(OH)D concentrations.

Conclusions There has been a marked and sustained increase in vitamin D supplementation prescribing to children in UK primary care. Our data suggests that national guidelines on vitamin D supplementation for children are not consistently followed by GPs.

For peer review only - http://bmjopen.bmj.com/site/about/guidelines.xhtml

Strengths and limitations of this study

- This is the first population-based study to examine the extent and variability of vitamin D supplementation prescribing practice using representative primary care data.
- This study does not contain data from secondary care, however, vitamin D deficiency is largely managed in the primary care setting.
- Although several explanations for the factors that might have contributed to changes in vitamin D supplementation prescribing over time are provided, it was not possible to determine the practitioners' rationale for prescribing, nor could we assess clinical appropriateness of prescriptions on an individual level.

For peer review only

For peer review only - http://bmjopen.bmj.com/site/about/guidelines.xhtml

Introduction

Vitamin D is an essential prohormone that humans obtain through cutaneous photosynthesis, diet, or supplementation. While few would argue the importance of its physiological role in maintaining calcium homeostasis and bone mineralisation, the beneficial effects of vitamin D supplementation is a topic of much controversy.[1–5] In children, severe vitamin D deficiency can cause rickets and hypocalcaemic seizures, but the clinical consequences of vitamin D insufficiency are less established despite expanding literature in this area. Findings from numerous epidemiological studies have linked low 25-hydroxyvitamin D [25(OH)D] concentrations to an increased risk of a myriad of adverse health consequences in both adults and children[6,7], where low concentrations in children have been linked to asthma[8,9], eczema[10], respiratory tract infections[11,12], and diabetes[13], among others.[6] Yet, the majority of randomised controlled trials do not show improved outcomes with vitamin D supplementation,[6,7,14,15] and meta-analyses are inconclusive and widely debated.[6–8,11,12,14–18]

Against this backdrop of ongoing debate, vitamin D deficiency is widely prevalent in all age groups and seen as a growing public health concern worldwide.[19] The US,[20] Canada,[21] Australia,[22,23] France,[24] and the UK[25,26] have all reported a significant increase in laboratory vitamin D testing, with as much as a 90-fold increase over a 10-year period.[22,23] In England, there has also been a marked increase in the number of vitamin D prescriptions issued by general practitioners (GPs) in primary care, with annual spending on vitamin D prescriptions increasing from £0.5 to £40 million between 2007 and 2016[27]; a rising trend that was partly attributed to vitamin D prescriptions issued for children.[28]

These reported trends have understandably led many to question the clinical appropriateness of vitamin D testing in practice.[29,30] However, even though vitamin D is also widely available without a prescription, the increasing number of GPs issued vitamin D prescriptions has surprisingly not attracted the same degree of attention. For the UK where the healthcare service is publicly-funded and free, national strategy is centred on primary prevention in managing vitamin D deficiency. Specifically, UK national guidance (A summary of UK guidelines is presented in Supplementary Table S1) has, until relatively recently, targeted recommendations on supplementation mainly towards high-risk groups.[31–

For peer review only - http://bmjopen.bmj.com/site/about/guidelines.xhtml

[40] All children under 5 years of age including breastfed infants, if their mother is also at risk of vitamin D deficiency, were recommended to take daily vitamin D supplement at prevention doses.[32–35,37,38] There were no specific recommendations for children 5 years or above unless individuals were considered at risk. In any children where 25(OH)D is tested, treatment at pharmacological doses is guided by whether 25(OH)D concentration is below 25 nmol/L, the threshold set by the UK Scientific Advisory Committee on Nutrition as vitamin D deficient.[31,39] Little is known about how well these recommendations are implemented in practice, but it has been noted that the complexity of the advice and poor communications may have caused confusion among both health professionals and the public.[37] Considering there are numerous other international and national guidelines on vitamin D supplementation and health which differ in their definition of vitamin D deficiency and supplementation recommendations[41–43], a comprehensive analysis of vitamin D prescribing by GPs offers an opportunity to facilitate the appropriate use of vitamin D supplementation for patient benefit and contain healthcare expenditure.

The aim of our study was to examine recent trends in the prescribing pattern of vitamin D supplementation in children. We quantified temporal changes in the incidence of vitamin D prescriptions issued by GPs in a UK population-based study. Moreover, we examined the proportions of prescriptions by type of supplementation, dose, dosing schedule, as well as the proportions that can be linked to 25(OH)D laboratory test results and clinical symptoms suggestive vitamin D deficiency.

Methods

Data source

We obtained electronic health records from patients registered with UK general practices contributing to The Health Improvement Network (THIN). The THIN database contains anonymised data from 744 general practices using the Vision computer system (In Practice Systems, London, UK); data are from all patients in participating practices unless individual patients opt-out of THIN. It contains data of approximately 16 million patients which has been shown to be broadly representative of the UK

For peer review only - http://bmjopen.bmj.com/site/about/guidelines.xhtml

population in terms of age, sex, prevalence of medical conditions, and mortality rates.[44] As of 2015, the THIN dataset is reported to cover 6% of the UK population.[45]

THIN contains information on diagnoses, laboratory tests, symptoms and hospital referrals, as well as basic sociodemographic information. Clinical events and diagnoses are recorded using a hierarchical coding system called Read codes.[46] THIN also includes records of all prescriptions issued and these are linked to the British National Formulary. Prescribing data are particularly well recorded in THIN since the computerised entry made by the GP is directly used to issue a prescription to the patient. Prescribing data in THIN has been shown to be comparable to data on dispensed prescriptions with a mean practice redemption rate for all prescribing of 98.5%.[47]

Study population

Study cohort selection is presented in supplementary Figure S1. We identified a cohort of children aged 0 to 17 years registered with a THIN general practice from 1st January 2008 to 31st December 2016. Individuals were not eligible to enter the study until three months after their registration with the practice in order to exclude those with prevalent prescriptions. Cohort entry was defined by the latest of: the study start date (1st January 2008), 3 months after the patient's registration with the practice, or the date the practice met two predefined quality indicators (acceptable mortality recording and acceptable computer usage) for electronic data recording.[49,50] Children with cystic fibrosis, chronic renal failure, inflammatory bowel diseases or chronic liver diseases were excluded as these conditions are known to affect vitamin D absorption or metabolism and therefore require specialist management (Code lists for these conditions were created using previously published methodology[51]). Children were followed until an incident prescription of vitamin D, or censored on death, midpoint of their 18th birthday, end of registration with a practice, the last collection date of data from the practice, or end of the study period (31st December 2016), whichever occurred first. The total number of patient-years between cohort entry and exit defined the denominator for incidence calculations.

Outcome

For peer review only - http://bmjopen.bmj.com/site/about/guidelines.xhtml

A vitamin D prescription was defined as a first prescription record (incident prescription) of either: 1) a single-ingredient product containing calciferol, ergocalciferol or colecalciferol as the only active ingredient; or 2) a combination product containing calciferol, ergocalciferol or colecalciferol with other active ingredients (e.g. ergocalciferol with calcium, multi-vitamin preparations). For the latter, only those that could be linked to a Read code indicating vitamin D deficiency or insufficiency or 25(OH)D testing within 90 days either side of the date of the prescription were included. Comprehensive lists of drug codes and Read codes were developed using previously published methodology (Supplementary Table S2 and S3).[51] Incident cases were defined as children with a first ever recording of a vitamin D prescription in THIN during the study period. The date at which the first recording of vitamin D prescription was made was classified as the index date. Consultations where general practitioners did not issue a prescription but recommended self-purchase of over the counter vitamin D supplement were not considered as prescriptions.

Covariates

Age groups were defined as: up to 6 months, 6 months – 4 years, 5 -11, and 12-17 years. Ethnicity was grouped into the 2011 UK Census 5- category classification; children with multiple ethnicity records belonging to different categories were included in the analysis under the 'missing data' category. Townsend index, an area-based relative measure of material deprivation, was extracted; the value closest to the start of the observation period was used for patients with multiple records. For this exploratory study, a pragmatic approach was taken to handle missing data separately under the 'missing data' category.

25(OH)D laboratory test results associated with incident prescriptions were identified by temporal proximity. A result was considered to be contemporary and would have formed the basis of prescribing if it occurred 90 days prior to the index date. Based on the elimination half-life of 25(OH)D, results recorded between 91-180 days after the index date were used to assess vitamin D status post supplementation. Concentrations <25 nmol/L were categorised as deficient as per the UK Department of Health and Social Care definition relevant at the time of the study period.[31] Concentrations were

For peer review only - http://bmjopen.bmj.com/site/about/guidelines.xhtml

further categorised as insufficient (25-50 nmol/L) as defined by the British Paediatric and Adolescent Bone Group and supported by the British Society of Paediatric Radiology and the Royal College of Paediatrics and Child Health.[34,36,38]

Read codes of symptoms or clinical complications that could potentially be related to vitamin D deficiency within the 90 days prior to the index date were extracted and organised into 12 categories as presented in Supplementary Table S4.

Incident vitamin D prescriptions analyses

Children with multiple vitamin D prescriptions on the index date (534 patients) were excluded from prescription pattern analyses as it was not possible to determine whether all prescriptions were relevant. Prescriptions were categorised according to the type of supplementation as per case definition above. Equivalent daily dose (EDD) was calculated for each incident prescription as determined by the quantity of vitamin D [international unit (IU)] prescribed per dose and the prescribed dosing frequency. Prescriptions with titration dosages (e.g. two tablets daily for 2 weeks and then one tablet monthly) were handled by considering the first dosage only to capture the initial management strategy. Where unit of dosing was missing (e.g. every day), one single dosing unit was assumed for tablet/capsule products only. Generic (e.g. as directed), or ambiguous (e.g. once daily for a liquid product) dosages were categorised under the 'undetermined' category. EDDs were further categorised into dose bands. To assist interpretation, doses of 280 - 400 IU were considered prevention doses across all age groups based on recommendations relevant at the time of the study period.[31,33–35,39] Doses between 401-1000 IU were categorised as a separate prevention dose category given divergent views exist within the medical community with regards to supplementation strategies for primary prevention.[36–38] Doses between 1,001 and 10,000 IU/day were considered as pharmacological treatment doses independent of age.[36,38] Prescription quantity supplied in days was calculated based on total prescription quantity issued and corresponding dosage and capped at 365 days to take account of product shelf life. High-dose vitamin D regimens (stoss regimens) prescribed either as intramuscular injections or oral preparations were categorised separately and reported as either low dose (total dose <150,000 IU) or high dose (total

For peer review only - http://bmjopen.bmj.com/site/about/guidelines.xhtml

dose ≥150,000 IU) stoss. As oral stoss regimens may be prescribed as a single dose or spread over several days, prescriptions with explicit duration directions for ≤15 days (e.g. 5,000 IU once daily for 14 days), or where the quantity prescribed were ≤7 days with an equivalent daily dose ≥10,000 IU (e.g. 5 capsules of 20,000 IU with dosing direction "one capsules once daily") were also categorised as stoss.

Statistical analysis

Cohort characteristics are presented by sex, ethnicity, socioeconomic quintile, country, calendar year, and year of cohort entry as frequencies (%). Medians and interquartile range (IQR) are presented for age at entry and follow-up time. We calculated crude annual incidence rates by dividing the number of children with a first ever recording of a vitamin D prescription by the Person Years at Risk (PYAR). Confidence intervals (95% CI) were calculated assuming a Poisson distribution. Using Poisson regression, we estimated sex stratified incidence rate ratios with 95% confidence intervals, adjusting for potential confounders. Time period was fitted as both a linear and categorical variable and models compared using the log likelihood ratio test; there was evidence that the categorised time period was a better fit with the data and thus the time period was treated as a categorical variable. A fully adjusted sex stratified model with an interaction between age and ethnicity was fitted and examined; while the interaction term was statistically significant, it did not change the estimates of the other variables and the incidence rates predicted by the two models were similar. However, as the model with the interaction term resulted in unstable predictions, the interaction term was not included in the final model. The final model with the inclusion of the GP practice as a random effect to account for any data clustering was used to calculate adjusted incidence rate ratios (IRRs). Proportions of prescriptions by type of supplementation, dose, dosing schedule, linked to 25(OH)D laboratory test concentrations and clinical symptoms suggestive vitamin D deficiency, are presented as frequencies (%). Trends in prescribing characteristics were quantified using Spearman correlation coefficients using all years between 2008 and 2016. Data analysis was conducted using Stata 15 (Stata Corp, College Station, Texas).

Three sensitivity analyses were performed. Firstly, we considered a narrower case definition for vitamin D prescription, where only single-ingredient products were included. Secondly, we repeated the

For peer review only - http://bmjopen.bmj.com/site/about/guidelines.xhtml

incidence analysis using complete cases only, that is among children who had complete data on sex, age, ethnicity, socioeconomic status, country and calendar year. Finally, we considered a broader definition for prevention doses, where equivalent daily doses of ≤1,000 IU were included.

Patient and public involvement

Patient and parent/carer representatives were involved in developing study plans and approving the lay summaries for the grant supporting this study. Patients were not involved in setting the specific research question, the outcome measure, the design, or implementation of this study. No patients were asked to advise on interpretation or writing up of results. It is not possible to disseminate the results of the research to study participants, but views from patient and parent/carer representatives will be sought in disseminating the research findings.

Ethics

The THIN data collection scheme to obtain and provide anonymised patient data was approved by the National Health Service South-East Multicentre Research Ethics Committee (REC Reference: 04/MRE01/9) in 2002. The study protocol was approved by an independent Scientific Review Committee (Reference: 16THIN035).

Results

A total of 2,051,403 eligible children from 723 general practices contributed data between Jan 1, 2008 and Dec 31, 2016, giving a total of 8 million person-years of follow-up. Of those children, 12,277 had an incident vitamin D prescription during the study period. Patient characteristics stratified by sex, ethnicity, country of general practice, and socioeconomic status are shown in the Table 1 for the overall cohort and the incident cohort.

Crude incidence

The crude annual incidence of vitamin D prescribing in children increased by 26-fold between 2008 and 2016 rising from 10.8 (95% CI: 8.9, 13.1) to 276.8 (95% CI: 264.3, 289.9) per 100,000 person-

For peer review only - http://bmjopen.bmj.com/site/about/guidelines.xhtml

years. Figure 1A shows temporal trends in vitamin D prescribing in children alongside key national guidelines or reports on vitamin D deficiency.[31–40,52] Prescribing rates for females were higher than males, with an average ratio of male to female prescribing rate of 1:1.5 across all time periods. As can be seen from Figure 1B, increasing prescribing trend (rate per 100,000 person-years) was particularly apparent in children aged 12 - 17 years [14.8 (95% CI: 11.1, 19.8) to 529.5 (95% CI: 499.2, 561.7)], followed by those aged 5 - 11 years [7.8 (95% CI: 5.4, 11.2) to 188.3 (95% CIL 172.5, 205.5)], 6 months - 4 years [10.2 (95% CI: 6.9, 15.1) to 111.6 (95% CI: 96.8, 128.7)], and less than 6 months. [10.6 (95% CI: 1.5, 75.2) to 79 (95% CI: 32.9, 189.7)]. When vitamin D prescription was defined using single-ingredient vitamin D products only, we found similar trends to the main analysis (Figure 1A).

Adjusted incidence rates

In multivariable analysis, increasing age, non-white ethnicity, and social deprivation were associated with higher incidence rates of vitamin D prescribing (Table 2). Among children aged 12-17 years, sex difference in incidence rate ratios was observed, with 1.8-fold higher vitamin D supplementation prescribing in girls than boys, respectively. General practices in England were also associated with higher incidence rate ratios as compared to Wales, Scotland and Northern Ireland. For both males and females, adjusted incidence rate ratios were higher than their corresponding crude values, with a 32-fold and 39-fold increase in the incidence of vitamin D prescribing between 2008 and 2016, respectively. A sensitivity analysis using complete cases only was comparable to the main analysis, albeit the magnitude of the effects of ethnicity was marginally greater for both sexes, along with a smaller temporal difference in annual incidence rates (Supplementary Table S5).

25-hydroxyvitamin D testing and presence of symptoms at initiation of supplementation

25(OH)D concentrations recorded before starting vitamin D supplementation are presented in Figure 2A. Overall, 28.7 to 56.4% of prescriptions annually had no linked 25(OH)D results recorded in the 3 months prior to the index date. The proportion of children with laboratory confirmed vitamin D deficiency [25(OH)D <25 nmol/L] increased from 25.7 to 40.9% between 2008 and 2011 before showing a gradual decrease to 27.2% in 2016, coinciding with the issue of public health guidelines on

For peer review only - http://bmjopen.bmj.com/site/about/guidelines.xhtml

primary prevention of vitamin D deficiency for at risk individuals. Over the study period, increased prescribing of vitamin D supplementation was observed in children with insufficient status [25(OH)D between 25-50 nmol/L], increasing from 15.8 - 21.7% between 2008 and 2011 to 27.9 - 37.1% during the period between 2012 to 2016.

29.5% of children had symptoms related to vitamin D deficiency (Supplementary Table S5). The most commonly recorded symptoms were those related to musculoskeletal or non-specific pain, tiredness or fatigue. In the group of children with no recorded pre-supplementation 25(OH)D results, only 15.4% had recorded symptoms.

Types of supplementation

Overall, 109 different pharmaceutical preparations of at least 32 dosage strengths (e.g. 3,000 IU/mL, 20,000 IU/tablet) were prescribed. A temporal change in supplementation form was observed; colecalciferol products accounted for 9.9% of the prescriptions in 2010 and increased to 90.2% in 2016, explaining the trends in prescribing patterns shown in Figure 1 (Supplementary Figure S2).

Doses and frequencies of administration

The EDD prescribed ranged from 27 to 160,000 IU/day (prescribed doses by age group is summarised in Supplementary Table S6) with a median duration of supply of 56 days (IQR: 30, 83 days). There were 237 (2.0%) prescriptions with doses less than the lowest recommended primary prevention dose (<280 IU/day), and 132 (1.1%) prescriptions with doses above the highest recommended pharmacological dose (>10,000 IU/day). A total of 24 different dosing frequencies were noted, with 37.2% of children on regimens other than once daily dosing (Supplementary Table S7).

Trend analysis of supplementation regimens stratified by pre-supplementation 25(OH)D concentrations is presented in Figure 3. Among children with deficiency [25(OH)D <25 nmol/L], prescriptions with EDD >1,000 IU/day, considered as pharmacological doses, showed a yearly increased from 3.8% in 2008 to 79.4% in 2016 (spearman's rho = 0.983, p<0.001), which is consistent with UK

For peer review only

recommendations. However, increased used of pharmacological doses (0% in 2008 to 53% in 2016; spearman's rho = 1, p<0.001) was also noted among children with 25(OH)D concentrations between 25-50 nmol/L who, according to UK national guidance, should have been recommended supplement at prevention doses; the corresponding temporal decrease of prescriptions at prevention doses was noted irrespective of the cut-off value used to define prevention doses (400 or ≤1,000 IU). A smaller but increased trend was similarly noted in the group of children with no linked 25(OH)D concentrations (35.7% in 2008 to 49.6% in 2016; spearman's rho = 0.7333, p = 0.025). Overall, vitamin D supplementation at pharmacological doses (>1,000 IU/day or stoss) was prescribed to 42.8% of children with insufficient status, outside of UK recommendations.

25-hydroxyvitamin D testing at follow-up

In the first 90 days post initiation of vitamin D supplementation, 9% (n=1,109) of children had 25(OH)D concentrations recorded, of which 24.9% were recorded within 1 month after the index date, outside of guideline recommendations. Post-supplementation testing at the recommended time intervals was low; 25(OH)D results in the period between 91 to 180 days after incident prescriptions were available for only 8.4% (n=1,035) of children (Figure 2B). Over the study period, the proportion of children with post-supplementation 25(OH)D concentrations >50 nmol/L increased from 11.1% to 76.3%.

Discussion

In a large cohort representative of children in UK primary care, there has been a marked temporal increase in vitamin D prescribing, even after adjustment for changes in population demographics. The adjusted incidence rates increased by 32-fold and 39-fold between 2008 and 2016 for males and females, respectively. 25(OH)D concentrations for guiding supplementation were not available in over 30% of children annually, and an increasing trend in prescribing vitamin D supplementation at pharmacological doses irrespective of 25(OH)D concentrations was noted with wide variations in supplementation regimens prescribed, deviating in part from UK recommendations. Furthermore, with no clear evidence to support the use of vitamin D supplementation on health outcomes, other than for

For peer review only - http://bmjopen.bmj.com/site/about/guidelines.xhtml

the prevention or treatment of rickets and osteomalacia, this aberrant prescribing practice incurs a huge, potentially avoidable, expense to our health care system.

This is the first study to report UK estimates of incidence rates of vitamin D prescribing in children using a comprehensive case definition for vitamin D prescription. An earlier study based in England included only 156 general practices and reported a marked increase in the diagnosis of vitamin D deficiency and prescription costs of single-ingredient vitamin D products in children between 2000 and 2014.[28,53] Our study reports similar trends but provides data from a much larger number of practices across the whole of the UK, with detailed socio-demographic prescribing patterns and extending the data to 2016.

Vitamin D prescribing in children increased sharply between 2011 and 2014, coinciding with the publication of guidelines and reports from the UK government and national guideline committees that emphasise the importance of vitamin D supplementation in certain groups of vulnerable children and young people.[31–40] These have, in part, raised awareness of vitamin D deficiency among primary care practitioners, and explain the increase in vitamin D prescribing practices. Notably, the increase prescribing rates over time can be attributable primarily to children 5 years or older, suggesting widening awareness of vitamin D deficiency, with risk perceptions among GPs going beyond children considered as high risk. The observed changed in the type of supplementation prescribed, from ergocalciferol and combination products to colecalciferol products, is congruent with increase product availability of the latter[27] and may potentially also relate to data suggesting colecalciferol is more efficacious at raising serum 25(OH)D concentrations than ergocalciferol.[54]

Patient age, sex and ethnicity were identified as drivers for prescribing, in keeping with the higher prevalence rates of vitamin D deficiency reported in teenage girls, and non-white ethnic groups in the UK.[39] Interestingly, we also observed increased rates of prescribing among practices in England as compared to those in Wales (IRR = 0.3), Scotland (IRR = 0.2) and Northern Ireland (IRR = 0.2), suggesting other contextual factors influencing practitioners' prescribing behaviour; disparities may be

For peer review only - http://bmjopen.bmj.com/site/about/guidelines.xhtml

due to variations in regional strategies in implementing national guidelines, or differences in practitioners' experience and attitudes towards vitamin D deficiency, and require further exploration.[55–57]

The initiation of vitamin D supplementation should be guided by 25(OH)D concentrations, except when used for primary prevention, as indicated in UK guideline recommendations.[36,38] Yet, our results show an absence of 25(OH)D results in over 30% of prescriptions annually since 2009, a practice also reported by others.[56] It is possible that a proportion of children may have 25(OH)D concentrations checked and recorded by hospital clinicians; although children with conditions known to affect vitamin D absorption and metabolism and thus likely to receive secondary input were already excluded in our analysis. We also recognised that a proportion of these prescriptions may also have been intended for primary prevention. However, applying the most conservative primary prevention dose definition of ≤1,000 IU/day, our finding that at least 40% of prescriptions exceeded 1,000/day, and together with only a small number of children with recorded symptoms possibly related to vitamin D deficiency, would be suggestive of aberrant prescribing.

Our analysis also found an upward trend in prescribing of vitamin D supplementation at pharmacological doses independent of 25(OH)D concentrations. To some extent, this pattern observed for children with deficiency suggests that practitioners are responsive to prescribing guidelines[36,38], at least on a population level. On the other hand, a comparable increase was also seen in the prescription of pharmacological doses of vitamin D to children with vitamin D insufficiency; this deviates from UK national recommendations, and perhaps reflects confusion arising from multiple, sometimes conflicting, recommendations. At the same time, these deviations from recommendations might suggest a somewhat less cautious attitude towards the use of higher than recommended doses of vitamin D among practitioners. Vitamin D has a wide therapeutic window and risk of toxicity is low, nevertheless the potential risk of vitamin D toxicity exists and requires further exploration given its widespread, and sometimes unregulated use.

For peer review only - http://bmjopen.bmj.com/site/about/guidelines.xhtml

The major strength of our study is the large sample size that is representative of real-life clinical practice across the UK. Prescribing details in THIN are well recorded because all prescriptions from general practice are generated via a standardised computerised system. The inclusion of combination vitamin D products in the case definition also captures real life clinical practice, but some cases could be missed if combination vitamin D product was prescribed but a diagnosis of vitamin D status not entered in the primary care record. There are some limitations to our study too. The first vitamin D prescription recorded in THIN may not be the first ever prescription issued to the patient as initial prescribing may have occurred in secondary care and continued in general practice. However, most prescriptions issued by UK hospitals are limited to 7-14 days for clinically urgent conditions only, so the analysed sample is very likely to be representative of true practice. The study design did not allow us to determine the practitioners' rationale for prescribing, nor could we assess clinical appropriateness of prescriptions on an individual level. Trends in seasonal variations in prescribing was also not explored. Despite these limitations, our study explored the major clinical indicator for vitamin D supplementation, namely 25(OH)D concentrations and presence of symptoms.

Our study highlights two key implications for practice. Firstly, diverse clinical practice highlights the need for a broader understanding of the multitude factors influencing GPs' prescribing decisions for vitamin D supplementation in order to reduce inappropriate prescribing.[55,57] Further guidelines alone, even in the absence of ambiguity, are unlikely to be effective. Secondly, vitamin D supplementation exceeding recommended doses, and arguably, prescriptions at prevention doses may not be the best use of the limited health resources. On this basis, at least 32.3% of our prescriptions would fall into this category. Using the most conservative estimate from an earlier study[28] (£1.65 million for the total direct cost of vitamin D prescriptions in children per year), and without costing for consultation time with health care professionals, dispensing or tests, we estimate an annual saving of £0.5 million.

Conclusion

There has been a marked and sustained increase in vitamin D prescribing to children in primary care in the UK. While this may reflect the success of the Department of Health and Social Care's efforts in

For peer review only - http://bmjopen.bmj.com/site/about/guidelines.xhtml

raising awareness of vitamin D deficiency, findings from our study would suggest that nationally set recommendations on vitamin D supplementation are not consistently followed by GPs, in terms of the number of patients treated, the doses used for supplementation, as well as the practice of prescribing vitamin D without appropriate testing. More recent NHS England guidance[30], that recommends prescribing pharmacological doses of vitamin D supplementation only to those diagnosed with vitamin D deficiency, has the potential to reduce unnecessary prescriptions and NHS expenditure, but its impact on prescribing practice would require further evaluation as previous guidelines have not always translated into practice.

For peer review only

For peer review only - http://bmjopen.bmj.com/site/about/guidelines.xhtml

Contributors

MW, GR, RS and JP were involved in the conception of the study and obtaining funding. MW, LH, EB, JP, RS and GR contributed to the study protocol and interpretation of data. MW had full access to the database, programming code and performed the data extraction and analysis. MW drafted the manuscript which LH, EB, JP, RS and GR contributed to and revised critically before approval of the final manuscript. MW is the guarantor. The corresponding author attests that all listed authors meet authorship criteria and that no others meeting the criteria have been omitted.

Funding

This study was funded by a Clinical Doctoral Research Fellowship grant (ICA-CDRF-2016-02-057) from the UK National Institute for Health Research (NIHR). MW is a doctoral student supported by this grant. Independent expert peer reviewers provided feedback on the grant application underpinning this study but had no further role in study design, data collection, analysis, interpretation, or drafting of the manuscript. LH is fully funded by the Wellcome Trust (209207/Z/17/Z). RS holds a Career Development Fellowship with the National Institute for Health Research. A part of the work took place in the Biomedical Research Centre at Great Ormond Street Hospital for Children NHS Foundation Trust and University College London. The views expressed are those of the authors and not necessarily those of the NHS, the NIHR, or the Department of Health and Social Care.

Competing interests

All authors have completed the ICMJE uniform disclosure form at www.icmje.org/coi_disclosure.pdf and declare: no support from any organisation for the submitted work; no financial relationships with any organisations that might have an interest in the submitted worked in the previous three years; no other relationships or activities that could appear to have influenced the submitted work

Data sharing

The General Practices in The Health Improvement Network (THIN) is run by the Department of Health and Social Care. All the data are available via an application to THIN. Data acquisition is associated with a fee. Code lists used in this study will be available on request.

For peer review only

References

1 Boucher BJ. Causal link between vitamin D deficiency and ill health still possible. *BMJ* 2014; **348**: g2923.

2 Welsh P, Sattar N. Vitamin D and chronic disease prevention. *BMJ* 2014; **348**: g2280.

3 Stokes CS, Lammert F. Vitamin D supplementation: less controversy, more guidance needed. *F1000Res* 2016; **5(F1000 Faculty Rev):2017**.

4 Bischoff-Ferrari HA, Orav EJ, Abderhalden L, *et al*. Vitamin D supplementation and musculoskeletal health. *The Lancet Diabetes & Endocrinology* 2019; **7**: 85.

5 Bolland MJ, Grey A, Avenell A. Vitamin D supplementation and musculoskeletal health – Authors' reply. *The Lancet Diabetes & Endocrinology* 2019; **7**: 88–89.

6 Theodoratou E, Tzoulaki I, Zgaga L, *et a*l. Vitamin D and multiple health outcomes: umbrella review of systematic reviews and meta-analyses of observational studies and randomised trials. *BMJ* 2014; **348**: g2035.

7 Autier P, Mullie P, Macacu A, *et al.* Effect of vitamin D supplementation on non-skeletal disorders: a systematic review of meta-analyses and randomised trials. *The Lancet Diabetes & Endocrinology* 2017; **5**: 986–1004.

8 Jolliffe DA, Greenberg L, Hooper RL, *et al.* Vitamin D supplementation to prevent asthma exacerbations: a systematic review and meta-analysis of individual participant data. *The Lancet Respiratory Medicine* 2017; **5**: 881–890.

9 Martineau AR, Cates CJ, Urashima M, *et al.* Vitamin D for the management of asthma. *Cochrane Database of Systematic Reviews* 2016.

10 Huang CM, Lara-Corrales I, Pope E. Effects of Vitamin D levels and supplementation on atopic dermatitis: A systematic review. *Pediatr Dermatol* 2018; **35**: 754–760.

11 Jolliffe DA, Griffiths CJ, Martineau AR. Vitamin D in the prevention of acute respiratory infection: Systematic review of clinical studies. *The Journal of Steroid Biochemistry and Molecular Biology* 2013; **136**: 321–329.

12 Martineau AR, Jolliffe DA, Hooper RL, *et al.* Vitamin D supplementation to prevent acute respiratory tract infections: systematic review and meta-analysis of individual participant data. *BMJ* 2017; **356**: i6583.

13 Savastio S, Cadario F, Genoni G, *et al.* Vitamin D Deficiency and Glycemic Status in Children and Adolescents with Type 1 Diabetes Mellitus. *PLOS ONE* 2016; **11**: e0162554.

14 Winzenberg TM, Powell S, Shaw KA, *et al*. Vitamin D supplementation for improving bone mineral density in children. *Cochrane Database of Systematic Reviews* 2010.

15 Bolland MJ, Grey A, Avenell A. Effects of vitamin D supplementation on musculoskeletal health: a systematic review, meta-analysis, and trial sequential analysis. *The Lancet Diabetes & Endocrinology* 2018; **6**: 847-858.

16 Bischoff-Ferrari HA, Willett WC, Wong JB, *et al*. Fracture prevention with vitamin D supplementation: a meta-analysis of randomized controlled trials. *JAMA* 2005; **293**: 2257–2264.

17 World Health Organisation. Vitamin D for prevention of respiratory tract infections. WHO. http://www.who.int/elena/titles/commentary/vitamind_pneumonia_children/en/ (accessed Feb 28 2019).

18 Martineau A. Vitamin D supplementation to prevent asthma exacerbations – Authors' reply. *The Lancet Respiratory Medicine* 2018; **6**: e26–e27.

For peer review only - http://bmjopen.bmj.com/site/about/guidelines.xhtml

19 Palacios C, Gonzalez L. Is vitamin D deficiency a major global public health problem? *J Steroid Biochem Mol Biol* 2014; **144PA**: 138–145.

20 Shahangian S, Alspach TD, Astles JR, *et al*. Trends in laboratory test volumes for Medicare Part B reimbursements, 2000-2010. *Arch Pathol Lab Med* 2014; **138**: 189–203.

21 Ontario Health Technology Advisory Committee. Clinical Utility of Vitamin D Testing. *Ont Health Technol Assess Ser* 2010; **10**: 1–93.

22 Bilinski KL, Boyages SC. The rising cost of vitamin D testing in Australia: time to establish guidelines for testing. *The Medical Journal of Australia* 2012; **197**: 90.

23 Bilinski K, Boyages S. Evidence of overtesting for vitamin D in Australia: an analysis of 4.5 years of Medicare Benefits Schedule (MBS) data. *BMJ Open* 2013; **3**: e002955.

24 Caillet P, Goyer-Joos A, Viprey M, *et al*. Increase of vitamin D assays prescriptions and associated factors: a population-based cohort study. *Scientific Reports* 2017; **7**: 10361.

25 Sattar N, Welsh P, Panarelli M, *et al*. Increasing requests for vitamin D measurement: costly, confusing, and without credibility. *The Lancet* 2012; **379**: 95–96.

26 Zhao S, Gardner K, Taylor W, *et al*. Vitamin D assessment in primary care: changing patterns of testing. *London J Prim Care (Abingdon)* 2015; **7**: 15–22.

27 NHS Digital. Prescription Cost Analysis. NHS Digital. https://digital.nhs.uk/data-and-information/publications/statistical/prescription-cost-analysis (accessed Feb 28 2019).

28 Basatemur E, Hunter R, Horsfall L, *et al*. Costs of vitamin D testing and prescribing among children in primary care. *Eur J Pediatr* 2017; **176**: 1405–1409.

29 Boyages SC. Vitamin D testing: new targeted guidelines stem the overtesting tide. *Med J Aust* 2016; **204**: 18.

30 NHS England. Conditions for which over the counter items should not routinely be prescribed in primary care: Guidance for CCGs. 2018. https://www.england.nhs.uk/wp-content/uploads/2018/03/otc-guidance-for-ccgs.pdf (accessed Feb 28 2019).

31 Scientific Advisory Committee on Nutrition. Update on Vitamin D - Position statement by the Scientific Advisory Committee on Nutrition. 2007. https://assets.publishing.service.gov.uk/government/uploads/system/uploads/attachment_data/file/339349/SACN_Update_on_Vitamin_D_2007.pdf (accessed Feb 28 2019).

32 National Institute for Health and Care Excellence. Improving the nutrition of pregnant and breastfeeding mothers and children in low-income households. (PH11). 2008. https://webarchive.nationalarchives.gov.uk/20080912094204/http://www.gserve.nice.org.uk/nicemedia/pdf/PH011guidance.pdf (accessed Feb 28 2019).

33 UK Chief Medical Officers. Vitamin D - advice on supplements for at risk groups - letter from UK Chief Medical Officers. 2012. https://www.gov.uk/government/publications/vitamin-d-advice-on-supplements-for-at-risk-groups (accessed Feb 28 2019).

34 Arundel P, Ahmed SF, Allgrove J, *et al.* British Paediatric and Adolescent Bone Group's position statement on vitamin D deficiency. *BMJ* 2012; **345**: e8182.

35 Royal College of Paediatrics and Child Health. Vitamin D: position statement. 2012. https://www.rcpch.ac.uk/resources/vitamin-d-position-statement (accessed Feb 28 2019).

36 Royal College of Paediatrics and Child Health. Guide for Vitamin D in Childhood. 2013. https://www.rcpch.ac.uk/sites/default/files/2018-03/guide_to_vitamin_d_in_childhood.pdf (accessed Feb 28 2019).

For peer review only - http://bmjopen.bmj.com/site/about/guidelines.xhtml

37 National Institute for Health and Care Excellence. Vitamin D: supplement use in specific population groups (PH56). 2014. https://www.nice.org.uk/guidance/ph56/resources/vitamin-d-supplement-use-in-specific-population-groups-pdf-1996421765317 (accessed Feb 28 2019).

38 National Osteoporosis Society. Vitamin D and Bone Health: A Practical Clinical Guideline for Management in Children and Young People. 2015. https://nos.org.uk/media/2074/vitamin-d-and-bone-health-children.pdf (accessed Feb 28 2019).

39 Scientific Advisory Committee on Nutrition. SACN vitamin D and health report. 2016.

40 National Institute for Health and Care Excellence Clinical Knowledge Summaries. Vitamin D deficiency in children. 2016. https://cks.nice.org.uk/vitamin-d-deficiency-in-children#!references (accessed Feb 28 2019).

41 Rosen CJ, Abrams SA, Aloia JF, *et al.* IOM Committee Members Respond to Endocrine Society Vitamin D Guideline. *J Clin Endocrinol Metab* 2012; **97**: 1146–1152.

42 Munns CF, Shaw N, Kiely M, *et al.* Global Consensus Recommendations on Prevention and Management of Nutritional Rickets. *J Clin Endocrinol Metab* 2016; **101**: 394–415.

43 Institute of Medicine (US) Committee to Review Dietary Reference Intakes for Vitamin D and Calcium. *Dietary Reference Intakes for Calcium and Vitamin D*. National Academies Press (US): Washington (DC), 2011.

44 Blak B, Thompson M, Dattani H, *et al*. Generalisability of The Health Improvement Network (THIN) database: demographics, chronic disease prevalence and mortality rates. *Journal of Innovation in Health Informatics* 2011; **19**: 251–255.

45 IMS Health. THIN Data Guide for Researchers. 2015.

46 Chisholm J. The Read clinical classification. *BMJ* 1990; **300**: 1092.

47 NHS Digital. Prescribing Compliance, A review of the proportion of prescriptions dispensed. 2011. https://digital.nhs.uk/data-and-information/publications/statistical/prescribing-compliance/prescribing-compliance-a-review-of-the-proportion-of-prescriptions-dispensed (accessed Feb 28 2019).

48 Lewis JD, Bilker WB, Weinstein RB, *et al*. The relationship between time since registration and measured incidence rates in the General Practice Research Database. *Pharmacoepidem Drug Safe* 2005; **14**: 443–451.

49 Maguire A, Blak BT, Thompson M. The importance of defining periods of complete mortality reporting for research using automated data from primary care. *Pharmacoepidem Drug Safe* 2009; **18**: 76–83.

50 Horsfall L, Walters K, Petersen I. Identifying periods of acceptable computer usage in primary care research databases. *Pharmacoepidemiol Drug Saf* 2013; **22**: 64–69.

51 Davé S, Petersen I. Creating medical and drug code lists to identify cases in primary care databases. *Pharmacoepidem Drug Safe* 2009; **18**: 704–707.

52 Department of Health & Food Standards Agency, Department of Health & Food Standards Agency. National Diet and Nutrition Survey. Headline results from Years 1 and 2 (combined) of the Rolling Programme (2008/2009 – 2009/10). 2011. https://www.gov.uk/government/publications/national-diet-and-nutrition-survey-headline-results-from-years-1-and-2-combined-of-the-rolling-programme-2008-9-2009-10 (accessed Feb 28 2019).

53 Basatemur E, Horsfall L, Marston L, *et al*. Trends in the Diagnosis of Vitamin D Deficiency. *Pediatrics* 2017; **139**: e20162748.

54 Tripkovic L, Lambert H, Hart K, *et al.* Comparison of vitamin D2 and vitamin D3 supplementation in raising serum 25-hydroxyvitamin D status: a systematic review and meta-analysis123. *Am J Clin Nutr* 2012; **95**: 1357–1364.

For peer review only - http://bmjopen.bmj.com/site/about/guidelines.xhtml

55 Epling JW, Mader EM, Roseamelia CA, *et al*. Emerging Practice Concerning Vitamin D in Primary Care. *Qual Health Res* 2015; **25**: 1005–1012.

56 DelGiudice NJ, Street N, Torchia RJ, *et al*. Vitamin D Prescribing Practices in Primary Care Pediatrics: Underpinnings From the Health Belief Model and Use of Web-Based Delphi Technique for Instrument Validity. *Journal of Pediatric Health Care* 2018; **32**: 536–547.

57 Rockwell M, Kraak V, Hulver M, *et al*. Clinical Management of Low Vitamin D: A Scoping Review of Physicians' Practices. *Nutrients* 2018; **10**: 493.

For peer review only

Table 1: Descriptive characteristics of the overall study cohort and incident cohort

	Overall cohort (n=2,051,403)	Incident cohort (n=12,277)
Median age at entry, years (IQR)	5.5 (0.8 – 11.5)	7.6 (3.5 – 10.5)
Follow up time, years (IQR)	3.5 (1.5 – 6.3)	4.2 (2 – 6.1)
Sex, n (%)		
Male	1,055,395 (51.5)	4,936 (40.2)
Female	996,008 (48·6)	7,341 (59·8)
Ethnicity, n (%)		
White	878,360 (42.8)	2,836 (23.1)
Asian/Asian British	76,840 (3.8)	2,928 (23.9)
Black/African/Caribbean/Black British	47,206 (2.3)	1,275 (10.4)
Other ethnic group	17,656 (0.9)	312 (2.5)
Mixed/Multiple ethnic group	28,605 (1.4)	295 (2.4)
Missing	1,002,736 (48.9)	4,631 (37.7)
Country of GP practice, n (%)		
England	1,451,923 (70.8)	10,921 (88.9)
Northern Ireland	84,171 (4.1)	177 (1.4)
Scotland	310,142 (15.1)	597 (4.9)
Wales	205,167 (10)	582 (4.7)
Townsend deprivation index quintile, n (%)		
1 (least deprived)	397,057 (19.4)	1,452 (11.8)
2	357,567 (17.4)	1,377 (11.2)
3	396,234 (19.3)	2,128 (17.3)
4	379,485 (18.5)	2,740 (22.3)
5 (most deprived)	291,502 (14.2)	2,866 (23.3)
Missing	229,558 (11.2)	1,714 (14)
**Year of cohort entry, n (%)**		
2008	1,060,098 (51.7%)	6,831 (55.6%)
2009	130,610 (6.4%)	954 (7.8%)
2010	126,825 (6.2%)	961 (7.8%)
2011	160,853 (7.8%)	1,020 (8.3%)
2012	134,390 (6.6%)	870 (7.1%)
2013	139,113 (6.8%)	757 (6.2%)
2014	119,354 (5.8%)	503 (4.1%)
2015	97,198 (4.7%)	279 (2.3%)
2016	82,962 (4.0%)	102 (0.8%)

IQR = Interquartile range; Children with no recorded data on ethnicity or Townsend index were categorised as 'missing'.

For peer review only

Table 2: Multivariable Poisson regression models of incidence of vitamin D supplementation prescribing in children stratified by sex.

	**Male**	**Female**
	**IRR[a] (95% CI)**	**IRR[a] (95% CI)**
**Age group**		
Up to 6 months	1.00 (Reference)	1.00 (Reference)
6 months - 4 years	1.93 (1.21 – 3.07)	1.28 (0.84 – 1.96)
5 - 11 years	2.47 (1.55 – 3.92)	2.25 (1.48 – 3.42)
12 - 17 years	4.15 (2.61 – 6.60)	7.41 (4.87 – 11.27)
**Ethnicity**		
White	1.00 (Reference)	1.00 (Reference)
Asian	5.05 (4.60 – 5.54)	4.46 (4.12 – 4.81)
Black	2.83 (2.53 – 3.18)	2.83 (2.58 – 3.10)
Others	2.68 (2.20 – 3.26)	2.72 (2.33 – 3.19)
Mixed	1.93 (1.59 – 2.33)	1.84 (1.57 – 2.16)
Missing	1.48 (1.37 – 1.61)	1.42 (1.33 – 1.52)
**Townsend index**		
1 (least deprived)	1.00 (Reference)	1.00 (Reference)
2	1.03 (0.92 – 1.17)	1.03 (0.94 – 1.14)
3	1.18 (1.06 – 1.33)	1.20 (1.09 – 1.32)
4	1.37 (1.22 – 1.53)	1.37 (1.25 – 1.51)
5 (most deprived)	1.41 (1.25 – 1.59)	1.44 (1.31 – 1.59)
Missing	1.38 (1.19 – 1.60)	1.43 (1.26 – 1.62)
**Country**		
England	1.00 (Reference)	1.00 (Reference)
Wales	0.34 (0.25 – 0.46)	0.33 (0.25 – 0.46)
Scotland	0.24 (0.19 – 0.32)	0.18 (0.14 – 0.23)
Northern Ireland	0.24 (0.16 – 0.37)	0.17 (0.11 – 0.27)
**Year**		
2008	1.00 (Reference)	1.00 (Reference)
2009	2.47 (1.73 – 3.51)	2.61(1.93 – 3.52)
2010	3.68 (2.63 – 5.15)	4.97 (3.75 – 6.58)
2011	6.72 (4.88 – 9.25)	8.04 (6.12 – 10.56)
2012	14.60 (10.72 – 19.90)	16.16 (12.39 – 2.08)
2013	21.10 (15.52 – 28.67)	22.82 (17.53 – 29.72)
2014	25.81 (18.99 – 35.09)	28.14 (21.60 – 36.65)
2015	29.19 (21.44 – 39.76)	33.53 (25.70 – 43.74)
2016	32.27 (23.64 – 44.05)	38.87 (29.75 – 50.80)

Abbreviations: CI, confidence interval; IRR = incidence rate ratio.
[a]Adjusted for all variables listed in the table. The multilevel models included thedal practice as a random effect.

For peer review only

Figures

Figure 1: A) Time trends in vitamin D supplementation prescribing in children using all components of the case definition together and each source of case identification independently. **B)** Time trends in vitamin D supplementation prescribing in children by different age groups. Crude incidence rates with 95% confidence intervals represented by vertical bars.

Key: SACN = Scientific Advisory Committee on Nutrition; NICE = The National Institute for Health and Care Excellence; RCPCH = Royal College of Paediatrics and Child Health; NOS = National Osteoporosis Society

1) 2007: SACN: Update on Vitamin D[31]
2) 2008: NICE: Improving the nutrition of pregnant and breastfeeding mothers and children in low-income households[32]
3) 2011: The National Diet and Nutrition Survey (NDNS) rolling programme: Data from years 1 & 2[52]
4) 2012: Department of Health: Vitamin D - advice on supplements for at risk groups[33]
5) 2012: British Paediatric and Adolescent Bone Group's position statement on vitamin D deficiency[34]
6) 2012: RCPCH: Vitamin D: Position statement[35]
7) 2013: RCPCH: Guide for Vitamin D in Childhood[36]
8) 2014: NICE: Vitamin D: increasing supplement use in at-risk groups[37]
9) 2015: NOS: Vitamin D and Bone Health[38]
10) 2016: SACN: vitamin D and health report[39]
11) 2016: NICE Clinical Knowledge Summaries: Vitamin D deficiency in children[40]

Figure 2: A) 25-hydroxyvitamin D concentrations recorded in the 90 days prior to or on the index date (n=12,277); **B)** 25-hydroxyvitamon D concentrations recorded between 91-180 days after index date (only children with known levels, n=1,035). Records with ambiguous unit of measurements were categorised as 'undetermined'.

Figure 3: Dosages of incident vitamin D prescriptions presented in equivalent daily dose among children with **A)** no pre-supplementation 25(OH)D concentrations, **B)** 25(OH)D concentrations less than 25 nmol/L, and **C)** 25(OH)D concentrations between 25 - 50 nmol/L in the 90 days prior to their incident prescriptions.

For peer review only

A)

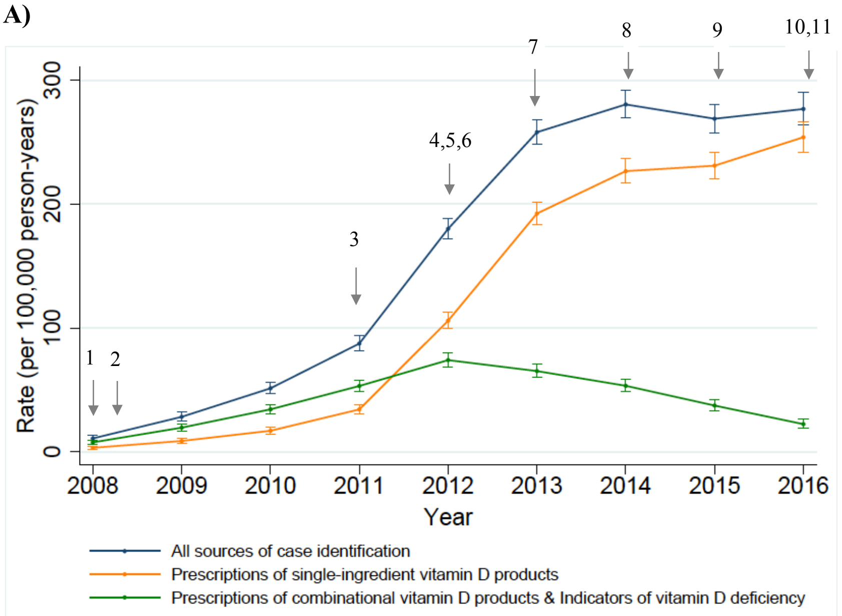

B)

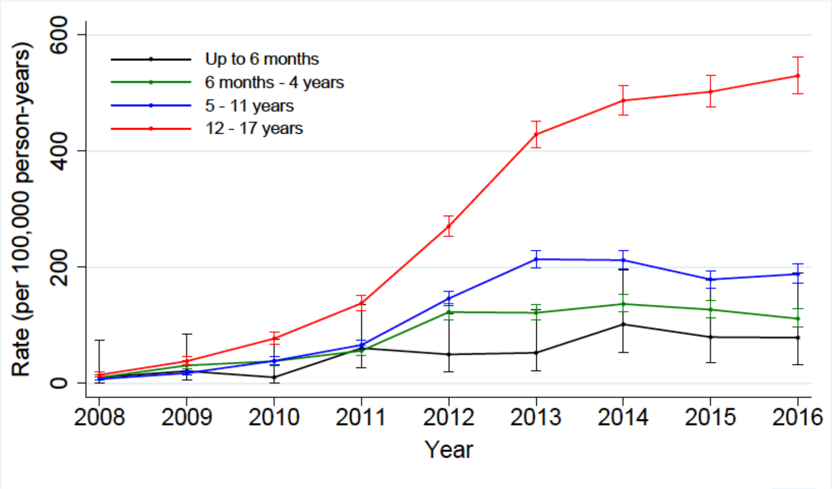

Figure 1: A) Time trends in vitamin D supplementation prescribing in children using all components of the case definition together and each source of case identification independently. **B)** Time trends in vitamin D supplementation prescribing in children by different age groups. Crude incidence rates with 95% confidence intervals represented by vertical bars.

Key: SACN = Scientific Advisory Committee on Nutrition; NICE = The National Institute for Health and Care Excellence; RCPCH = Royal College of Paediatrics and Child Health; NOS = National Osteoporosis Society

1) 2007: SACN: Update on Vitamin D[31]
2) 2008: NICE: Improving the nutrition of pregnant and breastfeeding mothers and children in low-income households[32]
3) 2011: The National Diet and Nutrition Survey (NDNS) rolling programme: Data from years 1 & 2[52]
4) 2012: Department of Health: Vitamin D - advice on supplements for at risk groups[33]
5) 2012: British Paediatric and Adolescent Bone Group's position statement on vitamin D deficiency[34]
6) 2012: RCPCH: Vitamin D: Position statement[35]
7) 2013: RCPCH: Guide for Vitamin D in Childhood[36]
8) 2014: NICE: Vitamin D: increasing supplement use in at-risk groups[37]
9) 2015: NOS: Vitamin D and Bone Health[38]
10) 2016: SACN: vitamin D and health report[39]
11) 2016: NICE Clinical Knowledge Summaries: Vitamin D deficiency in children[40]

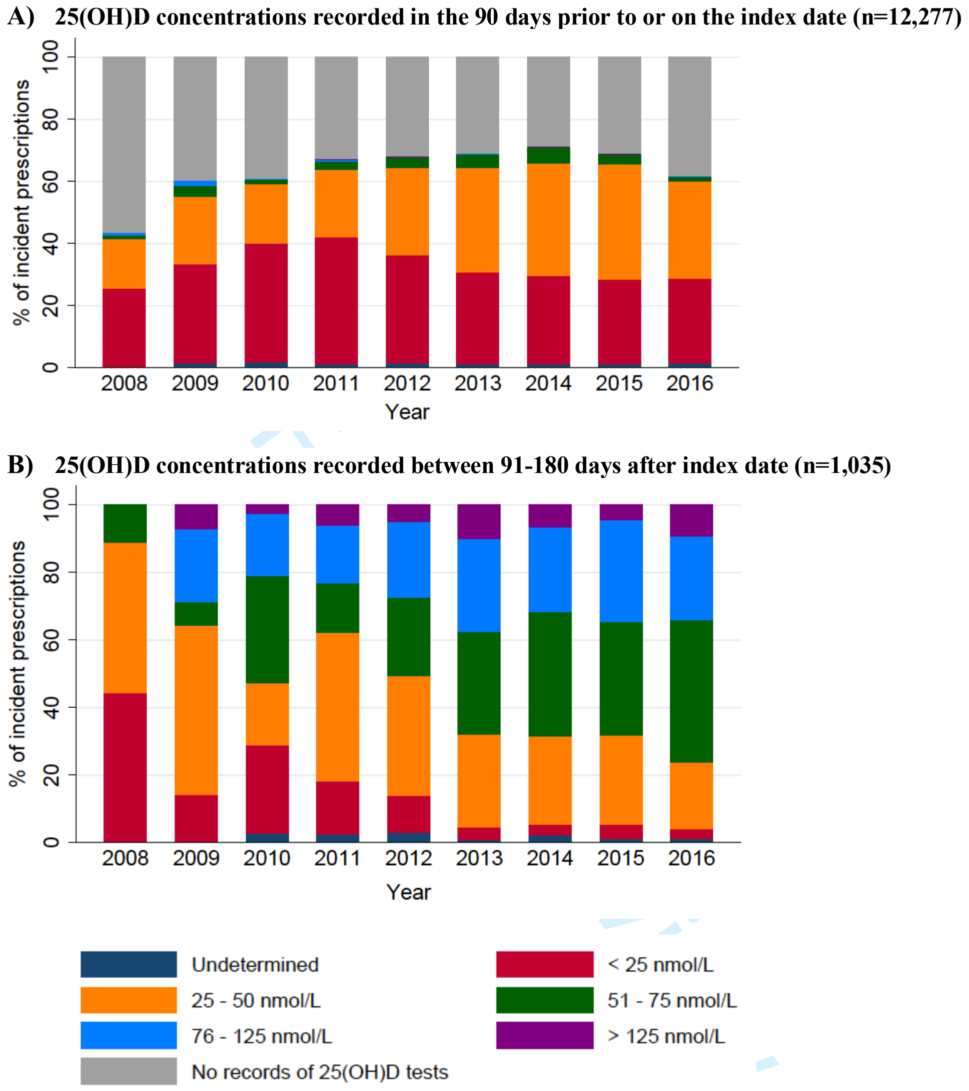

Figure 2: A) 25-hydroxyvitamin D concentrations recorded in the 90 days prior to or on the index date (n=12,277); **B)** 25-hydroxyvitamon D concentrations recorded between 91-180 days after index date (only children with known levels, n=1,035). Records with ambiguous unit of measurements were categorised as 'undetermined'.

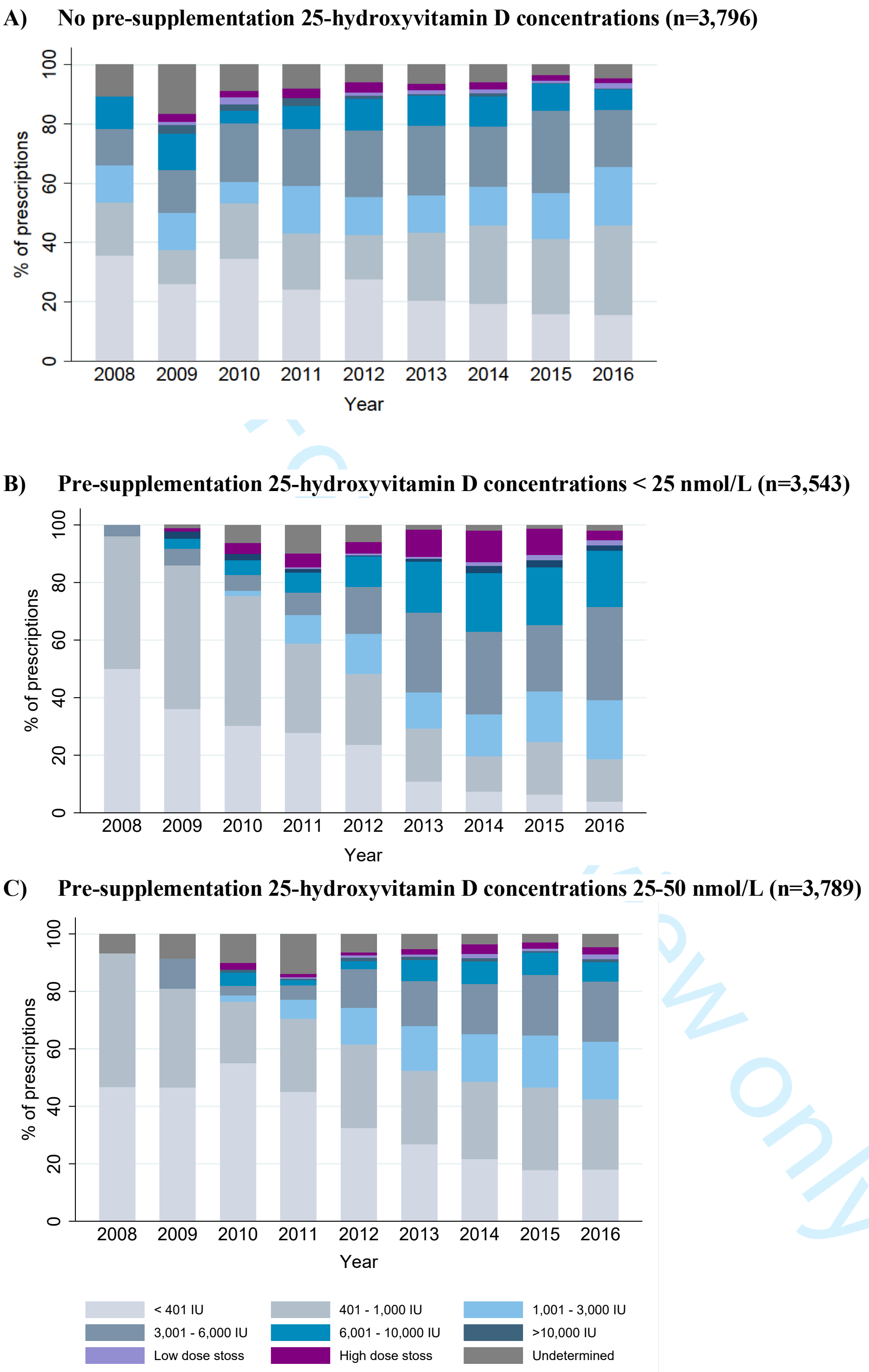

Figure 3: Dosages of incident vitamin D prescriptions presented in equivalent daily dose among children with **A)** no pre-supplementation 25(OH)D concentrations, **B)** 25(OH)D concentrations less than 25 nmol/L, and **C)** 25(OH)D concentrations between 25 - 50 nmol/L in the 90 days prior to their incident prescriptions.

Supplementary Table S1

Summary of UK guidance for the management of vitamin D deficiency in children

Supplementary Figure S1

Flowchart of study cohort selection

Supplementary Table S2

Drug code related to single-ingredient vitamin D products

Supplementary Table S3

Read codes related to vitamin D deficiency and 25-hydroxyvitamin D tests

Supplementary Table S4

No. of children with a record of diagnosis Read codes suggestive of vitamin D deficiency in the 90 days prior to or on the date of the incident prescription, stratified by pre-supplementation 25-hydroxyvitamin D concentrations

Supplementary Table S5

Multivariable Poisson regression models of incidence of vitamin D supplementation prescribing in children stratified by sex (Complete-cases only).

Supplementary Figure S2

Time trends in vitamin D supplementation prescribing in children by product types

Supplementary Table S6

Doses of vitamin D supplementation prescribed stratified by age group

Supplementary Table S7

Vitamin D supplementation dosing frequencies observed in the study cohort

For peer review only

Supplementary Table S1

Summary of UK guidance for the management of vitamin D deficiency in children

Guideline	Definition of vitamin D deficiency	Recommendations on supplementation for primary prevention	Recommendations on supplement for treatment of vitamin D deficiency
SACN (2007)	<25 nmol/L	0-6 months: 340 IU daily;<br>7 months to 3 years: 280 IU daily	No recommendations
NICE (2008)	<25 nmol/L	6 months to 4 years: 300 IU daily	No recommendations
DHSC (2012)	Not explicitly defined	6 months to 5 years: 280 - 340 IU daily<br>Those infants who are fed infant formula will not need vitamin drops until they are receiving less than 500ml of infant formula a day. Breastfed infants from one month of age if their mother has not taken vitamin D supplements throughout pregnancy.	No recommendations
BPABG (2012)	<25 nmol/L<br><br>25-50 nmol/L defined as insufficiency	6 months to 5 years: as per DHSC (2012) recommendations<br>Exclusively breastfed infants receive vitamin D supplements from soon after birth.	No recommendations
RCPCH (2012)	Not explicitly defined	As per NICE (2008) and DHSC (2012) recommendations	No recommendations
RCPCH (2013)	<25 nmol/L<br><br>25-50 nmol/L defined as insufficiency	Newborn up to 1 month: 300 - 400 IU daily<br>1 month - 18 years: 400 - 1,000 IU daily	Up to 6 months: 1,000 - 3,000 IU daily*<br>6 months - 12 years: 6,000 IU daily*<br>12 -18 years: 10,000 IU daily*<br>*For 4 - 8 weeks
NICE (2014)	<25 nmol/L	0 - 6 months: 340 IU daily<br>Older infants and children up to the age of 5: 280 IU daily	No recommendations
NOS (2015)	<25 nmol/L<br><br>25-50 nmol/L defined as insufficiency	As per DHSC (2012) recommendations	1- 6 months: 3,000 IU daily*<br>6 months - 12 years: 6,000 IU daily*<br>12 -18 years: 10,000 IU daily*<br>*For 8 – 12 weeks
SACN (2016)	<25 nmol/L	0 up to 1 year (including exclusively and partially breast-fed infants, from birth): 340 – 400 IU daily<br>1 year or older: 400 IU daily	No recommendations
NICE CKS (2016)	<25 nmol/L	As per SACN (2016) recommendations	1- 6 months: 3,000 IU daily*<br>6 months - 12 years: 6,000 IU daily*<br>12 -18 years: 10,000 IU daily*<br>*For 8 – 12 weeks

Key:

- SACN (2007): Scientific Advisory Committee on Nutrition - Update on Vitamin D[31]
- NICE (2008): The National Institute for Health and Care Excellence - Improving the nutrition of pregnant and breastfeeding mothers and children in low-income households[32]
- DHSC (2012): Department of Health and Social Care - Vitamin D - advice on supplements for at risk groups[33]
- BPABG (2012): British Paediatric and Adolescent Bone Group's position statement on vitamin D deficiency[34]
- RCPCH (2012): Royal College of Paediatrics and Child Health - Vitamin D: Position statement[35]
- RCPCH (2013): Royal College of Paediatrics and Child Health - Guide for Vitamin D in Childhood[36]
- NICE (2014): The National Institute for Health and Care Excellence - Vitamin D: increasing supplement use in at-risk groups[37]
- NOS (2015): National Osteoporosis Society - Vitamin D and Bone Health[38]
- SACN (2016): Scientific Advisory Committee on Nutrition - vitamin D and health report[39]
- NICE CKS (2016): The National Institute for Health and Care Excellence - Clinical Knowledge Summaries - Vitamin D deficiency in children[40]

For peer review only - http://bmjopen.bmj.com/site/about/guidelines.xhtml

Supplementary Figure S1

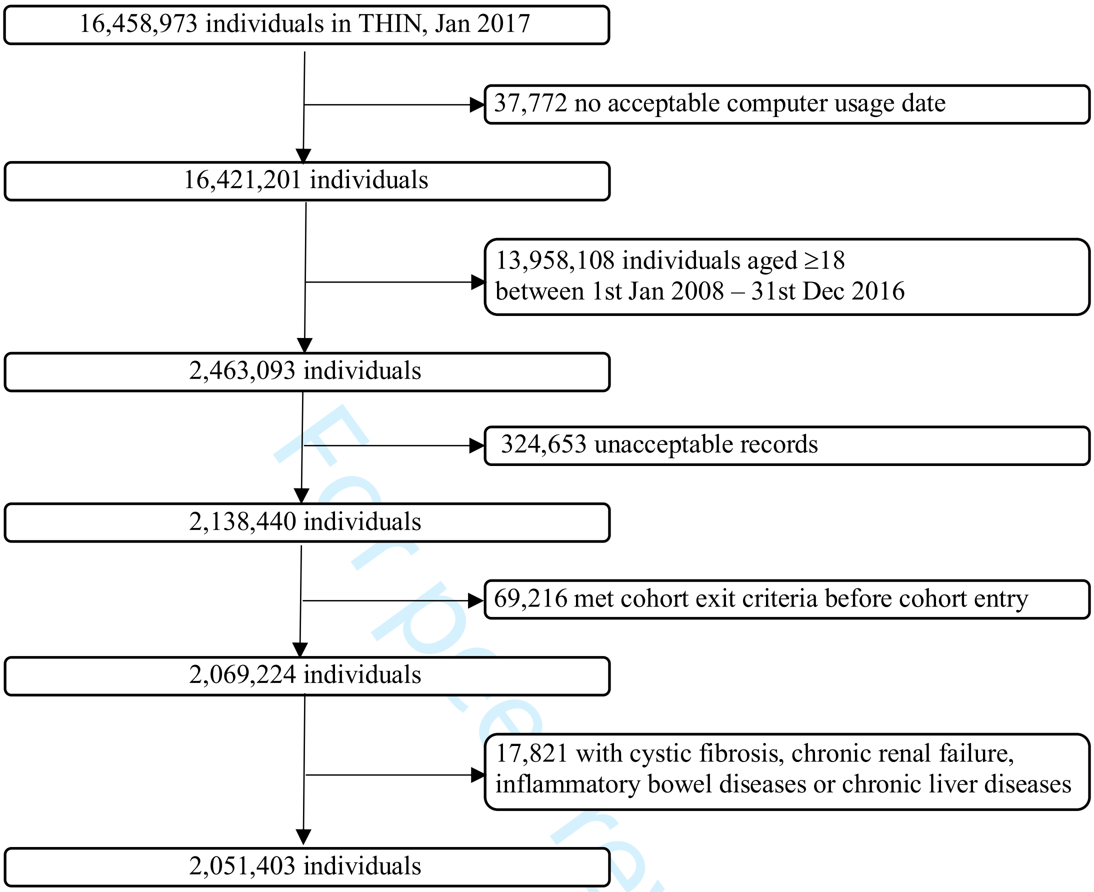

Figure S1: Flowchart of study cohort selection. The diagram displays the numbers of patients excluded from the THIN cohort and reasons for exclusion, leading to selection of the study cohort.

For peer review only - http://bmjopen.bmj.com/site/about/guidelines.xhtml

Supplementary Table S2

Drug code related to single-ingredient vitamin D products

2a. Drug code related to single-ingredient vitamin D products

Drug code	Description
29742978	colecalciferol 800unit capsules
30212978	colecalciferol 2,500units/drop oral drops sugar free
30582978	colecalciferol 40,000unit tablets
30643978	colecalciferol 800unit capsules
32236978	colecalciferol 4,000unit tablets
32237978	colecalciferol 4,000unit tablets
32265978	colecalciferol 4,000unit capsules
32267978	colecalciferol 1,000unit tablets
32317978	colecalciferol 50,000unit capsules
34135978	colecalciferol 3,000unit tablets
34138978	colecalciferol 800unit capsules
34144978	colecalciferol 1,000unit capsules
38846978	colecalciferol 20,000unit tablets
39354978	colecalciferol 50,000units/1ml oral solution unit dose ampoules sugar free
39355978	colecalciferol 50,000units/1ml oral solution unit dose ampoules sugar free
47464978	colecalciferol 2,740units/ml oral drops sugar free
47465978	colecalciferol 2,740units/ml oral drops sugar free
48011978	colecalciferol 15,000units/5ml oral solution
51017978	colecalciferol 15,000units/5ml oral solution
51018978	colecalciferol 15,000units/5ml oral solution
51473978	colecalciferol 200units/drop oral drops sugar free
51484978	colecalciferol 1,000unit capsules
52073979	colecalciferol 40,000unit capsules
52074979	colecalciferol 40,000unit capsules
52154979	colecalciferol 20,000unit capsules
53089979	colecalciferol 15,000units/5ml oral solution
53090979	colecalciferol 15,000units/5ml oral solution
53091979	colecalciferol 15,000units/5ml oral solution
53092979	colecalciferol 15,000units/5ml oral solution
53321979	ergocalciferol 125microgram tablets
53322979	ergocalciferol 125microgram tablets
53352979	colecalciferol 20,000unit tablets
53353979	colecalciferol 5,000unit tablets
53355979	colecalciferol 400unit tablets
53356979	colecalciferol 30,000unit capsules
53357979	colecalciferol 5,000unit capsules
53358979	colecalciferol 3,000unit capsules
53359979	ergocalciferol 12.5microgram tablets
53362979	colecalciferol 400unit capsules
53365979	colecalciferol 1,000unit capsules
53366979	colecalciferol 600unit capsules
53367979	colecalciferol 2,200unit capsules
53368979	colecalciferol 10,000unit capsules
53369979	colecalciferol 20,000unit capsules
53370979	colecalciferol 20,000unit capsules
53371979	colecalciferol 50,000unit capsules
53377978	colecalciferol 2,400units/ml oral drops sugar free
53378978	colecalciferol 2,400units/ml oral drops sugar free
53461978	ergocalciferol 12.5microgram tablets
53464978	colecalciferol 20,000unit capsules
53641979	ergocalciferol 250microgram tablets
53650979	ergocalciferol 1.25mg capsules
54637979	colecalciferol 500unit capsules
54638979	colecalciferol 500unit capsules
54716979	colecalciferol 1,000unit capsules
54790979	ergocalciferol 1.25mg capsules
54889979	colecalciferol 3,000units/ml oral solution
54890979	colecalciferol 2,000units/ml oral solution

For peer review only

2a. Drug code related to single-ingredient vitamin D products - continued

Drug code	Description
54891979	colecalciferol 2,000units/ml oral solution
54916979	colecalciferol 800unit tablets
54917979	colecalciferol 800unit tablets
55053979	colecalciferol 3,000unit tablets
55054979	colecalciferol 20,000unit tablets
55055979	colecalciferol 2,200unit tablets
55056979	colecalciferol 10,000unit tablets
55060979	colecalciferol 5,000unit tablets
55061979	ergocalciferol 3,000units/ml oral solution
55062979	ergocalciferol 3,000units/ml oral solution sugar free
55064979	colecalciferol 3,000units/ml oral solution sugar free
55065979	colecalciferol 20,000units/ml oral solution sugar free
55066979	colecalciferol 30,000unit capsules
55067979	colecalciferol 30,000unit capsules
55068979	colecalciferol 5,000unit capsules
55070979	colecalciferol 400unit capsules
55072979	colecalciferol 1,000unit capsules
55074979	colecalciferol 2,200unit capsules
55076979	colecalciferol 10,000unit capsules
55077979	colecalciferol 20,000unit capsules
55078979	colecalciferol 50,000unit capsules
55079979	colecalciferol 50,000unit capsules
55194978	colecalciferol 10,000units/ml oral solution sugar free
55195978	colecalciferol 10,000units/ml oral drops sugar free
55196978	colecalciferol 10,000units/ml oral drops sugar free
55197978	colecalciferol 10,000units/ml oral drops sugar free
55257978	colecalciferol 20,000unit capsules
55558979	ergocalciferol 50,000unit capsules
56123979	ergocalciferol 1.25mg capsules
56242979	colecalciferol 3,000unit tablets
56244979	colecalciferol 20,000unit tablets
56246979	colecalciferol 2,200unit tablets
56248979	colecalciferol 10,000unit tablets
56310979	colecalciferol 400unit tablets
56313979	colecalciferol 1,000unit tablets
56949978	colecalciferol 25,000unit tablets
56950978	colecalciferol 25,000unit tablets
57024978	colecalciferol 1,000unit tablets
57965979	colecalciferol 20,000unit capsules
58139979	colecalciferol 5,000unit tablets
58209979	ergocalciferol 3,000units/ml oral solution
58346979	colecalciferol 800unit capsules
58349979	colecalciferol 5,000unit capsules
58353979	colecalciferol 1,000unit capsules
58355979	colecalciferol 20,000unit capsules
58356979	colecalciferol 20,000unit capsules
58768979	colecalciferol 2,000units/ml oral solution sugar free
58769979	colecalciferol 2,000units/ml oral solution sugar free
58782979	colecalciferol 3,000units/ml oral solution sugar free
59522978	colecalciferol 2,000unit tablets
59525978	colecalciferol 40,000unit capsules
59527978	colecalciferol 5,000unit tablets
59528978	colecalciferol 20,000unit capsules
59769979	colecalciferol 800units/ml oral drops sugar free
59770979	colecalciferol 800units/ml oral drops sugar free
59771979	colecalciferol 400units/dose oral spray sugar free
59772979	colecalciferol 400units/dose oral spray sugar free
59843979	colecalciferol 20,000unit capsules
59981979	ergocalciferol 15,000units/5ml oral solution
60056979	ergocalciferol 300,000units/1ml solution for injection ampoules

For peer review only

2a. Drug code related to single-ingredient vitamin D products - continued

Drug code	Description
60224979	colecalciferol 300,000units/1ml solution for injection ampoules
60225979	ergocalciferol 300,000units/1ml solution for injection ampoules
60373979	fultium-d3 800unit capsules
60374979	colecalciferol 800unit capsules
60566979	vitamin d2 3,000units/ml oral solution sugar free
60568979	ergocalciferol 20,000units/ml oral solution sugar free
60572979	colecalciferol 20,000units/ml oral solution sugar free
60594979	colecalciferol 1,000unit tablets
61563979	colecalciferol 30,000units/5ml oral solution
61592979	colecalciferol 2,000units/ml oral drops sugar free
61593979	colecalciferol 2,000units/ml oral drops sugar free
61910979	colecalciferol 400unit tablets
61911979	colecalciferol 400unit tablets
62001979	colecalciferol 1,000units/ml oral solution
62002979	colecalciferol 1,000units/ml oral solution
62087979	colecalciferol 10,000units/ml oral solution
62088979	colecalciferol 10,000units/ml oral solution
62116979	colecalciferol 5,000unit capsules
62118979	colecalciferol 3,000unit capsules
62152979	colecalciferol 6,000units/5ml oral suspension
62324979	colecalciferol 20,000units/ml oral drops
62356979	ergocalciferol 12.5microgram tablets
62357979	ergocalciferol 12.5microgram tablets
62536979	colecalciferol 400unit capsules
62622979	colecalciferol 1,000unit tablets
62626979	colecalciferol 1,000unit capsules
62643979	colecalciferol 600unit capsules
62645979	colecalciferol 2,200unit capsules
62986979	colecalciferol 20,000unit capsules
63201979	colecalciferol 2,000unit capsules
64131979	colecalciferol 4,000units/5ml oral suspension
68364979	ergocalciferol 400units/5ml oral solution
68382979	ergocalciferol 20,000units/5ml oral suspension
68384979	ergocalciferol 20,000units/5ml oral solution
68392979	ergocalciferol 120units/5ml oral solution
68396979	ergocalciferol 100,000units/5ml oral solution
68780978	colecalciferol 20,000unit capsules
68781978	colecalciferol 20,000unit capsules
69915979	colecalciferol 400units/5ml oral suspension
69917979	colecalciferol 20,000units/5ml oral suspension
69920979	colecalciferol 1,000units/5ml oral suspension
70249978	ergocalciferol 1.25mg capsules
73011978	colecalciferol 25,000units/1ml oral solution unit dose ampoules sugar free
73012978	colecalciferol 25,000units/1ml oral solution unit dose ampoules sugar free
73102978	colecalciferol 3,200unit capsules
73128978	colecalciferol 10,000unit capsules
73129978	colecalciferol 20,000unit capsules
73301978	colecalciferol 20,000units/ml oral solution
73391978	colecalciferol 3,200unit capsules
73392978	colecalciferol 3,200unit capsules
77093978	colecalciferol 15,000units/5ml oral solution
78387978	colecalciferol 500unit orodispersible tablets sugar free
78388978	colecalciferol 500unit orodispersible tablets sugar free
78389978	colecalciferol 2,000unit orodispersible tablets sugar free
78390978	colecalciferol 2,000unit orodispersible tablets sugar free
78426978	colecalciferol 2,200unit tablets
78428978	colecalciferol 5,000unit tablets
78441978	colecalciferol 1,000unit capsules
78719978	colecalciferol 2,000unit tablets
78720978	colecalciferol 2,000unit tablets

For peer review only

2a. Drug code related to single-ingredient vitamin D products - continued

Drug code	Description
79321978	colecalciferol 200units/drop oral drops sugar free
79322978	colecalciferol 200units/drop oral drops sugar free
79338978	ergocalciferol 6,000units/5ml oral solution
79340978	ergocalciferol 10,000units/5ml oral solution
79935978	ergocalciferol 600,000units/1.5ml solution for injection ampoules
79960978	colecalciferol 280unit chewable tablets
79961978	colecalciferol 280unit chewable tablets
79993978	colecalciferol 20,000unit tablets
80016978	colecalciferol 400unit capsules
80018978	colecalciferol 1,000unit tablets
80019978	colecalciferol 1,000unit capsules
80022978	colecalciferol 20,000unit capsules
80147979	ergocalciferol 800units/5ml oral suspension
80151979	ergocalciferol 6,000units/5ml oral suspension
80153979	ergocalciferol 50,000units/5ml oral suspension
80157979	ergocalciferol 30,000units/5ml oral suspension
80159979	ergocalciferol 3,000units/5ml oral suspension
80169979	ergocalciferol 10,000units/5ml oral suspension
80416979	colecalciferol 15,000units/5ml oral suspension
80883998	colecalciferol 3,000units/ml oral solution sugar free
80885998	colecalciferol 3,000units/ml oral solution sugar free
80886998	colecalciferol 20,000units/ml oral solution sugar free
80887998	colecalciferol 20,000units/ml oral solution sugar free
80914998	colecalciferol 2,000units/ml oral drops sugar free
80915998	colecalciferol 2,000units/ml oral drops sugar free
80947998	ergocalciferol 20,000units/ml oral solution sugar free
80952998	colecalciferol 800unit capsules
80958998	colecalciferol 20,000unit capsules
81004998	colecalciferol 30,000unit capsules
81007998	colecalciferol 30,000unit capsules
81009998	colecalciferol 10,000unit capsules
81010998	colecalciferol 10,000unit capsules
81011998	colecalciferol 2,500unit capsules
81012998	colecalciferol 2,500unit capsules
81013998	colecalciferol 1,000unit capsules
81014998	colecalciferol 1,000unit capsules
81015998	colecalciferol 400unit capsules
81074998	colecalciferol 5,000unit capsules
81138998	colecalciferol 500 units tablets
81252998	colecalciferol 1,000unit tablets
81253998	colecalciferol 1,000unit tablets
81328998	colecalciferol 800unit capsules
81329998	colecalciferol 800unit capsules
81335998	colecalciferol 20,000unit capsules
81464998	colecalciferol 400unit capsules
81568998	colecalciferol 10000units/ml oral solution
81612998	colecalciferol 1,000unit tablets
81624998	colecalciferol 10,000units/5ml oral suspension
81631998	colecalciferol 2,200unit capsules
81710998	colecalciferol 50,000unit capsules
82283998	colecalciferol 15,000units/5ml oral solution
82513998	colecalciferol 20,000unit capsules
82514998	ergocalciferol 1.25mg capsules
82946978	colecalciferol 20,000unit tablets
82956978	colecalciferol 20,000unit capsules
83392978	colecalciferol 50,000unit tablets
83393978	colecalciferol 50,000unit tablets
83468978	colecalciferol 20,000unit tablets
83469978	colecalciferol 10,000unit tablets
83480978	colecalciferol 800unit capsules

For peer review only

2a. Drug code related to single-ingredient vitamin D products - continued

Drug code	Description
83743978	ergocalciferol 1,000units/5ml oral solution
83744978	colecalciferol 5,000units/5ml oral solution
83746978	colecalciferol 5,000units/5ml oral solution
83747978	colecalciferol 10,000units/5ml oral solution
83748978	colecalciferol 10,000units/5ml oral solution
83750978	colecalciferol 10,000units/5ml oral solution
84072978	colecalciferol 5,000unit tablets
84414978	ergocalciferol 1,500units/ml oral solution sugar free
84586978	colecalciferol 20,000unit tablets
84592978	colecalciferol 400unit tablets
84600978	colecalciferol 1,000unit tablets
84680998	colecalciferol oral liquid
85005998	ergocalciferol oral liquid
85220998	ergocalciferol 600,000units/2ml solution for injection ampoules
85224998	ergocalciferol 300,000units/1ml solution for injection ampoules
85329978	colecalciferol 1,000unit tablets
86614979	ergocalciferol 300,000units/1ml solution for injection ampoules
86615979	ergocalciferol 300,000units/1ml solution for injection ampoules
93163990	ergocalciferol 600,000units/2ml solution for injection ampoules
93164990	ergocalciferol 300,000units/1ml solution for injection ampoules
93384992	calciferol 10 mcg tab
93390992	calciferol 1,000 iu i/u tab
93401992	calciferol 5000 i/u tab
94809990	ergocalciferol 1.25mg tablets
94810990	ergocalciferol 250microgram tablets
95606992	ergocalciferol 600ku/1.5ml solution
95872992	calciferol 3,000 iu tab
95873992	calciferol 30,000 iu/ml inj
95875992	calciferol soln 400u/5ml in arachis oil sol
95877992	calciferol 40,000 iu/ml inj
95878992	calciferol 400 iu/ml sol
96131992	calciferol 400 iu/ml i/u syr
96132992	calciferol .25 mg cap
96133992	calciferol 1 mg cap
96134992	calciferol 1,000 iu i/u cap
96599992	calciferol 75 mcg inj
96600992	ergocalciferol 250microgram tablets
96601992	calciferol 20,000 iu cap
96788998	ergocalciferol 400000units/ml oral solution
97023992	calciferol 100,000 iu/ml inj
97024992	calciferol 600,000 iu/ml inj
97032992	calciferol 300 iu/ml i/u syr
97033992	calciferol drops liq
97034992	calciferol 400 i/u inj
97035992	calciferol 9,000 iu/ml liq
97036992	calciferol 1,000 iu/5ml sol
97037992	calciferol 300 mg tab
97038992	calciferol 15 mcg tab
97238989	ergocalciferol 1.25mg tablets
97238990	ergocalciferol 250microgram tablets
97767998	calciferol bp 3000units/ml solution
98374992	vitamin d2 tab
98421997	ergocalciferol 1.25mg tablets
98421998	ergocalciferol 250microgram tablets
98422997	ergocalciferol 1,000units/5ml oral suspension
98422998	ergocalciferol 3,000units/ml oral solution sugar free
98925998	ergocalciferol 600,000units/2ml solution for injection ampoules
99762990	ergocalciferol 250microgram tablets
99763988	ergocalciferol 250microgram tablets
99763990	ergocalciferol 300,000units/1ml solution for injection ampoules

For peer review only

2a. Drug code related to combination vitamin D products

Drug code	Description
34026978	colecalciferol 1,000unit / calcium carbonate 2.5g chewable tablets
34027978	colecalciferol 1,000unit / calcium carbonate 2.5g chewable tablets
52015979	colecalciferol 800unit / calcium carbonate 1.25g chewable tablets
52016979	colecalciferol 800unit / calcium carbonate 1.25g tablets
56947978	colecalciferol 800unit / calcium carbonate 2.5g chewable tablets
56948978	colecalciferol 800unit / calcium carbonate 2.5g chewable tablets
58270979	generic caldrink d3 liquid
60543979	colecalciferol 100unit / calcium carbonate 400mg chewable tablets
60544979	colecalciferol 100unit / calcium carbonate 400mg chewable tablets
61594979	colecalciferol 150unit / calcium 250mg tablets
61595979	colecalciferol 150unit / calcium 250mg tablets
64223979	calcium carbonate 400mg / ergocalciferol 2.5microgram tablets
64224979	calcium carbonate 400mg / ergocalciferol 2.5microgram tablets
64830979	calcium and ergocalciferol tablets
64831979	colecalciferol 100unit / calcium carbonate 400mg tablets
68756978	colecalciferol 880unit / calcium carbonate 2.5g chewable tablets
68757978	colecalciferol 880unit / calcium carbonate 2.5g chewable tablets
73294978	generic healthy start children's vitamin drops
73598978	colecalciferol 100unit / calcium carbonate 400mg tablets
80849998	calcium carbonate 400mg with colecalciferol 100 units chewable tablets
81191998	colecalciferol 400unit / calcium carbonate 1.5g tablets
81268998	colecalciferol 200unit / calcium carbonate 750mg tablets
81269998	colecalciferol 200unit / calcium carbonate 750mg tablets
81655998	colecalciferol 800unit / calcium carbonate 1.25g chewable tablets
81656998	colecalciferol 800unit / calcium carbonate 1.25g chewable tablets
82214998	colecalciferol 400unit / calcium carbonate 1.25g tablets
82215998	colecalciferol 400unit / calcium carbonate 1.25g chewable tablets
82229978	generic littlevit multivitamin drops
82344998	colecalciferol 400unit / calcium carbonate 1.5g tablets
82429979	colecalciferol 400unit / calcium carbonate 1.25g chewable tablets
82483998	colecalciferol 400unit / calcium carbonate 1.5g chewable tablets
82546978	colecalciferol 400unit / calcium carbonate 1.5g chewable tablets
82752998	generic sandocal+d 1200 effervescent tablets
82753998	calcium lactate gluconate with calcium carbonate and colecalciferol 1200mg calcium with 800iu effervescent tablets
82966998	generic sandocal+d 600 effervescent tablets
82967998	calcium lactate gluconate with calcium carbonate and colecalciferol 600mg calcium with 400iu effervescent tablets
83420998	colecalciferol 400unit / calcium carbonate 1.25g chewable tablets
83638978	colecalciferol 1,000unit / folic acid 400microgram capsules
83639978	colecalciferol 1,000unit / folic acid 400microgram capsules
84212998	risedronate sodium 35mg with calcium carbonate 2500mg & colecalciferol 22micrograms tablets and granules
84286998	colecalciferol 400unit / calcium carbonate 1.5g effervescent tablets
84287998	colecalciferol 400unit / calcium carbonate 1.5g chewable tablets
84292998	colecalciferol 400unit / calcium carbonate 1.5g chewable tablets
84362998	vitamins c & d3 with folic acid tablets
84363998	vitamins c & d3 with folic acid tablets
86598998	alendronic acid 70mg / colecalciferol 70microgram tablets
86599998	alendronic acid 70mg / colecalciferol 70microgram tablets
88002998	dolomite with vitamin d tablets
88144997	calcium carbonate 600mg with vitamin d % micrograms with minerals chewable tablets
88144998	calcium carbonate 600mg with vitamin d and minerals 5 micrograms tablets
88225998	calcium carbonate 600mg with vitamin d and 5 micrograms tablets
88233979	generic abidec multivitamin drops
89183998	colecalciferol 400unit / calcium carbonate 1.25g chewable tablets
89439998	colecalciferol 440unit / calcium carbonate 1.25g effervescent granules sachets
89637998	colecalciferol 400unit / calcium carbonate 1.5g chewable tablets
89647998	colecalciferol 400unit / calcium carbonate 1.5g chewable tablets
89828997	multivitamin and mineral chewable tablets

For peer review only - http://bmjopen.bmj.com/site/about/guidelines.xhtml

2a. Drug code related to combination vitamin D products - continued

Drug code	Description
89828998	calcium carbonate 600mg with vitamin d and minerals 5 micrograms tablets
90498998	nicotinic acid with colecalciferol and calcium gluconate tablet
91053998	colecalciferol 800unit / calcium phosphate 3.1g oral powder sachets
91526998	calcium carbonate 600mg with vitamin d and 5 micrograms tablets
91987979	colecalciferol 400unit / calcium carbonate 1.5g chewable tablets
91989979	colecalciferol 400unit / calcium carbonate 1.25g chewable tablets
91990979	colecalciferol 400unit / calcium carbonate 1.25g chewable tablets
91991979	colecalciferol 400unit / calcium carbonate 1.25g chewable tablets
91993979	colecalciferol 400unit / calcium carbonate 1.25g chewable tablets
91994979	colecalciferol 400unit / calcium carbonate 1.25g chewable tablets
91995979	colecalciferol 200unit / calcium carbonate 1.25g chewable tablets
91996979	colecalciferol 400unit / calcium carbonate 1.25g chewable tablets
91997979	colecalciferol 400unit / calcium carbonate 1.25g chewable tablets
91998979	colecalciferol 200unit / calcium carbonate 1.25g chewable tablets
92000979	colecalciferol 200unit / calcium carbonate 1.25g chewable tablets
92001979	colecalciferol 200unit / calcium carbonate 1.25g chewable tablets
92004979	calcium and ergocalciferol tablets
93127992	calcium 500mgs & vitamin d 400iu tab
93276998	generic abidec multivitamin drops
94021990	calcium and ergocalciferol tablets
94089992	calcium + vitamin d 300 mg tab
94756992	calcium & vitamin d 6000 i/u tab
96015992	vit.a /vit.b1 /vit.b2 /vit.c /vit.d2 /ni pow
96020992	calcium and ergocalciferol tablets
96737998	calciferol with calcium tablets
96789998	calcium and ergocalciferol tablets
96897998	calcium and ergocalciferol tablets
96920998	colecalciferol 800unit / calcium phosphate 3.1g oral powder sachets
96959996	colecalciferol 440unit / calcium carbonate 1.25g effervescent granules sachets
96959997	colecalciferol 400unit / calcium carbonate 1.25g chewable tablets
96959998	colecalciferol 200unit / calcium carbonate 1.25g chewable tablets
97031992	calciferol/calcium gluconate tab
97064992	calcium 500mgs & vitamin d 200iu i/u tab
97138998	calcium and ergocalciferol tablets
97248990	calcium and ergocalciferol tablets
97780990	calcium and ergocalciferol tablets
97815990	calcium and ergocalciferol tablets
97935998	generic abidec multivitamin drops
98247992	vit.a /vit.b1 /vit.b2 /vit.c /vit.d2 /ni eli
98249990	calcium and ergocalciferol tablets
98581990	calcium and ergocalciferol tablets
98700997	colecalciferol 200unit / calcium carbonate 1.25g chewable tablets
98700998	colecalciferol 200unit / calcium carbonate 1.25g chewable tablets
99018990	calcium and ergocalciferol tablets
99733992	ascorbic acid/vitamin a/vitamin d 2 25 mg tab

For peer review only

Supplementary Table S3

Read codes related to vitamin D deficiency and 25-hydroxyvitamin D tests

Read code	Description
C28..00	vitamin d deficiency
C28..11	osteomalacia
C28..12	rickets
C280.00	active rickets
C282.00	osteomalacia unspecified
C28z.00	avitaminosis d nos
C2B..00	vitamin d insufficiency
J690.11	coeliac rickets
K080300	renal rickets
44LA.00	serum vitamin d
44LM.00	plasma 25-hydroxyvitamin d3 level
44LP.00	serum 25-hydroxy vitamin d3 level
44Lg.00	serum vitamin d2 level
44Ln.00	serum 25-hydroxyvitamin d2 level
4QB4.00	vitamin d level
4QB4000	vitamin d2 level
4QB4100	vitamin d3 level
4QB4200	25-hydroxyvitamin d2 level
4QB4300	25-hydroxyvitamin d3 level
4QB4400	combined total vitamin d2 and d3 level
4QB4500	25-hydroxyvitamin d level
4QB4600	total 25-hydroxyvitamin d level
4QB4700	serum total 25-hydroxy vitamin d level
5788	radiobioassay - vitamin d

For peer review only

Supplementary Table S4

No. of children with a record of diagnosis Read codes suggestive of vitamin D deficiency in the 90 days prior to or on the date of the incident prescription, stratified by pre-supplementation 25-hydroxyvitamin D concentrations

	**25-hydroxyvitamin D concentrations at initiation of supplementation (nmol/L)**							
	**<10** (n=3,738)	**10 - 20** (n=3,898)	**>20 - 30** (n=441)	**>30 - 50** (n=31)	**>50** (n=9)	**No test** (n=3,986)	**Undetermined** (n=174)	**Total** (n=12,277)
**No. of children with symptoms, n**								
Musculoskeletal and non-specific pain	750	769	67	4	0	340	23	1,953
Tiredness and fatigue	556	547	46	3	0	106	5	1,263
Failure to thrive	25	33	7	1	0	36	3	105
Bone fracture	13	15	2	1	0	42	6	79
Skeletal deformity	15	26	8	1	0	35	2	87
Numbness or paraesthesia	14	9	1	0	0	7	0	31
Hypocalcaemia	9	2	2	0	0	16	0	29
Seizure or tetany	1	3	0	0	0	16	1	21
Abnormal gait	4	9	1	0	0	6	1	21
Muscle weakness	9	7	2	0	0	3	0	21
Delay in motor development	0	0	0	0	0	5	0	5
Cardiomyopathy	0	0	0	0	0	2	0	2
**Total, n (%)**	1,396 (37.3)	1,420 (36.4)	136 (30.8)	10 (32.3)	0 (0)	614 (15.4)	41 (23.6)	3,617 (29.5)

For peer review only

Supplementary Table S5

Multivariable Poisson regression models of incidence of vitamin D supplementation prescribing in children stratified by sex (complete-case analysis)

	Male	Female
	IRR[a] (95% CI)	IRR[a] (95% CI)
**Age group**		
Up to 6 months	1.00 (Reference)	1.00 (Reference)
6 months - 4 years	2.33 (1.11 – 4.92)	1.37 (0.73 – 2.56)
5 - 11 years	3.08 (1.47 – 6.49)	2.42 (1.30 – 4.51)
12 - 17 years	5.35 (2.54 – 11.24)	8.15 (4.38 – 15.17)
**Ethnicity**		
White	1.00 (Reference)	1.00 (Reference)
Asian	6.59 (5.92 – 7.33)	6.24 (5.71 – 6.82)
Black	3.67 (3.21 – 4.19)	3.63 (3.26 – 4.05)
Others	3.47 (2.79 – 4.32)	3.79 (3.18 – 4.51)
Mixed	2.32 (1.88 – 2.86)	2.31 (1.94 – 2.75)
**Townsend index**		
1 (least deprived)	1.00 (Reference)	1.00 (Reference)
2	1.03 (0.88 – 1.21)	0.97 (0.85 – 1.11)
3	1.14 (0.98 – 1.33)	1.12 (0.98 – 1.27)
4	1.32 (1.14 – 1.53)	1.31 (1.16 – 1.49)
5 (most deprived)	1.33 (1.13 – 1.56)	1.41 (1.24 – 1.61)
**Country**		
England	1.00 (Reference)	1.00 (Reference)
Wales	0.28 (0.18 – 0.42)	0.31 (0.21 – 0.45)
Scotland	0.31 (0.23 – 0.40)	0.23 (0.17 – 0.30)
Northern Ireland	0.37 (0.23 – 0.61)	0.22 (0.13 – 0.38)
**Year**		
2008	1.00 (Reference)	1.00 (Reference)
2009	2.06 (1.30 – 3.26)	3.03 (1.98 – 4.66)
2010	2.88 (1.87 – 4.45)	4.68 (3.11 – 7.04)
2011	4.70 (3.11 – 7.11)	7.87 (5.30 – 11.68)
2012	9.62 (6.46 – 14.34)	15.19 (10.32 – 22.37)
2013	14.37 (9.68 – 21.32)	20.84 (14.18 – 30.63)
2014	17.62 (11.87 – 26.16)	26.00 (17.69 – 38.22)
2015	18.02 (12.10 – 26.85)	29.35 (19.92 – 43.25)
2016	20.07 (13.43 – 29.98)	31.74 (21.49 – 46.88)

Abbreviations: CI, confidence interval; IRR = incidence rate ratio.
[a]Adjusted for all variables listed in the table. The multilevel models included the general practice as a random effect.

For peer review only

Supplementary Figure S2

Time trends in vitamin D supplementation prescribing in children by product types (n=11,743)

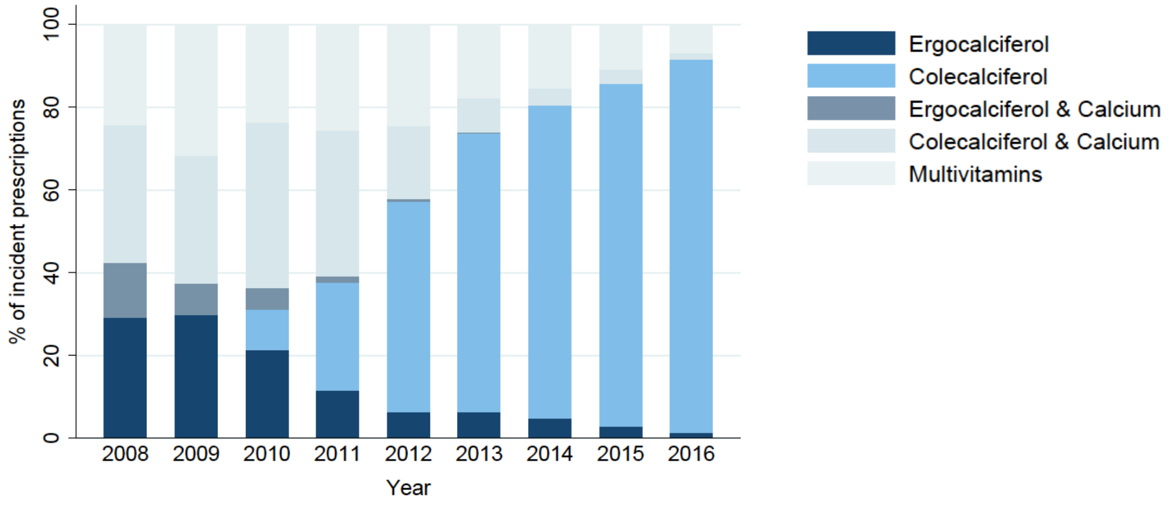

peer review only

Supplementary Table S6

Doses of vitamin D supplementation prescribed stratified by age group

	**Age group**			
	**Up to 6 months (n = 29)**	**6 months – 4 years (n = 1,670)**	**5 - 11 years (n = 3,504)**	**12 - 17 years (n = 6,540)**
Equivalent daily dose, n (%)	25 (86.2%)	1,460 (87.4%)	3,193 (91.1%)	5,929 (90.7%)
≤400 IU/day	8	707	1,110	640
401 – 1,000 IU/day	1	178	625	2,012
1,001 – 3,000 IU/day	15	170	437	1,073
3,001 – 6,000 IU/day	1	377	869	1,082
6,001 – 10,000 IU/day	0	25	129	1,016
>10,000 IU/day	0	3	23	106
Stoss therapy, n (%)	0	24 (1.7%)	94 (2.7%)	437 (6.7%)
<150,000 IU	0	10	32	86
150,000 – 300,000 IU	0	14	61	290
>300,000 IU	0	0	1	61
Undetermined, n (%)	4 (13.8%)	186 (11.1%)	217 (6.2%)	174 (2.7%)

For peer review only

For peer review only - http://bmjopen.bmj.com/site/about/guidelines.xhtml

Supplementary Table S7

Vitamin D supplementation dosing frequencies observed in the study cohort (n=11,743)

Dosing Frequencies	n (%)
Once daily	7,376 (62.8)
Once every 7 days	1,337 (11.4)
Twice a day	1,012 (8.6)
Undetermined	581(5)
Stoss	555 (4.7)
Once 2 times a week	267 (2.3)
Once every 14 days	227 (1.9)
Once 3 times a week	157 (1.3)
Once every 2 days	93 (0.8)
Once every 28 days	41 (0.4)
3 times a day	35 (0.3)
Once every 42 days	19 (0.2)
Others	43 (0.4)

For peer review only

The RECORD statement – checklist of items, extended from the STROBE statement, that should be reported in observational studies using routinely collected health data.

	Item No.	STROBE items	Location in manuscript where items are reported	RECORD items	Location in manuscript where items are reported
**Title and abstract**					
	1	(a) Indicate the study's design with a commonly used term in the title or the abstract (b) Provide in the abstract an informative and balanced summary of what was done and what was found		RECORD 1.1: The type of data used should be specified in the title or abstract. When possible, the name of the databases used should be included.<br><br>RECORD 1.2: If applicable, the geographic region and timeframe within which the study took place should be reported in the title or abstract.<br><br>RECORD 1.3: If linkage between databases was conducted for the study, this should be clearly stated in the title or abstract.	1,2<br><br>1,2<br><br>N/A
**Introduction**					
Background rationale	2	Explain the scientific background and rationale for the investigation being reported			4,5
Objectives	3	State specific objectives, including any prespecified hypotheses			4,5
**Methods**					
Study Design	4	Present key elements of study design early in the paper			5,6
Setting	5	Describe the setting, locations, and relevant dates, including periods of recruitment, exposure, follow-up, and data collection			5,6

For peer review only

Participants	6	*(a) Cohort study* - Give the eligibility criteria, and the sources and methods of selection of participants. Describe methods of follow-up<br>*Case-control study* - Give the eligibility criteria, and the sources and methods of case ascertainment and control selection. Give the rationale for the choice of cases and controls<br>*Cross-sectional study* - Give the eligibility criteria, and the sources and methods of selection of participants<br><br>*(b) Cohort study* - For matched studies, give matching criteria and number of exposed and unexposed<br>*Case-control study* - For matched studies, give matching criteria and the number of controls per case		RECORD 6.1: The methods of study population selection (such as codes or algorithms used to identify subjects) should be listed in detail. If this is not possible, an explanation should be provided.<br><br>RECORD 6.2: Any validation studies of the codes or algorithms used to select the population should be referenced. If validation was conducted for this study and not published elsewhere, detailed methods and results should be provided.<br><br>RECORD 6.3: If the study involved linkage of databases, consider use of a flow diagram or other graphical display to demonstrate the data linkage process, including the number of individuals with linked data at each stage.	6, 7, Data sharing, Supplementary<br><br>N/A<br><br>N/A
Variables	7	Clearly define all outcomes, exposures, predictors, potential confounders, and effect modifiers. Give diagnostic criteria, if applicable.		RECORD 7.1: A complete list of codes and algorithms used to classify exposures, outcomes, confounders, and effect modifiers should be provided. If these cannot be reported, an explanation should be provided.	6,7,8 Supplementary
Data sources/ measurement	8	For each variable of interest, give sources of data and details of methods of assessment (measurement).<br>Describe comparability of assessment methods if there is more than one group			6,7,8

For peer review only

Bias	9	Describe any efforts to address potential sources of bias			6,7,8
Study size	10	Explain how the study size was arrived at			5,6, Supplementary
Quantitative variables	11	Explain how quantitative variables were handled in the analyses. If applicable, describe which groupings were chosen, and why			6,7,8,9
Statistical methods	12	(a) Describe all statistical methods, including those used to control for confounding<br>(b) Describe any methods used to examine subgroups and interactions<br>(c) Explain how missing data were addressed<br>(d) *Cohort study* - If applicable, explain how loss to follow-up was addressed<br>*Case-control study* - If applicable, explain how matching of cases and controls was addressed<br>*Cross-sectional study* - If applicable, describe analytical methods taking account of sampling strategy<br>(e) Describe any sensitivity analyses			9,10
Data access and cleaning methods		..		RECORD 12.1: Authors should describe the extent to which the investigators had access to the database population used to create the study population.	18

For peer review only

				RECORD 12.2: Authors should provide information on the data cleaning methods used in the study.	
Linkage		..		RECORD 12.3: State whether the study included person-level, institutional-level, or other data linkage across two or more databases. The methods of linkage and methods of linkage quality evaluation should be provided.	N/A
**Results**					
Participants	13	(a) Report the numbers of individuals at each stage of the study (*e.g.*, numbers potentially eligible, examined for eligibility, confirmed eligible, included in the study, completing follow-up, and analysed)<br>(b) Give reasons for non-participation at each stage.<br>(c) Consider use of a flow diagram		RECORD 13.1: Describe in detail the selection of the persons included in the study (*i.e.,* study population selection) including filtering based on data quality, data availability and linkage. The selection of included persons can be described in the text and/or by means of the study flow diagram.	5,6
Descriptive data	14	(a) Give characteristics of study participants (*e.g.*, demographic, clinical, social) and information on exposures and potential confounders<br>(b) Indicate the number of participants with missing data for each variable of interest<br>(c) *Cohort study* - summarise follow-up time (*e.g.*, average and total amount)			10<br>Table 1
Outcome data	15	*Cohort study* - Report numbers of outcome events or summary measures over time<br>*Case-control study* - Report numbers in each exposure			10,11,12,13<br>Table 2<br>Figure 1<br>Figure 2<br>Figure 3

For peer review only

		category, or summary measures of exposure<br>*Cross-sectional study* - Report numbers of outcome events or summary measures			
Main results	16	(a) Give unadjusted estimates and, if applicable, confounder-adjusted estimates and their precision (e.g., 95% confidence interval). Make clear which confounders were adjusted for and why they were included<br>(b) Report category boundaries when continuous variables were categorized<br>(c) If relevant, consider translating estimates of relative risk into absolute risk for a meaningful time period			10,11,12,13
Other analyses	17	Report other analyses done—e.g., analyses of subgroups and interactions, and sensitivity analyses			10,11,12,13
**Discussion**					
Key results	18	Summarise key results with reference to study objectives			13
Limitations	19	Discuss limitations of the study, taking into account sources of potential bias or imprecision. Discuss both direction and magnitude of any potential bias		RECORD 19.1: Discuss the implications of using data that were not created or collected to answer the specific research question(s). Include discussion of misclassification bias, unmeasured confounding, missing data, and changing eligibility over time, as they pertain to the study being reported.	16
Interpretation	20	Give a cautious overall interpretation of results considering objectives,			13, 14, 15

For peer review only - http://bmjopen.bmj.com/site/about/guidelines.xhtml

		limitations, multiplicity of analyses, results from similar studies, and other relevant evidence			
Generalisability	21	Discuss the generalisability (external validity) of the study results			16
**Other Information**					
Funding	22	Give the source of funding and the role of the funders for the present study and, if applicable, for the original study on which the present article is based			18
Accessibility of protocol, raw data, and programming code		..		RECORD 22.1: Authors should provide information on how to access any supplemental information such as the study protocol, raw data, or programming code.	18

*Reference: Benchimol EI, Smeeth L, Guttmann A, Harron K, Moher D, Petersen I, Sørensen HT, von Elm E, Langan SM, the RECORD Working Committee. The REporting of studies Conducted using Observational Routinely-collected health Data (RECORD) Statement. *PLoS Medicine* 2015; in press.

*Checklist is protected under Creative Commons Attribution (CC BY) license.

For peer review only

Vitamin D prescribing in children in UK primary care practices: a population-based cohort study

Journal:	*BMJ Open*
Manuscript ID	bmjopen-2019-031870.R2
Article Type:	Original research
Date Submitted by the Author:	10-Oct-2019
Complete List of Authors:	Wan, Mandy; Guy's and Saint Thomas' NHS Foundation Trust, Evelina Pharmacy; King's College London, Institute of Pharmaceutical Science<br>Horsfall, Laura; University College London, Research Department of Primary Care and Population Health<br>Basatemur, Emre; University College London, Population, Policy and Practice Programme<br>Patel, Jignesh; King's College London, Institute of Pharmaceutical Science<br>Shroff, Rukshana; Great Ormond Street Hospital For Children NHS Trust,<br>Rait, Greta; University College London, Research Department of Primary Care and Population Health
**Primary Subject Heading**:	Paediatrics
Secondary Subject Heading:	Epidemiology
Keywords:	PAEDIATRICS, Paediatric endocrinology < PAEDIATRICS, EPIDEMIOLOGY

Vitamin D prescribing in children in UK primary care practices: a population-based cohort study

Mandy Wan (ORCID ID: 0000-0001-8802-0425), Laura J Horsfall (ORCID ID: 0000-0002-0868-5660), Emre Basatemur, Jignesh P Patel (ORCID ID: 0000-0003-4197-8294), Rukshana Shroff* (ORCID ID: 0000-0001-8501-1072), and Greta Rait* (ORCID ID: 0000-0002-7216-7294)

*These authors share senior authorship

Mandy Wan: Lead Paediatric Research Pharmacist at Guy's and St Thomas' NHS Foundation Trust, Evelina London Children's Hospital, Pharmacy Department, London, UK; and PhD student at Institute of Pharmaceutical Science, King's College London, London, UK.

Laura Horsfall: Senior Epidemiologist and Wellcome Trust Fellow at Research Department of Primary Care and Population Health, University of College London, London, UK.

Emre Basatemur: Clinical Research Fellow at Population, Policy and Practice Programme, University of College London Institute of Child Health, London, UK.

Jignesh P Patel: Reader and Honorary Consultant Pharmacist in Anticoagulation at Institute of Pharmaceutical Science, King's College London, London, UK and Department of Haematological Medicine, King's College Hospital Foundation NHS Trust, London, UK.

Rukshana Shroff: Consultant Paediatric Nephrologist at Renal Unit, Great Ormond Street Hospital NHS Foundation Trust, London, UK.

Greta Rait: Professor of Primary Care and Health Services Research at Research Department of Primary Care and Population Health, University of College London, London, UK.

Corresponding author: Mandy Wan
Email: mandy.wan@gstt.nhs.uk
Organisation: Guy's and St Thomas' NHS Foundation Trust
Evelina London Children's Hospital
Pharmacy Department
Westminster Bridge Road
London
SE1 7EH

For peer review only

Abstract

Objective To examine temporal changes in the incidence and patterns of vitamin D supplementation prescribing by general practitioners (GPs) between 2008 and 2016.

Design Population-based cohort study.

Setting UK general practice health records from The Health Improvement Network (THIN).

Participants Children aged 0 to 17 years who were registered with their general practices for at least 3 months.

Outcome measures Annual incidence rates of vitamin D prescriptions were calculated, and rate ratios were estimated using multivariable Poisson regression to explore differences by sociodemographic factors. Data on the type of supplementation, dose, dosing schedule, linked 25-hydroxyvitamin D [25(OH)D] laboratory test results, and clinical symptoms suggestive of vitamin D deficiency were analysed.

Results Among 2 million children, the crude annual incidence of vitamin D prescribing increased by 26-fold between 2008 and 2016 rising from 10.8 (95% CI: 8.9, 13.1) to 276.8 (95% CI: 264.3, 289.9) per 100,000 person-years. Older children, non-white ethnicity, and general practices in England (compared to Wales/Scotland/Norther Ireland) were independently associated with higher rates of prescribing. Analyses of incident prescriptions showed inconsistent supplementation regimens with an absence of pre-supplementation 25(OH)D concentrations in 28.7 to 56.4% of prescriptions annually. There was an increasing trend in prescribing at pharmacological doses irrespective of 25(OH)D concentrations, deviating in part from UK recommendations. Prescribing at pharmacological doses for children with deficient status increased from 3.8 to 79.4%, but the rise was also observed in children for whom guidelines recommended prevention doses (0 to 53%). Vitamin D supplementation at pharmacological doses was also prescribed in at least 40% of children with no pre-supplementation 25(OH)D concentrations annually.

Conclusions There has been a marked and sustained increase in vitamin D supplementation prescribing in children in UK primary care. Our data suggests that national guidelines on vitamin D supplementation for children are not consistently followed by GPs.

For peer review only - http://bmjopen.bmj.com/site/about/guidelines.xhtml

Strengths and limitations of this study

- This is the first population-based study to examine the extent and variability of vitamin D supplementation prescribing practice using representative primary care data.
- This study does not contain data from secondary care, however, vitamin D deficiency is largely managed in the primary care setting.
- Although several explanations for the factors that might have contributed to changes in vitamin D supplementation prescribing over time are provided, it was not possible to determine the practitioners' rationale for prescribing, nor could we assess clinical appropriateness of prescriptions on an individual level.

For peer review only

Introduction

Vitamin D is an essential prohormone that humans obtain through cutaneous photosynthesis, diet, or supplementation. While few would argue the importance of its physiological role in maintaining calcium homeostasis and bone mineralisation, the beneficial effects of vitamin D supplementation is a topic of much controversy.[1–5] In children, severe vitamin D deficiency can cause rickets and hypocalcaemic seizures, but the clinical consequences of vitamin D insufficiency are less established despite expanding literature in this area. Findings from numerous epidemiological studies have linked low 25-hydroxyvitamin D [25(OH)D] concentrations to an increased risk of a myriad of adverse health consequences in both adults and children[6,7], where low concentrations in children have been linked to asthma[8,9], eczema[10], respiratory tract infections[11,12], and diabetes[13], among others.[6] Yet, the majority of randomised controlled trials do not show improved outcomes with vitamin D supplementation,[6,7,14,15] and meta-analyses are inconclusive and widely debated.[6–8,11,12,14–18]

Against this backdrop of ongoing debate, vitamin D deficiency is widely prevalent in all age groups and seen as a growing public health concern worldwide.[19] The US,[20] Canada,[21] Australia,[22,23] France,[24] and the UK[25,26] have all reported a significant increase in laboratory vitamin D testing, with as much as a 90-fold increase over a 10-year period.[22,23] In England, there has also been a marked increase in the number of vitamin D prescriptions issued by general practitioners (GPs) in primary care, with annual spending on vitamin D prescriptions increasing from £0.5 to £40 million between 2007 and 2016[27]; a rising trend that was partly attributed to vitamin D prescriptions issued for children.[28]

These reported trends have understandably led many to question the clinical appropriateness of vitamin D testing in practice.[29,30] However, even though vitamin D is also widely available without a prescription, the increasing number of GPs issued vitamin D prescriptions has surprisingly not attracted the same degree of attention. For the UK where the healthcare service is publicly-funded and free, national strategy is centred on primary prevention in managing vitamin D deficiency. Specifically, UK national guidance (A summary of UK guidelines is presented in Supplementary Table S1) has, until relatively recently, targeted recommendations on supplementation mainly towards high-risk groups.[31–

For peer review only - http://bmjopen.bmj.com/site/about/guidelines.xhtml

[40] All children under 5 years of age including breastfed infants, if their mother is also at risk of vitamin D deficiency, were recommended to take daily vitamin D supplement at prevention doses.[32–35,37,38] There were no specific recommendations for children 5 years or above unless individuals were considered at risk. In any children where 25(OH)D is tested, treatment at pharmacological doses is guided by whether 25(OH)D concentration is below 25 nmol/L, the threshold set by the UK Scientific Advisory Committee on Nutrition as vitamin D deficient.[31,39] Little is known about how well these recommendations are implemented in practice, but it has been noted that the complexity of the advice and poor communications may have caused confusion among both health professionals and the public.[37] Considering there are numerous other international and national guidelines on vitamin D supplementation and health which differ in their definition of vitamin D deficiency and supplementation recommendations[41–43], a comprehensive analysis of vitamin D prescribing by GPs offers an opportunity to facilitate the appropriate use of vitamin D supplementation for patient benefit and contain healthcare expenditure.

The aim of our study was to examine recent trends in prescribing pattern of vitamin D supplementation in children. We quantified temporal changes in the incidence of vitamin D prescriptions issued by GPs in a UK population-based study. Moreover, we examined the proportions of prescriptions by type of supplementation, dose, dosing schedule, as well as the proportions that can be linked to 25(OH)D laboratory test results and clinical symptoms suggestive of vitamin D deficiency.

Methods

Data source

We obtained electronic health records from patients registered with UK general practices contributing to The Health Improvement Network (THIN). The THIN database contains anonymised data from 744 general practices using the Vision computer system (In Practice Systems, London, UK); data are from all patients in participating practices unless individual patients opt-out of THIN. It contains data of approximately 16 million patients which has been shown to be broadly representative of the UK

For peer review only - http://bmjopen.bmj.com/site/about/guidelines.xhtml

population in terms of age, sex, prevalence of medical conditions, and mortality rates.[44] As of 2015, the THIN dataset is reported to cover 6% of the UK population.[45]

THIN contains information on diagnoses, laboratory tests, symptoms and hospital referrals, as well as basic sociodemographic information. Clinical events and diagnoses are recorded using a hierarchical coding system called Read codes.[46] THIN also includes records of all prescriptions issued and these are linked to the British National Formulary. Prescribing data are particularly well recorded in THIN since the computerised entry made by the GP is directly used to issue a prescription to the patient. Prescribing data in THIN has been shown to be comparable to data on dispensed prescriptions with a mean practice redemption rate for all prescribing of 98.5%.[47]

Study population

Study cohort selection is presented in supplementary Figure S1. We identified a cohort of children aged 0 to 17 years registered with a THIN general practice from 1st January 2008 to 31st December 2016. Individuals were not eligible to enter the study until three months after their registration with the practice in order to exclude those with prevalent prescriptions.[48] Cohort entry was defined by the latest of: the study start date (1st January 2008), 3 months after the patient's registration with the practice, or the date the practice met two predefined quality indicators (acceptable mortality recording and acceptable computer usage) for electronic data recording.[49,50] Children with cystic fibrosis, chronic renal failure, inflammatory bowel diseases or chronic liver diseases were excluded as these conditions are known to affect vitamin D absorption or metabolism and therefore require specialist management (Code lists for these conditions were created using previously published methodology[51]). Children were followed until an incident prescription of vitamin D, or censored on death, midpoint of their 18th birthday, end of registration with a practice, the last collection date of data from the practice, or end of the study period (31st December 2016), whichever occurred first. The total number of patient-years between cohort entry and exit defined the denominator for incidence calculations.

Outcome

For peer review only - http://bmjopen.bmj.com/site/about/guidelines.xhtml

A vitamin D prescription was defined as a first prescription record (incident prescription) of either: 1) a single-ingredient product containing calciferol, ergocalciferol or colecalciferol as the only active ingredient; or 2) a combination product containing calciferol, ergocalciferol or colecalciferol with other active ingredients (e.g. ergocalciferol with calcium, multi-vitamin preparations). For the latter, only those that could be linked to a Read code indicating vitamin D deficiency or insufficiency or 25(OH)D testing within 90 days either side of the date of the prescription were included. Comprehensive lists of drug codes and Read codes were developed using previously published methodology (Supplementary Table S2 and S3).[51] Incident cases were defined as children with a first ever recording of a vitamin D prescription in THIN during the study period. The date at which the first recording of vitamin D prescription was made was classified as the index date. Consultations where GPs did not issue a prescription but recommended self-purchase of over the counter vitamin D supplement were not considered as prescriptions.

Covariates

Age groups were defined as: up to 6 months, 6 months – 4 years, 5 -11, and 12-17 years. Ethnicity was grouped into the 2011 UK Census 5- category classification; children with multiple ethnicity records belonging to different categories were included in the analysis under the 'missing data' category. Townsend index, an area-based relative measure of material deprivation, was extracted; the value closest to the start of the observation period was used for patients with multiple records. For this exploratory study, a pragmatic approach was taken to handle missing data separately under the 'missing data' category.

25(OH)D laboratory test results associated with incident prescriptions were identified by temporal proximity. A result was considered to be contemporary and would have formed the basis of prescribing if it occurred 90 days prior to the index date. Based on the elimination half-life of 25(OH)D, results recorded between 91-180 days after the index date were used to assess post supplementation vitamin D status. Concentrations <25 nmol/L were categorised as deficient as per the UK Department of Health and Social Care definition relevant at the time of the study period.[31] Concentrations were further

categorised as insufficient (25-50 nmol/L) as defined by the British Paediatric and Adolescent Bone Group and supported by the British Society of Paediatric Radiology and the Royal College of Paediatrics and Child Health.[34,36,38]

Read codes for symptoms or clinical complications that could potentially be related to vitamin D deficiency within the 90 days prior to the index date were extracted and organised into 12 categories as presented in Supplementary Table S4.

Incident vitamin D prescriptions analyses

Children with multiple vitamin D prescriptions on the index date (534 patients) were excluded from prescription pattern analyses as it was not possible to determine whether all prescriptions were relevant. Prescriptions were categorised according to the type of supplementation as per case definition above. Equivalent daily dose (EDD) was calculated for each incident prescription as determined by the quantity of vitamin D [international unit (IU)] prescribed per dose and the prescribed dosing frequency. Prescriptions with titration dosages (e.g. two tablets daily for 2 weeks and then one tablet monthly) were handled by considering the first dosage only to capture the initial management strategy. Where unit of dosing was missing (e.g. every day), one single dosing unit was assumed for tablet/capsule products only. Generic (e.g. as directed), or ambiguous (e.g. once daily for a liquid product) dosages were categorised under the ‘undetermined’ category. EDDs were further categorised into dose bands. To assist interpretation, doses of 280 - 400 IU were considered prevention doses across all age groups based on recommendations relevant at the time of the study period.[31,33–35,39] Doses between 401-1000 IU were categorised as a separate prevention dose category given divergent views exist within the medical community with regards to supplementation strategies for primary prevention.[36–38] Doses between 1,001 and 10,000 IU/day were considered as pharmacological treatment doses independent of age.[36,38] Prescription quantity supplied in days was calculated based on total prescription quantity issued and corresponding dosage and capped at 365 days to take account of product shelf life. High-dose vitamin D regimens (stoss regimens) prescribed either as intramuscular injections or oral preparations were categorised separately and reported as either low dose (total dose <150,000 IU) or high dose (total

For peer review only - http://bmjopen.bmj.com/site/about/guidelines.xhtml

dose ≥150,000 IU) stoss. As oral stoss regimens may be prescribed as a single dose or spread over several days, prescriptions with explicit duration directions for ≤15 days (e.g. 5,000 IU once daily for 14 days), or where the quantity prescribed were ≤7 days with an equivalent daily dose ≥10,000 IU (e.g. 5 capsules of 20,000 IU with dosing direction “one capsules once daily”) were also categorised as stoss.

Statistical analysis

Cohort characteristics are presented by sex, ethnicity, socioeconomic quintile, country, calendar year, and year of cohort entry as frequencies (%). Medians and interquartile range (IQR) are presented for age at entry and follow-up time. We calculated crude annual incidence rates by dividing the number of children with a first ever recording of a vitamin D prescription by the Person Years at Risk (PYAR). Confidence intervals (95% CI) were calculated assuming a Poisson distribution. Using Poisson regression, we estimated sex stratified incidence rate ratios with 95% confidence intervals, adjusting for potential confounders. Time period was fitted as both a linear and categorical variable and models compared using the log likelihood ratio test; there was evidence that the categorised time period was a better fit with the data and thus the time period was treated as a categorical variable. Age was also fitted as a categorial variable as it had a non-linear relationship. A fully adjusted sex stratified model with an interaction between age and ethnicity was fitted and examined; while the interaction term was statistically significant, it did not change the estimates of the other variables and the incidence rates predicted by the two models were similar. However, as the model with the interaction term resulted in unstable predictions, the interaction term was not included in the final model. The final model with the inclusion of the GP practice as a random effect to account for any data clustering was used to calculate adjusted incidence rate ratios (IRRs). Proportions of prescriptions by type of supplementation, dose, dosing schedule, linked to 25(OH)D laboratory test concentrations and clinical symptoms suggestive of vitamin D deficiency, are presented as frequencies (%). Trends in prescribing characteristics were quantified using Spearman correlation coefficients using all years between 2008 and 2016. Data analysis was conducted using Stata 15 (Stata Corp, College Station, Texas).

For peer review only - http://bmjopen.bmj.com/site/about/guidelines.xhtml

Three sensitivity analyses were performed. Firstly, we considered a narrower case definition for vitamin D prescription, where only single-ingredient products were included. Secondly, we repeated the incidence analysis using complete cases only, that is among children who had complete data on sex, age, ethnicity, socioeconomic status, country and calendar year. Finally, we considered a broader definition for prevention doses, where equivalent daily doses of ≤1,000 IU were included.

Patient and public involvement

Patient and parent/carer representatives were involved in developing study plans and approving the lay summaries for the grant supporting this study. Patients were not involved in setting the specific research question, the outcome measure, the design, or implementation of this study. No patients were asked to advise on interpretation or writing up of results. It is not possible to disseminate the results of the research to study participants, but views from patient and parent/carer representatives will be sought in disseminating the research findings.

Ethics

The THIN data collection scheme to obtain and provide anonymised patient data was approved by the National Health Service South-East Multicentre Research Ethics Committee (REC Reference: 04/MRE01/9) in 2002. The study protocol was approved by an independent Scientific Review Committee (Reference: 16THIN035).

Results

A total of 2,051,403 eligible children from 723 general practices contributed data between Jan 1, 2008 and Dec 31, 2016, giving a total of 8 million person-years of follow-up. Of those children, 12,277 had an incident vitamin D prescription during the study period. Patient characteristics stratified by sex, ethnicity, country of general practice, and socioeconomic status are shown in Table 1 for the overall cohort and the incident cohort.

Crude incidence

For peer review only - http://bmjopen.bmj.com/site/about/guidelines.xhtml

The crude annual incidence of vitamin D prescribing in children increased by 26-fold between 2008 and 2016 rising from 10.8 (95% CI: 8.9, 13.1) to 276.8 (95% CI: 264.3, 289.9) per 100,000 person-years. Figure 1A shows temporal trends in vitamin D prescribing in children alongside key national guidelines or reports on vitamin D deficiency.[31–40,52] Prescribing rates for females were higher than males, with an average ratio of male to female prescribing rate of 1:1.5 across all time periods. As can be seen from Figure 1B, increasing prescribing trend (rate per 100,000 person-years) was particularly apparent in children aged 12 - 17 years [14.8 (95% CI: 11.1, 19.8) to 529.5 (95% CI: 499.2, 561.7)], followed by those aged 5 - 11 years [7.8 (95% CI: 5.4, 11.2) to 188.3 (95% CIL 172.5, 205.5)], 6 months - 4 years [10.2 (95% CI: 6.9, 15.1) to 111.6 (95% CI: 96.8, 128.7)], and less than 6 months [10.6 (95% CI: 1.5, 75.2) to 79 (95% CI: 32.9, 189.7)]. When vitamin D prescription was defined using single-ingredient vitamin D products only, we found similar trends to the main analysis (Figure 1A).

Adjusted incidence rates

In multivariable analysis, increasing age, non-white ethnicity, and social deprivation were associated with higher incidence rates of vitamin D prescribing (Table 2). Among children aged 12-17 years, sex difference in incidence rate ratios was observed, with 1.8-fold higher vitamin D supplementation prescribing in females than males. General practices in England were also associated with higher incidence rate ratios as compared to Wales, Scotland and Northern Ireland. For both males and females, adjusted incidence rate ratios were higher than their corresponding crude values, with a 32-fold and 39-fold increase in the incidence of vitamin D prescribing between 2008 and 2016, respectively. A sensitivity analysis using complete cases only was comparable to the main analysis, albeit the magnitude of the effects of ethnicity was marginally greater for both sexes, along with a smaller temporal difference in annual incidence rates (Supplementary Table S5).

25-hydroxyvitamin D testing and presence of symptoms at initiation of supplementation

25(OH)D concentrations recorded before starting vitamin D supplementation are presented in Figure 2A. Overall, 28.7 to 56.4% of prescriptions annually had no linked 25(OH)D results recorded in the 3 months prior to the index date. The proportion of children with laboratory confirmed vitamin D

For peer review only

deficiency [25(OH)D <25 nmol/L] increased from 25.7 to 40.9% between 2008 and 2011 before showing a gradual decrease to 27.2% in 2016, coinciding with the issue of public health guidelines on primary prevention of vitamin D deficiency for at risk individuals. Over the study period, increased prescribing of vitamin D supplementation was observed in children with insufficient status [25(OH)D between 25-50 nmol/L], increasing from 15.8 - 21.7% between 2008 and 2011 to 27.9 - 37.1% during the period between 2012 to 2016.

29.5% of children had symptoms related to vitamin D deficiency (Supplementary Table S5). The most commonly recorded symptoms were those related to musculoskeletal or non-specific pain, tiredness or fatigue. In the group of children with no recorded pre-supplementation 25(OH)D results, only 15.4% had recorded symptoms.

Types of supplementation

Overall, 109 different pharmaceutical preparations of at least 32 dosage strengths (e.g. 3,000 IU/mL, 20,000 IU/tablet) were prescribed. A temporal change in supplementation form was observed; colecalciferol products accounted for 9.9% of the prescriptions in 2010 and increased to 90.2% in 2016, explaining the trends in prescribing patterns shown in Figure 1 (Supplementary Figure S2).

Doses and frequencies of administration

The EDD prescribed ranged from 27 to 160,000 IU/day (prescribed doses by age group is summarised in Supplementary Table S6) with a median duration of supply of 56 days (IQR: 30, 83 days). There were 237 (2.0%) prescriptions with doses less than the lowest recommended primary prevention dose (<280 IU/day), and 132 (1.1%) prescriptions with doses above the highest recommended pharmacological dose (>10,000 IU/day). A total of 24 different dosing frequencies were noted, with 37.2% of children on regimens other than once daily dosing (Supplementary Table S7).

Trend analysis of supplementation regimens stratified by pre-supplementation 25(OH)D concentrations is presented in Figure 3. Among children with deficiency [25(OH)D <25 nmol/L], prescriptions with

For peer review only - http://bmjopen.bmj.com/site/about/guidelines.xhtml

EDD >1,000 IU/day, considered as pharmacological doses, showed a yearly increased from 3.8% in 2008 to 79.4% in 2016 (spearman's rho = 0.983, p<0.001), which is consistent with UK recommendations. Increased used of pharmacological doses (0% in 2008 to 53% in 2016; spearman's rho = 1, p<0.001) was also noted among children with 25(OH)D concentrations between 25-50 nmol/L who, according to UK national guidance, should have been recommended supplement at prevention doses; the corresponding temporal decrease of prescriptions at prevention doses was noted irrespective of the cut-off value used to define prevention doses (400 or ≤1,000 IU). A smaller but increased trend was similarly noted in the group of children with no linked 25(OH)D concentrations (35.7% in 2008 to 49.6% in 2016; spearman's rho = 0.7333, p = 0.025). Overall, vitamin D supplementation at pharmacological doses (>1,000 IU/day or stoss) was prescribed to 42.8% of children with insufficient status, outside of UK recommendations.

25-hydroxyvitamin D testing at follow-up

In the first 90 days post initiation of vitamin D supplementation, 9% (n=1,109) of children had 25(OH)D concentrations recorded, of which 24.9% were recorded within 1 month after the index date, outside of guideline recommendations. Post-supplementation testing at the recommended time intervals was low; 25(OH)D results in the period between 91 to 180 days after incident prescriptions were available for only 8.4% (n=1,035) of children (Figure 2B). Over the study period, the proportion of children with post-supplementation 25(OH)D concentrations >50 nmol/L increased from 11.1% to 76.3%.

Discussion

In a large cohort representative of children in UK primary care, there has been a marked temporal increase in vitamin D prescribing, even after adjustment for changes in population demographics. The adjusted incidence rates increased by 32-fold and 39-fold between 2008 and 2016 for males and females, respectively. 25(OH)D concentrations for guiding supplementation were not available in over 30% of children annually, and an increasing trend in prescribing vitamin D supplementation at pharmacological doses irrespective of 25(OH)D concentrations was noted with wide variations in supplementation regimens prescribed, deviating in part from UK recommendations. Furthermore, with

For peer review only - http://bmjopen.bmj.com/site/about/guidelines.xhtml

no clear evidence to support the use of vitamin D supplementation on health outcomes, other than for the prevention or treatment of rickets and osteomalacia, this aberrant prescribing practice incurs a huge, potentially avoidable, expense to our health care system.

This is the first study to report UK estimates of incidence rates of vitamin D prescribing in children using a comprehensive case definition for vitamin D prescription. An earlier study based in England included only 156 general practices and reported a marked increase in the diagnosis of vitamin D deficiency and prescription costs of single-ingredient vitamin D products in children between 2000 and 2014.[28,53] Our study reports similar trends but provides data from a much larger number of practices across the whole of the UK, with detailed socio-demographic prescribing patterns and extending the data to 2016.

Vitamin D prescribing in children increased sharply between 2011 and 2014, coinciding with the publication of guidelines and reports from the UK government and national guideline committees that emphasise the importance of vitamin D supplementation in certain groups of vulnerable children and young people.[31–40] These have, in part, raised awareness of vitamin D deficiency among primary care practitioners, and explain the increase in vitamin D prescribing practices. Notably, the increase prescribing rates over time can be attributable primarily to children 5 years or older, suggesting widening awareness of vitamin D deficiency, with risk perceptions among GPs going beyond children considered as high risk. The observed change in the type of supplementation prescribed, from ergocalciferol and combination products to colecalciferol products, is congruent with increase product availability of the latter[27] and may potentially also relate to data suggesting colecalciferol is more efficacious at raising serum 25(OH)D concentrations than ergocalciferol.[54]

Patient age, sex and ethnicity were identified as drivers for prescribing, in keeping with the higher prevalence rates of vitamin D deficiency reported in teenage girls, and non-white ethnic groups in the UK.[39] Interestingly, we also observed increased rates of prescribing among practices in England as compared to those in Wales (IRR = 0.3), Scotland (IRR = 0.2) and Northern Ireland (IRR = 0.2),

For peer review only - http://bmjopen.bmj.com/site/about/guidelines.xhtml

suggesting other contextual factors influencing practitioners' prescribing behaviour; disparities may be due to variations in regional strategies in implementing national guidelines, or differences in practitioners' experience and attitudes towards vitamin D deficiency, and require further exploration.[55–57]

The initiation of vitamin D supplementation should be guided by 25(OH)D concentrations, except when used for primary prevention, as indicated in UK guideline recommendations.[36,38] Yet, our results show an absence of 25(OH)D results in over 30% of prescriptions annually since 2009, a practice also reported by others.[56] It is possible that a proportion of children may have 25(OH)D concentrations checked and recorded by hospital clinicians; although children with conditions known to affect vitamin D absorption and metabolism and thus likely to receive secondary input were already excluded in our analysis. We also recognised that a proportion of these prescriptions may have been intended for primary prevention. However, applying the most conservative primary prevention dose definition of ≤1,000 IU/day, our finding that at least 40% of prescriptions exceeded 1,000/day, and together with only a small number of children with recorded symptoms possibly related to vitamin D deficiency, would be suggestive of aberrant prescribing.

Our analysis also found an upward trend in prescribing of vitamin D supplementation at pharmacological doses independent of 25(OH)D concentrations. To some extent, this pattern observed for children with deficiency suggests that practitioners are responsive to prescribing guidelines[36,38], at least on a population level. On the other hand, a comparable increase was also seen in children with vitamin D insufficiency; this deviates from UK national recommendations, and perhaps reflects confusion arising from multiple, sometimes conflicting, recommendations. At the same time, these deviations from recommendations might suggest a somewhat less cautious attitude towards the use of higher than recommended doses of vitamin D among practitioners. Vitamin D has a wide therapeutic window and risk of toxicity is low, nevertheless the potential risk of vitamin D toxicity exists and requires further exploration given its widespread, and sometimes unregulated use.

For peer review only - http://bmjopen.bmj.com/site/about/guidelines.xhtml

The major strength of our study is the large sample size that is representative of real-life clinical practice across the UK. Prescribing details in THIN are well recorded because all prescriptions from general practice are generated via a standardised computerised system. The inclusion of combination vitamin D products in the case definition also captures real life clinical practice, but some cases could be missed if combination vitamin D product was prescribed but a diagnosis of vitamin D status was not entered in the primary care record. There are some limitations to our study too. The first vitamin D prescription recorded in THIN may not be the first ever prescription issued to the patient as initial prescribing may have occurred in secondary care and continued in general practice. However, most prescriptions issued by UK hospitals are limited to 7-14 days for clinically urgent conditions only, so the analysed sample is very likely to be representative of true practice. The study design did not allow us to determine the practitioners' rationale for prescribing, nor could we assess clinical appropriateness of prescriptions on an individual level. Trends in seasonal variations in prescribing was also not explored. Despite these limitations, our study explored the major clinical indicator for vitamin D supplementation, namely 25(OH)D concentrations and presence of symptoms.

Our study highlights two key implications for practice. Firstly, diverse clinical practice highlights the need for a broader understanding of the multitude factors influencing GPs' prescribing decisions for vitamin D supplementation in order to reduce inappropriate prescribing.[55,57] Further guidelines alone, even in the absence of ambiguity, are unlikely to be effective. Secondly, vitamin D supplementation exceeding recommended doses, and arguably, prescriptions at prevention doses may not be the best use of the limited health resources. On this basis, at least 32.3% of our prescriptions would fall into this category. Using the most conservative estimate from an earlier study[28] (£1.65 million for the total direct cost of vitamin D prescriptions in children per year), and without costing for consultation time with health care professionals, dispensing or tests, we estimate an annual saving of £0.5 million.

Conclusion

There has been a marked and sustained increase in vitamin D prescribing in children in UK primary care. While this may reflect the success of the Department of Health and Social Care's efforts in raising

For peer review only - http://bmjopen.bmj.com/site/about/guidelines.xhtml

awareness of vitamin D deficiency, findings from our study would suggest that nationally set recommendations on vitamin D supplementation are not consistently followed by GPs, in terms of the number of patients treated, the doses used for supplementation, as well as the practice of prescribing vitamin D without appropriate testing. More recent NHS England guidance[30], that recommends prescribing pharmacological doses of vitamin D supplementation only to those diagnosed with vitamin D deficiency, has the potential to reduce unnecessary prescriptions and UK healthcare expenditure, but its impact on prescribing practice would require further evaluation as previous guidelines have not always translated into practice.

For peer review only

For peer review only - http://bmjopen.bmj.com/site/about/guidelines.xhtml

Contributors

MW, GR, RS and JP were involved in the conception of the study and obtaining funding. MW, LH, EB, JP, RS and GR contributed to the study protocol and interpretation of data. MW had full access to the database, programming code and performed the data extraction and analysis. MW drafted the manuscript which LH, EB, JP, RS and GR contributed to and revised critically before approval of the final manuscript. MW is the guarantor. The corresponding author attests that all listed authors meet authorship criteria and that no others meeting the criteria have been omitted.

Funding

This study was funded by a Clinical Doctoral Research Fellowship grant (ICA-CDRF-2016-02-057) from the UK National Institute for Health Research (NIHR). MW is a doctoral student supported by this grant. Independent expert peer reviewers provided feedback on the grant application underpinning this study but had no further role in study design, data collection, analysis, interpretation, or drafting of the manuscript. LH is fully funded by the Wellcome Trust (209207/Z/17/Z). RS holds a Career Development Fellowship with the National Institute for Health Research. A part of the work took place in the Biomedical Research Centre at Great Ormond Street Hospital for Children NHS Foundation Trust and University College London. The views expressed are those of the authors and not necessarily those of the NHS, the NIHR, or the Department of Health and Social Care.

Competing interests

All authors have completed the ICMJE uniform disclosure form at www.icmje.org/coi_disclosure.pdf and declare: no support from any organisation for the submitted work; no financial relationships with any organisations that might have an interest in the submitted worked in the previous three years; no other relationships or activities that could appear to have influenced the submitted work

Data sharing

The General Practices in The Health Improvement Network (THIN) is run by the Department of Health and Social Care. All the data are available via an application to THIN. Data acquisition is associated with a fee. Code lists used in this study will be available on request.

For peer review only

References

1 Boucher BJ. Causal link between vitamin D deficiency and ill health still possible. *BMJ* 2014; **348**: g2923.

2 Welsh P, Sattar N. Vitamin D and chronic disease prevention. *BMJ* 2014; **348**: g2280.

3 Stokes CS, Lammert F. Vitamin D supplementation: less controversy, more guidance needed. *F1000Res* 2016; **5(F1000 Faculty Rev):2017**.

4 Bischoff-Ferrari HA, Orav EJ, Abderhalden L, *et al*. Vitamin D supplementation and musculoskeletal health. *The Lancet Diabetes & Endocrinology* 2019; **7**: 85.

5 Bolland MJ, Grey A, Avenell A. Vitamin D supplementation and musculoskeletal health – Authors' reply. *The Lancet Diabetes & Endocrinology* 2019; **7**: 88–89.

6 Theodoratou E, Tzoulaki I, Zgaga L, *et a*l. Vitamin D and multiple health outcomes: umbrella review of systematic reviews and meta-analyses of observational studies and randomised trials. *BMJ* 2014; **348**: g2035.

7 Autier P, Mullie P, Macacu A, *et al.* Effect of vitamin D supplementation on non-skeletal disorders: a systematic review of meta-analyses and randomised trials. *The Lancet Diabetes & Endocrinology* 2017; **5**: 986–1004.

8 Jolliffe DA, Greenberg L, Hooper RL, *et al.* Vitamin D supplementation to prevent asthma exacerbations: a systematic review and meta-analysis of individual participant data. *The Lancet Respiratory Medicine* 2017; **5**: 881–890.

9 Martineau AR, Cates CJ, Urashima M, *et al.* Vitamin D for the management of asthma. *Cochrane Database of Systematic Reviews* 2016.

10 Huang CM, Lara-Corrales I, Pope E. Effects of Vitamin D levels and supplementation on atopic dermatitis: A systematic review. *Pediatr Dermatol* 2018; **35**: 754–760.

11 Jolliffe DA, Griffiths CJ, Martineau AR. Vitamin D in the prevention of acute respiratory infection: Systematic review of clinical studies. *The Journal of Steroid Biochemistry and Molecular Biology* 2013; **136**: 321–329.

12 Martineau AR, Jolliffe DA, Hooper RL, *et al.* Vitamin D supplementation to prevent acute respiratory tract infections: systematic review and meta-analysis of individual participant data. *BMJ* 2017; **356**: i6583.

13 Savastio S, Cadario F, Genoni G, *et al.* Vitamin D Deficiency and Glycemic Status in Children and Adolescents with Type 1 Diabetes Mellitus. *PLOS ONE* 2016; **11**: e0162554.

14 Winzenberg TM, Powell S, Shaw KA, *et al*. Vitamin D supplementation for improving bone mineral density in children. *Cochrane Database of Systematic Reviews* 2010.

15 Bolland MJ, Grey A, Avenell A. Effects of vitamin D supplementation on musculoskeletal health: a systematic review, meta-analysis, and trial sequential analysis. *The Lancet Diabetes & Endocrinology* 2018; **6**: 847-858.

16 Bischoff-Ferrari HA, Willett WC, Wong JB, *et al*. Fracture prevention with vitamin D supplementation: a meta-analysis of randomized controlled trials. *JAMA* 2005; **293**: 2257–2264.

17 World Health Organisation. Vitamin D for prevention of respiratory tract infections. WHO. http://www.who.int/elena/titles/commentary/vitamind_pneumonia_children/en/ (accessed Feb 28 2019).

18 Martineau A. Vitamin D supplementation to prevent asthma exacerbations – Authors' reply. *The Lancet Respiratory Medicine* 2018; **6**: e26–e27.

For peer review only - http://bmjopen.bmj.com/site/about/guidelines.xhtml

19 Palacios C, Gonzalez L. Is vitamin D deficiency a major global public health problem? *J Steroid Biochem Mol Biol* 2014; **144PA**: 138–145.

20 Shahangian S, Alspach TD, Astles JR, *et al*. Trends in laboratory test volumes for Medicare Part B reimbursements, 2000-2010. *Arch Pathol Lab Med* 2014; **138**: 189–203.

21 Ontario Health Technology Advisory Committee. Clinical Utility of Vitamin D Testing. *Ont Health Technol Assess Ser* 2010; **10**: 1–93.

22 Bilinski KL, Boyages SC. The rising cost of vitamin D testing in Australia: time to establish guidelines for testing. *The Medical Journal of Australia* 2012; **197**: 90.

23 Bilinski K, Boyages S. Evidence of overtesting for vitamin D in Australia: an analysis of 4.5 years of Medicare Benefits Schedule (MBS) data. *BMJ Open* 2013; **3**: e002955.

24 Caillet P, Goyer-Joos A, Viprey M, *et al*. Increase of vitamin D assays prescriptions and associated factors: a population-based cohort study. *Scientific Reports* 2017; **7**: 10361.

25 Sattar N, Welsh P, Panarelli M, *et al*. Increasing requests for vitamin D measurement: costly, confusing, and without credibility. *The Lancet* 2012; **379**: 95–96.

26 Zhao S, Gardner K, Taylor W, *et al*. Vitamin D assessment in primary care: changing patterns of testing. *London J Prim Care (Abingdon)* 2015; **7**: 15–22.

27 NHS Digital. Prescription Cost Analysis. NHS Digital. https://digital.nhs.uk/data-and-information/publications/statistical/prescription-cost-analysis (accessed Feb 28 2019).

28 Basatemur E, Hunter R, Horsfall L, *et al*. Costs of vitamin D testing and prescribing among children in primary care. *Eur J Pediatr* 2017; **176**: 1405–1409.

29 Boyages SC. Vitamin D testing: new targeted guidelines stem the overtesting tide. *Med J Aust* 2016; **204**: 18.

30 NHS England. Conditions for which over the counter items should not routinely be prescribed in primary care: Guidance for CCGs. 2018. https://www.england.nhs.uk/wp-content/uploads/2018/03/otc-guidance-for-ccgs.pdf (accessed Feb 28 2019).

31 Scientific Advisory Committee on Nutrition. Update on Vitamin D - Position statement by the Scientific Advisory Committee on Nutrition. 2007. https://assets.publishing.service.gov.uk/government/uploads/system/uploads/attachment_data/file/339349/SACN_Update_on_Vitamin_D_2007.pdf (accessed Feb 28 2019).

32 National Institute for Health and Care Excellence. Improving the nutrition of pregnant and breastfeeding mothers and children in low-income households. (PH11). 2008. https://webarchive.nationalarchives.gov.uk/20080912094204/http://www.gserve.nice.org.uk/nicemedia/pdf/PH011guidance.pdf (accessed Feb 28 2019).

33 UK Chief Medical Officers. Vitamin D - advice on supplements for at risk groups - letter from UK Chief Medical Officers. 2012. https://www.gov.uk/government/publications/vitamin-d-advice-on-supplements-for-at-risk-groups (accessed Feb 28 2019).

34 Arundel P, Ahmed SF, Allgrove J, *et al.* British Paediatric and Adolescent Bone Group's position statement on vitamin D deficiency. *BMJ* 2012; **345**: e8182.

35 Royal College of Paediatrics and Child Health. Vitamin D: position statement. 2012. https://www.rcpch.ac.uk/resources/vitamin-d-position-statement (accessed Feb 28 2019).

36 Royal College of Paediatrics and Child Health. Guide for Vitamin D in Childhood. 2013. https://www.rcpch.ac.uk/sites/default/files/2018-03/guide_to_vitamin_d_in_childhood.pdf (accessed Feb 28 2019).

For peer review only

37 National Institute for Health and Care Excellence. Vitamin D: supplement use in specific population groups (PH56). 2014. https://www.nice.org.uk/guidance/ph56/resources/vitamin-d-supplement-use-in-specific-population-groups-pdf-1996421765317 (accessed Feb 28 2019).

38 National Osteoporosis Society. Vitamin D and Bone Health: A Practical Clinical Guideline for Management in Children and Young People. 2015. https://nos.org.uk/media/2074/vitamin-d-and-bone-health-children.pdf (accessed Feb 28 2019).

39 Scientific Advisory Committee on Nutrition. SACN vitamin D and health report. 2016.

40 National Institute for Health and Care Excellence Clinical Knowledge Summaries. Vitamin D deficiency in children. 2016. https://cks.nice.org.uk/vitamin-d-deficiency-in-children#!references (accessed Feb 28 2019).

41 Rosen CJ, Abrams SA, Aloia JF, *et al.* IOM Committee Members Respond to Endocrine Society Vitamin D Guideline. *J Clin Endocrinol Metab* 2012; **97**: 1146–1152.

42 Munns CF, Shaw N, Kiely M, *et al.* Global Consensus Recommendations on Prevention and Management of Nutritional Rickets. *J Clin Endocrinol Metab* 2016; **101**: 394–415.

43 Institute of Medicine (US) Committee to Review Dietary Reference Intakes for Vitamin D and Calcium. *Dietary Reference Intakes for Calcium and Vitamin D*. National Academies Press (US): Washington (DC), 2011.

44 Blak B, Thompson M, Dattani H, *et al*. Generalisability of The Health Improvement Network (THIN) database: demographics, chronic disease prevalence and mortality rates. *Journal of Innovation in Health Informatics* 2011; **19**: 251–255.

45 IMS Health. THIN Data Guide for Researchers. 2015.

46 Chisholm J. The Read clinical classification. *BMJ* 1990; **300**: 1092.

47 NHS Digital. Prescribing Compliance, A review of the proportion of prescriptions dispensed. 2011. https://digital.nhs.uk/data-and-information/publications/statistical/prescribing-compliance/prescribing-compliance-a-review-of-the-proportion-of-prescriptions-dispensed (accessed Feb 28 2019).

48 Lewis JD, Bilker WB, Weinstein RB, *et al*. The relationship between time since registration and measured incidence rates in the General Practice Research Database. *Pharmacoepidem Drug Safe* 2005; **14**: 443–451.

49 Maguire A, Blak BT, Thompson M. The importance of defining periods of complete mortality reporting for research using automated data from primary care. *Pharmacoepidem Drug Safe* 2009; **18**: 76–83.

50 Horsfall L, Walters K, Petersen I. Identifying periods of acceptable computer usage in primary care research databases. *Pharmacoepidemiol Drug Saf* 2013; **22**: 64–69.

51 Davé S, Petersen I. Creating medical and drug code lists to identify cases in primary care databases. *Pharmacoepidem Drug Safe* 2009; **18**: 704–707.

52 Department of Health & Food Standards Agency, Department of Health & Food Standards Agency. National Diet and Nutrition Survey. Headline results from Years 1 and 2 (combined) of the Rolling Programme (2008/2009 – 2009/10). 2011. https://www.gov.uk/government/publications/national-diet-and-nutrition-survey-headline-results-from-years-1-and-2-combined-of-the-rolling-programme-2008-9-2009-10 (accessed Feb 28 2019).

53 Basatemur E, Horsfall L, Marston L, *et al*. Trends in the Diagnosis of Vitamin D Deficiency. *Pediatrics* 2017; **139**: e20162748.

54 Tripkovic L, Lambert H, Hart K, *et al.* Comparison of vitamin D2 and vitamin D3 supplementation in raising serum 25-hydroxyvitamin D status: a systematic review and meta-analysis123. *Am J Clin Nutr* 2012; **95**: 1357–1364.

For peer review only

55 Epling JW, Mader EM, Roseamelia CA, *et al*. Emerging Practice Concerning Vitamin D in Primary Care. *Qual Health Res* 2015; **25**: 1005–1012.

56 DelGiudice NJ, Street N, Torchia RJ, *et al*. Vitamin D Prescribing Practices in Primary Care Pediatrics: Underpinnings From the Health Belief Model and Use of Web-Based Delphi Technique for Instrument Validity. *Journal of Pediatric Health Care* 2018; **32**: 536–547.

57 Rockwell M, Kraak V, Hulver M, *et al*. Clinical Management of Low Vitamin D: A Scoping Review of Physicians' Practices. *Nutrients* 2018; **10**: 493.

For peer review only

Table 1: Descriptive characteristics of the overall study cohort and incident cohort

	**Overall cohort (n=2,051,403)**	**Incident cohort (n=12,277)**
**Median age at entry, years (IQR)**	5.5 (0.8 – 11.5)	7.6 (3.5 – 10.5)
**Follow up time, years (IQR)**	3.5 (1.5 – 6.3)	4.2 (2 – 6.1)
**Sex, n (%)**		
Male	1,055,395 (51.5)	4,936 (40.2)
Female	996,008 (48·6)	7,341 (59·8)
**Ethnicity, n (%)**		
White	878,360 (42.8)	2,836 (23.1)
Asian/Asian British	76,840 (3.8)	2,928 (23.9)
Black/African/Caribbean/Black British	47,206 (2.3)	1,275 (10.4)
Other ethnic group	17,656 (0.9)	312 (2.5)
Mixed/Multiple ethnic group	28,605 (1.4)	295 (2.4)
Missing	1,002,736 (48.9)	4,631 (37.7)
**Country of GP practice, n (%)**		
England	1,451,923 (70.8)	10,921 (88.9)
Northern Ireland	84,171 (4.1)	177 (1.4)
Scotland	310,142 (15.1)	597 (4.9)
Wales	205,167 (10)	582 (4.7)
**Townsend deprivation index quintile, n (%)**		
1 (least deprived)	397,057 (19.4)	1,452 (11.8)
2	357,567 (17.4)	1,377 (11.2)
3	396,234 (19.3)	2,128 (17.3)
4	379,485 (18.5)	2,740 (22.3)
5 (most deprived)	291,502 (14.2)	2,866 (23.3)
Missing	229,558 (11.2)	1,714 (14)
**Year of cohort entry, n (%)**		
2008	1,060,098 (51.7%)	6,831 (55.6%)
2009	130,610 (6.4%)	954 (7.8%)
2010	126,825 (6.2%)	961 (7.8%)
2011	160,853 (7.8%)	1,020 (8.3%)
2012	134,390 (6.6%)	870 (7.1%)
2013	139,113 (6.8%)	757 (6.2%)
2014	119,354 (5.8%)	503 (4.1%)
2015	97,198 (4.7%)	279 (2.3%)
2016	82,962 (4.0%)	102 (0.8%)

IQR = Interquartile range; Children with no recorded data on ethnicity or Townsend index were categorised as ‘missing’.

For peer review only

Table 2: Multivariable Poisson regression models of incidence of vitamin D supplementation prescribing in children stratified by sex.

	Male	Female
	**IRR[a] (95% CI)**	**IRR[a] (95% CI)**
**Age group**		
Up to 6 months	1.00 (Reference)	1.00 (Reference)
6 months - 4 years	1.93 (1.21 – 3.07)	1.28 (0.84 – 1.96)
5 - 11 years	2.47 (1.55 – 3.92)	2.25 (1.48 – 3.42)
12 - 17 years	4.15 (2.61 – 6.60)	7.41 (4.87 – 11.27)
**Ethnicity**		
White	1.00 (Reference)	1.00 (Reference)
Asian	5.05 (4.60 – 5.54)	4.46 (4.12 – 4.81)
Black	2.83 (2.53 – 3.18)	2.83 (2.58 – 3.10)
Others	2.68 (2.20 – 3.26)	2.72 (2.33 – 3.19)
Mixed	1.93 (1.59 – 2.33)	1.84 (1.57 – 2.16)
Missing	1.48 (1.37 – 1.61)	1.42 (1.33 – 1.52)
**Townsend index**		
1 (least deprived)	1.00 (Reference)	1.00 (Reference)
2	1.03 (0.92 – 1.17)	1.03 (0.94 – 1.14)
3	1.18 (1.06 – 1.33)	1.20 (1.09 – 1.32)
4	1.37 (1.22 – 1.53)	1.37 (1.25 – 1.51)
5 (most deprived)	1.41 (1.25 – 1.59)	1.44 (1.31 – 1.59)
Missing	1.38 (1.19 – 1.60)	1.43 (1.26 – 1.62)
**Country**		
England	1.00 (Reference)	1.00 (Reference)
Wales	0.34 (0.25 – 0.46)	0.33 (0.25 – 0.46)
Scotland	0.24 (0.19 – 0.32)	0.18 (0.14 – 0.23)
Northern Ireland	0.24 (0.16 – 0.37)	0.17 (0.11 – 0.27)
**Year**		
2008	1.00 (Reference)	1.00 (Reference)
2009	2.47 (1.73 – 3.51)	2.61(1.93 – 3.52)
2010	3.68 (2.63 – 5.15)	4.97 (3.75 – 6.58)
2011	6.72 (4.88 – 9.25)	8.04 (6.12 – 10.56)
2012	14.60 (10.72 – 19.90)	16.16 (12.39 – 2.08)
2013	21.10 (15.52 – 28.67)	22.82 (17.53 – 29.72)
2014	25.81 (18.99 – 35.09)	28.14 (21.60 – 36.65)
2015	29.19 (21.44 – 39.76)	33.53 (25.70 – 43.74)
2016	32.27 (23.64 – 44.05)	38.87 (29.75 – 50.80)

Abbreviations: CI, confidence interval; IRR = incidence rate ratio.
[a]Adjusted for all variables listed in the table. The multilevel models included the general practice as a random effect.

For peer review only - http://bmjopen.bmj.com/site/about/guidelines.xhtml

Figures

Figure 1: A) Time trends in vitamin D supplementation prescribing in children using all components of the case definition together and each source of case identification independently. **B)** Time trends in vitamin D supplementation prescribing in children by different age groups. Crude incidence rates with 95% confidence intervals represented by vertical bars.

Key: SACN = Scientific Advisory Committee on Nutrition; NICE = The National Institute for Health and Care Excellence; RCPCH = Royal College of Paediatrics and Child Health; NOS = National Osteoporosis Society

1) 2007: SACN: Update on Vitamin D[31]
2) 2008: NICE: Improving the nutrition of pregnant and breastfeeding mothers and children in low-income households[32]
3) 2011: The National Diet and Nutrition Survey (NDNS) rolling programme: Data from years 1 & 2[52]
4) 2012: Department of Health: Vitamin D - advice on supplements for at risk groups[33]
5) 2012: British Paediatric and Adolescent Bone Group's position statement on vitamin D deficiency[34]
6) 2012: RCPCH: Vitamin D: Position statement[35]
7) 2013: RCPCH: Guide for Vitamin D in Childhood[36]
8) 2014: NICE: Vitamin D: increasing supplement use in at-risk groups[37]
9) 2015: NOS: Vitamin D and Bone Health[38]
10) 2016: SACN: vitamin D and health report[39]
11) 2016: NICE Clinical Knowledge Summaries: Vitamin D deficiency in children[40]

Figure 2: A) 25-hydroxyvitamin D concentrations recorded in the 90 days prior to or on the index date (n=12,277); **B)** 25-hydroxyvitamon D concentrations recorded between 91-180 days after index date (only children with known levels, n=1,035). Records with ambiguous unit of measurements were categorised as 'undetermined'.

Figure 3: Dosages of incident vitamin D prescriptions presented in equivalent daily dose among children with **A)** no pre-supplementation 25(OH)D concentrations, **B)** 25(OH)D concentrations less than 25 nmol/L, and **C)** 25(OH)D concentrations between 25 - 50 nmol/L in the 90 days prior to their incident prescriptions.

For peer review only - http://bmjopen.bmj.com/site/about/guidelines.xhtml

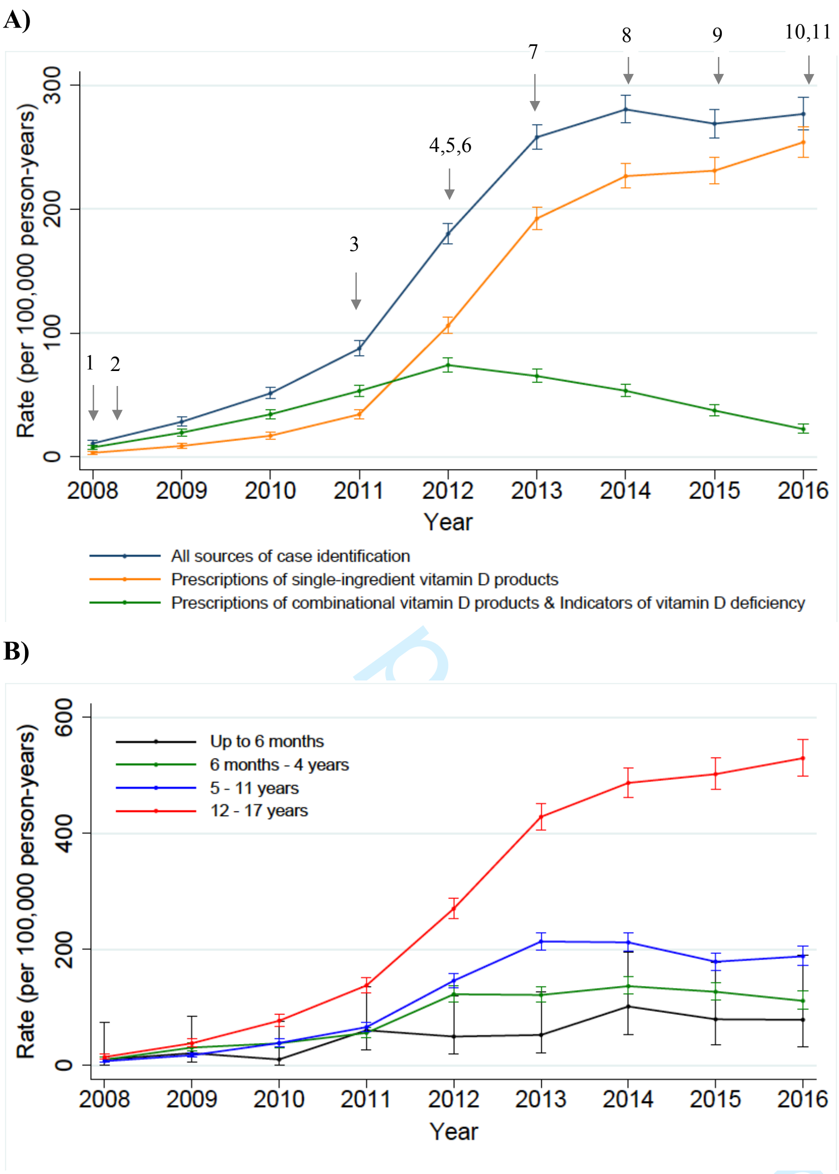

Figure 1: A) Time trends in vitamin D supplementation prescribing in children using all components of the case definition together and each source of case identification independently. **B)** Time trends in vitamin D supplementation prescribing in children by different age groups. Crude incidence rates with 95% confidence intervals represented by vertical bars.

Key: SACN = Scientific Advisory Committee on Nutrition; NICE = The National Institute for Health and Care Excellence; RCPCH = Royal College of Paediatrics and Child Health; NOS = National Osteoporosis Society

1) 2007: SACN: Update on Vitamin D[31]
2) 2008: NICE: Improving the nutrition of pregnant and breastfeeding mothers and children in low-income households[32]
3) 2011: The National Diet and Nutrition Survey (NDNS) rolling programme: Data from years 1 & 2[52]
4) 2012: Department of Health: Vitamin D - advice on supplements for at risk groups[33]
5) 2012: British Paediatric and Adolescent Bone Group's position statement on vitamin D deficiency[34]
6) 2012: RCPCH: Vitamin D: Position statement[35]
7) 2013: RCPCH: Guide for Vitamin D in Childhood[36]
8) 2014: NICE: Vitamin D: increasing supplement use in at-risk groups[37]
9) 2015: NOS: Vitamin D and Bone Health[38]
10) 2016: SACN: vitamin D and health report[39]
11) 2016: NICE Clinical Knowledge Summaries: Vitamin D deficiency in children[40]

A) 25(OH)D concentrations recorded in the 90 days prior to or on the index date (n=12,277)

B) 25(OH)D concentrations recorded between 91-180 days after index date (n=1,035)

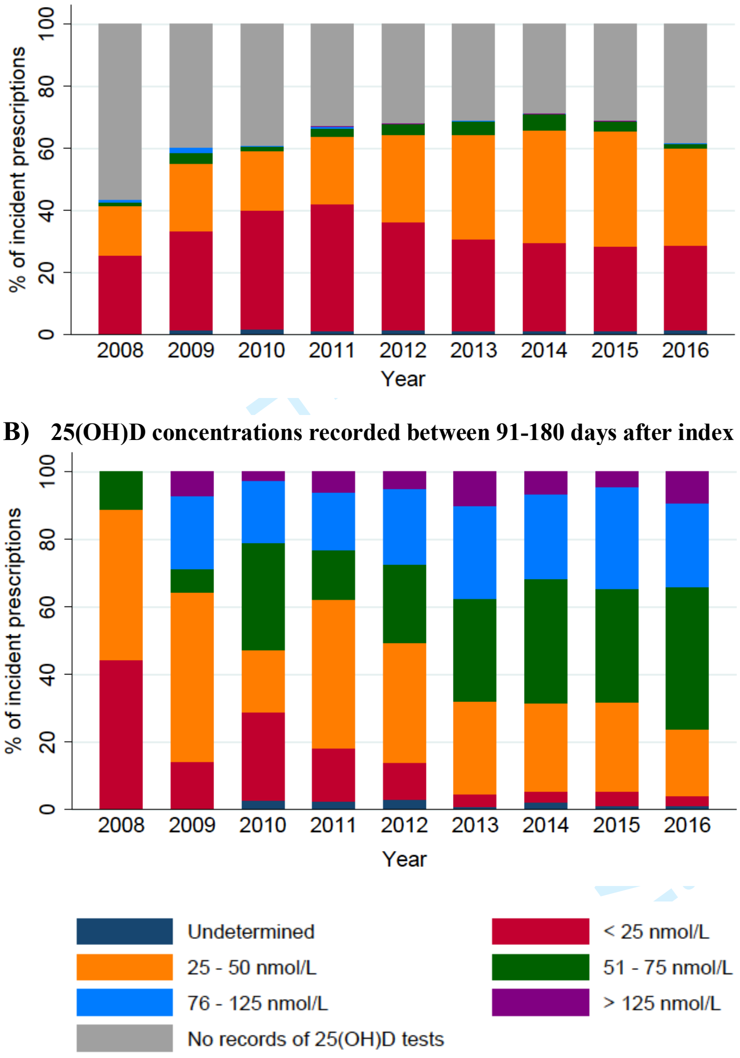

Figure 2: A) 25-hydroxyvitamin D concentrations recorded in the 90 days prior to or on the index date (n=12,277); **B)** 25-hydroxyvitamon D concentrations recorded between 91-180 days after index date (only children with known levels, n=1,035). Records with ambiguous unit of measurements were categorised as 'undetermined'.

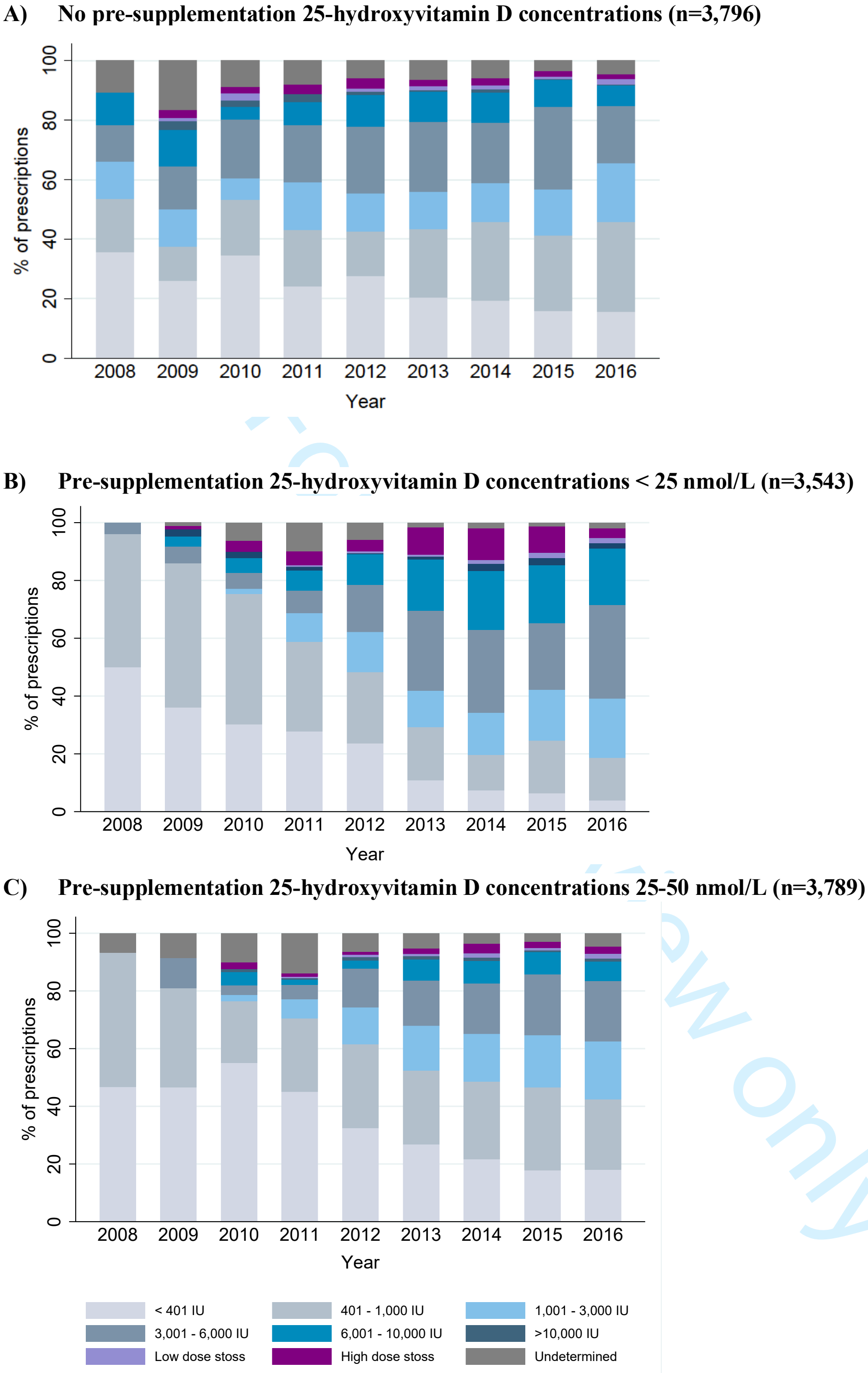

Figure 3: Dosages of incident vitamin D prescriptions presented in equivalent daily dose among children with **A)** no pre-supplementation 25(OH)D concentrations, **B)** 25(OH)D concentrations less than 25 nmol/L, and **C)** 25(OH)D concentrations between 25 - 50 nmol/L in the 90 days prior to their incident prescriptions.

For peer review only - http://bmjopen.bmj.com/site/about/guidelines.xhtml

Supplementary Table S1

Summary of UK guidance for the management of vitamin D deficiency in children

Supplementary Figure S1

Flowchart of study cohort selection

Supplementary Table S2

Drug code related to single-ingredient vitamin D products

Supplementary Table S3

Read codes related to vitamin D deficiency and 25-hydroxyvitamin D tests

Supplementary Table S4

No. of children with a record of diagnosis Read codes suggestive of vitamin D deficiency in the 90 days prior to or on the date of the incident prescription, stratified by pre-supplementation 25-hydroxyvitamin D concentrations

Supplementary Table S5

Multivariable Poisson regression models of incidence of vitamin D supplementation prescribing in children stratified by sex (Complete-cases only).

Supplementary Figure S2

Time trends in vitamin D supplementation prescribing in children by product types

Supplementary Table S6

Doses of vitamin D supplementation prescribed stratified by age group

Supplementary Table S7

Vitamin D supplementation dosing frequencies observed in the study cohort

For peer review only

Supplementary Table S1

Summary of UK guidance for the management of vitamin D deficiency in children

Guideline	Definition of vitamin D deficiency	Recommendations on supplementation for primary prevention	Recommendations on supplement for treatment of vitamin D deficiency
SACN (2007)	<25 nmol/L	0-6 months: 340 IU daily;<br>7 months to 3 years: 280 IU daily	No recommendations
NICE (2008)	<25 nmol/L	6 months to 4 years: 300 IU daily	No recommendations
DHSC (2012)	Not explicitly defined	6 months to 5 years: 280 - 340 IU daily<br>Those infants who are fed infant formula will not need vitamin drops until they are receiving less than 500ml of infant formula a day. Breastfed infants from one month of age if their mother has not taken vitamin D supplements throughout pregnancy.	No recommendations
BPABG (2012)	<25 nmol/L<br><br>25-50 nmol/L defined as insufficiency	6 months to 5 years: as per DHSC (2012) recommendations<br>Exclusively breastfed infants receive vitamin D supplements from soon after birth.	No recommendations
RCPCH (2012)	Not explicitly defined	As per NICE (2008) and DHSC (2012) recommendations	No recommendations
RCPCH (2013)	<25 nmol/L<br><br>25-50 nmol/L defined as insufficiency	Newborn up to 1 month: 300 - 400 IU daily<br>1 month - 18 years: 400 - 1,000 IU daily	Up to 6 months: 1,000 - 3,000 IU daily*<br>6 months - 12 years: 6,000 IU daily*<br>12 -18 years: 10,000 IU daily*<br>*For 4 - 8 weeks
NICE (2014)	<25 nmol/L	0 - 6 months: 340 IU daily<br>Older infants and children up to the age of 5: 280 IU daily	No recommendations
NOS (2015)	<25 nmol/L<br><br>25-50 nmol/L defined as insufficiency	As per DHSC (2012) recommendations	1- 6 months: 3,000 IU daily*<br>6 months - 12 years: 6,000 IU daily*<br>12 -18 years: 10,000 IU daily*<br>*For 8 – 12 weeks
SACN (2016)	<25 nmol/L	0 up to 1 year (including exclusively and partially breast-fed infants, from birth): 340 – 400 IU daily<br>1 year or older: 400 IU daily	No recommendations
NICE CKS (2016)	<25 nmol/L	As per SACN (2016) recommendations	1- 6 months: 3,000 IU daily*<br>6 months - 12 years: 6,000 IU daily*<br>12 -18 years: 10,000 IU daily*<br>*For 8 – 12 weeks

Key:

- SACN (2007): Scientific Advisory Committee on Nutrition - Update on Vitamin D[31]
- NICE (2008): The National Institute for Health and Care Excellence - Improving the nutrition of pregnant and breastfeeding mothers and children in low-income households[32]
- DHSC (2012): Department of Health and Social Care - Vitamin D - advice on supplements for at risk groups[33]
- BPABG (2012): British Paediatric and Adolescent Bone Group's position statement on vitamin D deficiency[34]
- RCPCH (2012): Royal College of Paediatrics and Child Health - Vitamin D: Position statement[35]
- RCPCH (2013): Royal College of Paediatrics and Child Health - Guide for Vitamin D in Childhood[36]
- NICE (2014): The National Institute for Health and Care Excellence - Vitamin D: increasing supplement use in at-risk groups[37]
- NOS (2015): National Osteoporosis Society - Vitamin D and Bone Health[38]
- SACN (2016): Scientific Advisory Committee on Nutrition - vitamin D and health report[39]
- NICE CKS (2016): The National Institute for Health and Care Excellence - Clinical Knowledge Summaries - Vitamin D deficiency in children[40]

For peer review only

Supplementary Figure S1

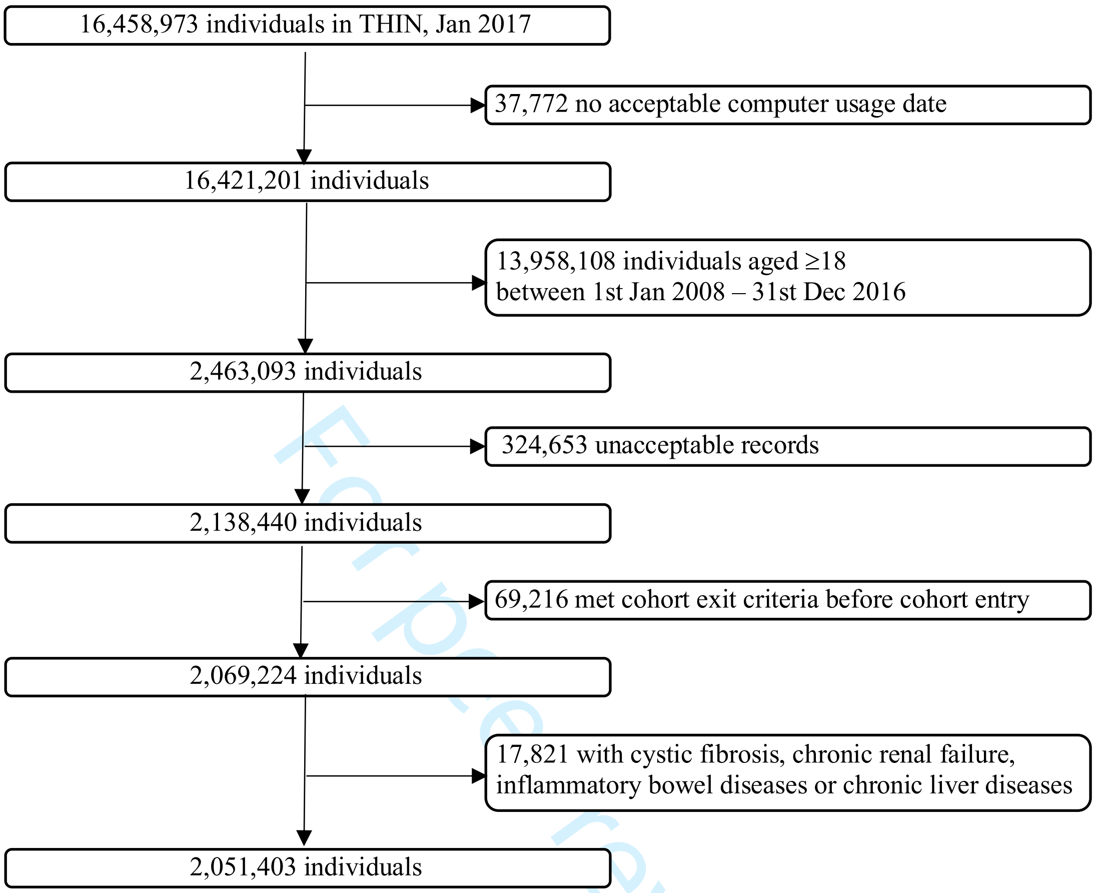

Figure S1: Flowchart of study cohort selection. The diagram displays the numbers of patients excluded from the THIN cohort and reasons for exclusion, leading to selection of the study cohort.

For peer review only

Supplementary Table S2

Drug code related to single-ingredient vitamin D products

2a. Drug code related to single-ingredient vitamin D products

Drug code	Description
29742978	colecalciferol 800unit capsules
30212978	colecalciferol 2,500units/drop oral drops sugar free
30582978	colecalciferol 40,000unit tablets
30643978	colecalciferol 800unit capsules
32236978	colecalciferol 4,000unit tablets
32237978	colecalciferol 4,000unit tablets
32265978	colecalciferol 4,000unit capsules
32267978	colecalciferol 1,000unit tablets
32317978	colecalciferol 50,000unit capsules
34135978	colecalciferol 3,000unit tablets
34138978	colecalciferol 800unit capsules
34144978	colecalciferol 1,000unit capsules
38846978	colecalciferol 20,000unit tablets
39354978	colecalciferol 50,000units/1ml oral solution unit dose ampoules sugar free
39355978	colecalciferol 50,000units/1ml oral solution unit dose ampoules sugar free
47464978	colecalciferol 2,740units/ml oral drops sugar free
47465978	colecalciferol 2,740units/ml oral drops sugar free
48011978	colecalciferol 15,000units/5ml oral solution
51017978	colecalciferol 15,000units/5ml oral solution
51018978	colecalciferol 15,000units/5ml oral solution
51473978	colecalciferol 200units/drop oral drops sugar free
51484978	colecalciferol 1,000unit capsules
52073979	colecalciferol 40,000unit capsules
52074979	colecalciferol 40,000unit capsules
52154979	colecalciferol 20,000unit capsules
53089979	colecalciferol 15,000units/5ml oral solution
53090979	colecalciferol 15,000units/5ml oral solution
53091979	colecalciferol 15,000units/5ml oral solution
53092979	colecalciferol 15,000units/5ml oral solution
53321979	ergocalciferol 125microgram tablets
53322979	ergocalciferol 125microgram tablets
53352979	colecalciferol 20,000unit tablets
53353979	colecalciferol 5,000unit tablets
53355979	colecalciferol 400unit tablets
53356979	colecalciferol 30,000unit capsules
53357979	colecalciferol 5,000unit capsules
53358979	colecalciferol 3,000unit capsules
53359979	ergocalciferol 12.5microgram tablets
53362979	colecalciferol 400unit capsules
53365979	colecalciferol 1,000unit capsules
53366979	colecalciferol 600unit capsules
53367979	colecalciferol 2,200unit capsules
53368979	colecalciferol 10,000unit capsules
53369979	colecalciferol 20,000unit capsules
53370979	colecalciferol 20,000unit capsules
53371979	colecalciferol 50,000unit capsules
53377978	colecalciferol 2,400units/ml oral drops sugar free
53378978	colecalciferol 2,400units/ml oral drops sugar free
53461978	ergocalciferol 12.5microgram tablets
53464978	colecalciferol 20,000unit capsules
53641979	ergocalciferol 250microgram tablets
53650979	ergocalciferol 1.25mg capsules
54637979	colecalciferol 500unit capsules
54638979	colecalciferol 500unit capsules
54716979	colecalciferol 1,000unit capsules
54790979	ergocalciferol 1.25mg capsules
54889979	colecalciferol 3,000units/ml oral solution
54890979	colecalciferol 2,000units/ml oral solution

For peer review only

2a. Drug code related to single-ingredient vitamin D products - continued

Drug code	Description
54891979	colecalciferol 2,000units/ml oral solution
54916979	colecalciferol 800unit tablets
54917979	colecalciferol 800unit tablets
55053979	colecalciferol 3,000unit tablets
55054979	colecalciferol 20,000unit tablets
55055979	colecalciferol 2,200unit tablets
55056979	colecalciferol 10,000unit tablets
55060979	colecalciferol 5,000unit tablets
55061979	ergocalciferol 3,000units/ml oral solution
55062979	ergocalciferol 3,000units/ml oral solution sugar free
55064979	colecalciferol 3,000units/ml oral solution sugar free
55065979	colecalciferol 20,000units/ml oral solution sugar free
55066979	colecalciferol 30,000unit capsules
55067979	colecalciferol 30,000unit capsules
55068979	colecalciferol 5,000unit capsules
55070979	colecalciferol 400unit capsules
55072979	colecalciferol 1,000unit capsules
55074979	colecalciferol 2,200unit capsules
55076979	colecalciferol 10,000unit capsules
55077979	colecalciferol 20,000unit capsules
55078979	colecalciferol 50,000unit capsules
55079979	colecalciferol 50,000unit capsules
55194978	colecalciferol 10,000units/ml oral solution sugar free
55195978	colecalciferol 10,000units/ml oral drops sugar free
55196978	colecalciferol 10,000units/ml oral drops sugar free
55197978	colecalciferol 10,000units/ml oral drops sugar free
55257978	colecalciferol 20,000unit capsules
55558979	ergocalciferol 50,000unit capsules
56123979	ergocalciferol 1.25mg capsules
56242979	colecalciferol 3,000unit tablets
56244979	colecalciferol 20,000unit tablets
56246979	colecalciferol 2,200unit tablets
56248979	colecalciferol 10,000unit tablets
56310979	colecalciferol 400unit tablets
56313979	colecalciferol 1,000unit tablets
56949978	colecalciferol 25,000unit tablets
56950978	colecalciferol 25,000unit tablets
57024978	colecalciferol 1,000unit tablets
57965979	colecalciferol 20,000unit capsules
58139979	colecalciferol 5,000unit tablets
58209979	ergocalciferol 3,000units/ml oral solution
58346979	colecalciferol 800unit capsules
58349979	colecalciferol 5,000unit capsules
58353979	colecalciferol 1,000unit capsules
58355979	colecalciferol 20,000unit capsules
58356979	colecalciferol 20,000unit capsules
58768979	colecalciferol 2,000units/ml oral solution sugar free
58769979	colecalciferol 2,000units/ml oral solution sugar free
58782979	colecalciferol 3,000units/ml oral solution sugar free
59522978	colecalciferol 2,000unit tablets
59525978	colecalciferol 40,000unit capsules
59527978	colecalciferol 5,000unit tablets
59528978	colecalciferol 20,000unit capsules
59769979	colecalciferol 800units/ml oral drops sugar free
59770979	colecalciferol 800units/ml oral drops sugar free
59771979	colecalciferol 400units/dose oral spray sugar free
59772979	colecalciferol 400units/dose oral spray sugar free
59843979	colecalciferol 20,000unit capsules
59981979	ergocalciferol 15,000units/5ml oral solution
60056979	ergocalciferol 300,000units/1ml solution for injection ampoules

For peer review only

2a. Drug code related to single-ingredient vitamin D products - continued

Drug code	Description
60224979	colecalciferol 300,000units/1ml solution for injection ampoules
60225979	ergocalciferol 300,000units/1ml solution for injection ampoules
60373979	fultium-d3 800unit capsules
60374979	colecalciferol 800unit capsules
60566979	vitamin d2 3,000units/ml oral solution sugar free
60568979	ergocalciferol 20,000units/ml oral solution sugar free
60572979	colecalciferol 20,000units/ml oral solution sugar free
60594979	colecalciferol 1,000unit tablets
61563979	colecalciferol 30,000units/5ml oral solution
61592979	colecalciferol 2,000units/ml oral drops sugar free
61593979	colecalciferol 2,000units/ml oral drops sugar free
61910979	colecalciferol 400unit tablets
61911979	colecalciferol 400unit tablets
62001979	colecalciferol 1,000units/ml oral solution
62002979	colecalciferol 1,000units/ml oral solution
62087979	colecalciferol 10,000units/ml oral solution
62088979	colecalciferol 10,000units/ml oral solution
62116979	colecalciferol 5,000unit capsules
62118979	colecalciferol 3,000unit capsules
62152979	colecalciferol 6,000units/5ml oral suspension
62324979	colecalciferol 20,000units/ml oral drops
62356979	ergocalciferol 12.5microgram tablets
62357979	ergocalciferol 12.5microgram tablets
62536979	colecalciferol 400unit capsules
62622979	colecalciferol 1,000unit tablets
62626979	colecalciferol 1,000unit capsules
62643979	colecalciferol 600unit capsules
62645979	colecalciferol 2,200unit capsules
62986979	colecalciferol 20,000unit capsules
63201979	colecalciferol 2,000unit capsules
64131979	colecalciferol 4,000units/5ml oral suspension
68364979	ergocalciferol 400units/5ml oral solution
68382979	ergocalciferol 20,000units/5ml oral suspension
68384979	ergocalciferol 20,000units/5ml oral solution
68392979	ergocalciferol 120units/5ml oral solution
68396979	ergocalciferol 100,000units/5ml oral solution
68780978	colecalciferol 20,000unit capsules
68781978	colecalciferol 20,000unit capsules
69915979	colecalciferol 400units/5ml oral suspension
69917979	colecalciferol 20,000units/5ml oral suspension
69920979	colecalciferol 1,000units/5ml oral suspension
70249978	ergocalciferol 1.25mg capsules
73011978	colecalciferol 25,000units/1ml oral solution unit dose ampoules sugar free
73012978	colecalciferol 25,000units/1ml oral solution unit dose ampoules sugar free
73102978	colecalciferol 3,200unit capsules
73128978	colecalciferol 10,000unit capsules
73129978	colecalciferol 20,000unit capsules
73301978	colecalciferol 20,000units/ml oral solution
73391978	colecalciferol 3,200unit capsules
73392978	colecalciferol 3,200unit capsules
77093978	colecalciferol 15,000units/5ml oral solution
78387978	colecalciferol 500unit orodispersible tablets sugar free
78388978	colecalciferol 500unit orodispersible tablets sugar free
78389978	colecalciferol 2,000unit orodispersible tablets sugar free
78390978	colecalciferol 2,000unit orodispersible tablets sugar free
78426978	colecalciferol 2,200unit tablets
78428978	colecalciferol 5,000unit tablets
78441978	colecalciferol 1,000unit capsules
78719978	colecalciferol 2,000unit tablets
78720978	colecalciferol 2,000unit tablets

For peer review only

2a. Drug code related to single-ingredient vitamin D products - continued

Drug code	Description
79321978	colecalciferol 200units/drop oral drops sugar free
79322978	colecalciferol 200units/drop oral drops sugar free
79338978	ergocalciferol 6,000units/5ml oral solution
79340978	ergocalciferol 10,000units/5ml oral solution
79935978	ergocalciferol 600,000units/1.5ml solution for injection ampoules
79960978	colecalciferol 280unit chewable tablets
79961978	colecalciferol 280unit chewable tablets
79993978	colecalciferol 20,000unit tablets
80016978	colecalciferol 400unit capsules
80018978	colecalciferol 1,000unit tablets
80019978	colecalciferol 1,000unit capsules
80022978	colecalciferol 20,000unit capsules
80147979	ergocalciferol 800units/5ml oral suspension
80151979	ergocalciferol 6,000units/5ml oral suspension
80153979	ergocalciferol 50,000units/5ml oral suspension
80157979	ergocalciferol 30,000units/5ml oral suspension
80159979	ergocalciferol 3,000units/5ml oral suspension
80169979	ergocalciferol 10,000units/5ml oral suspension
80416979	colecalciferol 15,000units/5ml oral suspension
80883998	colecalciferol 3,000units/ml oral solution sugar free
80885998	colecalciferol 3,000units/ml oral solution sugar free
80886998	colecalciferol 20,000units/ml oral solution sugar free
80887998	colecalciferol 20,000units/ml oral solution sugar free
80914998	colecalciferol 2,000units/ml oral drops sugar free
80915998	colecalciferol 2,000units/ml oral drops sugar free
80947998	ergocalciferol 20,000units/ml oral solution sugar free
80952998	colecalciferol 800unit capsules
80958998	colecalciferol 20,000unit capsules
81004998	colecalciferol 30,000unit capsules
81007998	colecalciferol 30,000unit capsules
81009998	colecalciferol 10,000unit capsules
81010998	colecalciferol 10,000unit capsules
81011998	colecalciferol 2,500unit capsules
81012998	colecalciferol 2,500unit capsules
81013998	colecalciferol 1,000unit capsules
81014998	colecalciferol 1,000unit capsules
81015998	colecalciferol 400unit capsules
81074998	colecalciferol 5,000unit capsules
81138998	colecalciferol 500 units tablets
81252998	colecalciferol 1,000unit tablets
81253998	colecalciferol 1,000unit tablets
81328998	colecalciferol 800unit capsules
81329998	colecalciferol 800unit capsules
81335998	colecalciferol 20,000unit capsules
81464998	colecalciferol 400unit capsules
81568998	colecalciferol 10000units/ml oral solution
81612998	colecalciferol 1,000unit tablets
81624998	colecalciferol 10,000units/5ml oral suspension
81631998	colecalciferol 2,200unit capsules
81710998	colecalciferol 50,000unit capsules
82283998	colecalciferol 15,000units/5ml oral solution
82513998	colecalciferol 20,000unit capsules
82514998	ergocalciferol 1.25mg capsules
82946978	colecalciferol 20,000unit tablets
82956978	colecalciferol 20,000unit capsules
83392978	colecalciferol 50,000unit tablets
83393978	colecalciferol 50,000unit tablets
83468978	colecalciferol 20,000unit tablets
83469978	colecalciferol 10,000unit tablets
83480978	colecalciferol 800unit capsules

For peer review only

2a. Drug code related to single-ingredient vitamin D products - continued

Drug code	Description
83743978	ergocalciferol 1,000units/5ml oral solution
83744978	colecalciferol 5,000units/5ml oral solution
83746978	colecalciferol 5,000units/5ml oral solution
83747978	colecalciferol 10,000units/5ml oral solution
83748978	colecalciferol 10,000units/5ml oral solution
83750978	colecalciferol 10,000units/5ml oral solution
84072978	colecalciferol 5,000unit tablets
84414978	ergocalciferol 1,500units/ml oral solution sugar free
84586978	colecalciferol 20,000unit tablets
84592978	colecalciferol 400unit tablets
84600978	colecalciferol 1,000unit tablets
84680998	colecalciferol oral liquid
85005998	ergocalciferol oral liquid
85220998	ergocalciferol 600,000units/2ml solution for injection ampoules
85224998	ergocalciferol 300,000units/1ml solution for injection ampoules
85329978	colecalciferol 1,000unit tablets
86614979	ergocalciferol 300,000units/1ml solution for injection ampoules
86615979	ergocalciferol 300,000units/1ml solution for injection ampoules
93163990	ergocalciferol 600,000units/2ml solution for injection ampoules
93164990	ergocalciferol 300,000units/1ml solution for injection ampoules
93384992	calciferol 10 mcg tab
93390992	calciferol 1,000 iu i/u tab
93401992	calciferol 5000 i/u tab
94809990	ergocalciferol 1.25mg tablets
94810990	ergocalciferol 250microgram tablets
95606992	ergocalciferol 600ku/1.5ml solution
95872992	calciferol 3,000 iu tab
95873992	calciferol 30,000 iu/ml inj
95875992	calciferol soln 400u/5ml in arachis oil sol
95877992	calciferol 40,000 iu/ml inj
95878992	calciferol 400 iu/ml sol
96131992	calciferol 400 iu/ml i/u syr
96132992	calciferol .25 mg cap
96133992	calciferol 1 mg cap
96134992	calciferol 1,000 iu i/u cap
96599992	calciferol 75 mcg inj
96600992	ergocalciferol 250microgram tablets
96601992	calciferol 20,000 iu cap
96788998	ergocalciferol 400000units/ml oral solution
97023992	calciferol 100,000 iu/ml inj
97024992	calciferol 600,000 iu/ml inj
97032992	calciferol 300 iu/ml i/u syr
97033992	calciferol drops liq
97034992	calciferol 400 i/u inj
97035992	calciferol 9,000 iu/ml liq
97036992	calciferol 1,000 iu/5ml sol
97037992	calciferol 300 mg tab
97038992	calciferol 15 mcg tab
97238989	ergocalciferol 1.25mg tablets
97238990	ergocalciferol 250microgram tablets
97767998	calciferol bp 3000units/ml solution
98374992	vitamin d2 tab
98421997	ergocalciferol 1.25mg tablets
98421998	ergocalciferol 250microgram tablets
98422997	ergocalciferol 1,000units/5ml oral suspension
98422998	ergocalciferol 3,000units/ml oral solution sugar free
98925998	ergocalciferol 600,000units/2ml solution for injection ampoules
99762990	ergocalciferol 250microgram tablets
99763988	ergocalciferol 250microgram tablets
99763990	ergocalciferol 300,000units/1ml solution for injection ampoules

For peer review only

2a. Drug code related to combination vitamin D products

Drug code	Description
34026978	colecalciferol 1,000unit / calcium carbonate 2.5g chewable tablets
34027978	colecalciferol 1,000unit / calcium carbonate 2.5g chewable tablets
52015979	colecalciferol 800unit / calcium carbonate 1.25g chewable tablets
52016979	colecalciferol 800unit / calcium carbonate 1.25g tablets
56947978	colecalciferol 800unit / calcium carbonate 2.5g chewable tablets
56948978	colecalciferol 800unit / calcium carbonate 2.5g chewable tablets
58270979	generic caldrink d3 liquid
60543979	colecalciferol 100unit / calcium carbonate 400mg chewable tablets
60544979	colecalciferol 100unit / calcium carbonate 400mg chewable tablets
61594979	colecalciferol 150unit / calcium 250mg tablets
61595979	colecalciferol 150unit / calcium 250mg tablets
64223979	calcium carbonate 400mg / ergocalciferol 2.5microgram tablets
64224979	calcium carbonate 400mg / ergocalciferol 2.5microgram tablets
64830979	calcium and ergocalciferol tablets
64831979	colecalciferol 100unit / calcium carbonate 400mg tablets
68756978	colecalciferol 880unit / calcium carbonate 2.5g chewable tablets
68757978	colecalciferol 880unit / calcium carbonate 2.5g chewable tablets
73294978	generic healthy start children's vitamin drops
73598978	colecalciferol 100unit / calcium carbonate 400mg tablets
80849998	calcium carbonate 400mg with colecalciferol 100 units chewable tablets
81191998	colecalciferol 400unit / calcium carbonate 1.5g tablets
81268998	colecalciferol 200unit / calcium carbonate 750mg tablets
81269998	colecalciferol 200unit / calcium carbonate 750mg tablets
81655998	colecalciferol 800unit / calcium carbonate 1.25g chewable tablets
81656998	colecalciferol 800unit / calcium carbonate 1.25g chewable tablets
82214998	colecalciferol 400unit / calcium carbonate 1.25g tablets
82215998	colecalciferol 400unit / calcium carbonate 1.25g chewable tablets
82229978	generic littlevit multivitamin drops
82344998	colecalciferol 400unit / calcium carbonate 1.5g tablets
82429979	colecalciferol 400unit / calcium carbonate 1.25g chewable tablets
82483998	colecalciferol 400unit / calcium carbonate 1.5g chewable tablets
82546978	colecalciferol 400unit / calcium carbonate 1.5g chewable tablets
82752998	generic sandocal+d 1200 effervescent tablets
82753998	calcium lactate gluconate with calcium carbonate and colecalciferol 1200mg calcium with 800iu effervescent tablets
82966998	generic sandocal+d 600 effervescent tablets
82967998	calcium lactate gluconate with calcium carbonate and colecalciferol 600mg calcium with 400iu effervescent tablets
83420998	colecalciferol 400unit / calcium carbonate 1.25g chewable tablets
83638978	colecalciferol 1,000unit / folic acid 400microgram capsules
83639978	colecalciferol 1,000unit / folic acid 400microgram capsules
84212998	risedronate sodium 35mg with calcium carbonate 2500mg & colecalciferol 22micrograms tablets and granules
84286998	colecalciferol 400unit / calcium carbonate 1.5g effervescent tablets
84287998	colecalciferol 400unit / calcium carbonate 1.5g chewable tablets
84292998	colecalciferol 400unit / calcium carbonate 1.5g chewable tablets
84362998	vitamins c & d3 with folic acid tablets
84363998	vitamins c & d3 with folic acid tablets
86598998	alendronic acid 70mg / colecalciferol 70microgram tablets
86599998	alendronic acid 70mg / colecalciferol 70microgram tablets
88002998	dolomite with vitamin d tablets
88144997	calcium carbonate 600mg with vitamin d % micrograms with minerals chewable tablets
88144998	calcium carbonate 600mg with vitamin d and minerals 5 micrograms tablets
88225998	calcium carbonate 600mg with vitamin d and 5 micrograms tablets
88233979	generic abidec multivitamin drops
89183998	colecalciferol 400unit / calcium carbonate 1.25g chewable tablets
89439998	colecalciferol 440unit / calcium carbonate 1.25g effervescent granules sachets
89637998	colecalciferol 400unit / calcium carbonate 1.5g chewable tablets
89647998	colecalciferol 400unit / calcium carbonate 1.5g chewable tablets
89828997	multivitamin and mineral chewable tablets

For peer review only

2a. Drug code related to combination vitamin D products - continued

Drug code	Description
89828998	calcium carbonate 600mg with vitamin d and minerals 5 micrograms tablets
90498998	nicotinic acid with colecalciferol and calcium gluconate tablet
91053998	colecalciferol 800unit / calcium phosphate 3.1g oral powder sachets
91526998	calcium carbonate 600mg with vitamin d and 5 micrograms tablets
91987979	colecalciferol 400unit / calcium carbonate 1.5g chewable tablets
91989979	colecalciferol 400unit / calcium carbonate 1.25g chewable tablets
91990979	colecalciferol 400unit / calcium carbonate 1.25g chewable tablets
91991979	colecalciferol 400unit / calcium carbonate 1.25g chewable tablets
91993979	colecalciferol 400unit / calcium carbonate 1.25g chewable tablets
91994979	colecalciferol 400unit / calcium carbonate 1.25g chewable tablets
91995979	colecalciferol 200unit / calcium carbonate 1.25g chewable tablets
91996979	colecalciferol 400unit / calcium carbonate 1.25g chewable tablets
91997979	colecalciferol 400unit / calcium carbonate 1.25g chewable tablets
91998979	colecalciferol 200unit / calcium carbonate 1.25g chewable tablets
92000979	colecalciferol 200unit / calcium carbonate 1.25g chewable tablets
92001979	colecalciferol 200unit / calcium carbonate 1.25g chewable tablets
92004979	calcium and ergocalciferol tablets
93127992	calcium 500mgs & vitamin d 400iu tab
93276998	generic abidec multivitamin drops
94021990	calcium and ergocalciferol tablets
94089992	calcium + vitamin d 300 mg tab
94756992	calcium & vitamin d 6000 i/u tab
96015992	vit.a /vit.b1 /vit.b2 /vit.c /vit.d2 /ni pow
96020992	calcium and ergocalciferol tablets
96737998	calciferol with calcium tablets
96789998	calcium and ergocalciferol tablets
96897998	calcium and ergocalciferol tablets
96920998	colecalciferol 800unit / calcium phosphate 3.1g oral powder sachets
96959996	colecalciferol 440unit / calcium carbonate 1.25g effervescent granules sachets
96959997	colecalciferol 400unit / calcium carbonate 1.25g chewable tablets
96959998	colecalciferol 200unit / calcium carbonate 1.25g chewable tablets
97031992	calciferol/calcium gluconate tab
97064992	calcium 500mgs & vitamin d 200iu i/u tab
97138998	calcium and ergocalciferol tablets
97248990	calcium and ergocalciferol tablets
97780990	calcium and ergocalciferol tablets
97815990	calcium and ergocalciferol tablets
97935998	generic abidec multivitamin drops
98247992	vit.a /vit.b1 /vit.b2 /vit.c /vit.d2 /ni eli
98249990	calcium and ergocalciferol tablets
98581990	calcium and ergocalciferol tablets
98700997	colecalciferol 200unit / calcium carbonate 1.25g chewable tablets
98700998	colecalciferol 200unit / calcium carbonate 1.25g chewable tablets
99018990	calcium and ergocalciferol tablets
99733992	ascorbic acid/vitamin a/vitamin d 2 25 mg tab

For peer review only

Supplementary Table S3

Read codes related to vitamin D deficiency and 25-hydroxyvitamin D tests

Read code	Description
C28..00	vitamin d deficiency
C28..11	osteomalacia
C28..12	rickets
C280.00	active rickets
C282.00	osteomalacia unspecified
C28z.00	avitaminosis d nos
C2B..00	vitamin d insufficiency
J690.11	coeliac rickets
K080300	renal rickets
44LA.00	serum vitamin d
44LM.00	plasma 25-hydroxyvitamin d3 level
44LP.00	serum 25-hydroxy vitamin d3 level
44Lg.00	serum vitamin d2 level
44Ln.00	serum 25-hydroxyvitamin d2 level
4QB4.00	vitamin d level
4QB4000	vitamin d2 level
4QB4100	vitamin d3 level
4QB4200	25-hydroxyvitamin d2 level
4QB4300	25-hydroxyvitamin d3 level
4QB4400	combined total vitamin d2 and d3 level
4QB4500	25-hydroxyvitamin d level
4QB4600	total 25-hydroxyvitamin d level
4QB4700	serum total 25-hydroxy vitamin d level
5788	radiobioassay - vitamin d

For peer review only

Supplementary Table S4

No. of children with a record of diagnosis Read codes suggestive of vitamin D deficiency in the 90 days prior to or on the date of the incident prescription, stratified by pre-supplementation 25-hydroxyvitamin D concentrations

	25-hydroxyvitamin D concentrations at initiation of supplementation (nmol/L)							
	**<10** (n=3,738)	**10 - 20** (n=3,898)	**>20 - 30** (n=441)	**>30 - 50** (n=31)	**>50** (n=9)	**No test** (n=3,986)	**Undetermined** (n=174)	**Total** (n=12,277)
**No. of children with symptoms, n**								
Musculoskeletal and non-specific pain	750	769	67	4	0	340	23	1,953
Tiredness and fatigue	556	547	46	3	0	106	5	1,263
Failure to thrive	25	33	7	1	0	36	3	105
Bone fracture	13	15	2	1	0	42	6	79
Skeletal deformity	15	26	8	1	0	35	2	87
Numbness or paraesthesia	14	9	1	0	0	7	0	31
Hypocalcaemia	9	2	2	0	0	16	0	29
Seizure or tetany	1	3	0	0	0	16	1	21
Abnormal gait	4	9	1	0	0	6	1	21
Muscle weakness	9	7	2	0	0	3	0	21
Delay in motor development	0	0	0	0	0	5	0	5
Cardiomyopathy	0	0	0	0	0	2	0	2
**Total, n (%)**	1,396 (37.3)	1,420 (36.4)	136 (30.8)	10 (32.3)	0 (0)	614 (15.4)	41 (23.6)	3,617 (29.5)

For peer review only

Supplementary Table S5

Multivariable Poisson regression models of incidence of vitamin D supplementation prescribing in children stratified by sex (complete-case analysis)

	Male	Female
	IRR[a] (95% CI)	IRR[a] (95% CI)
**Age group**		
Up to 6 months	1.00 (Reference)	1.00 (Reference)
6 months - 4 years	2.33 (1.11 – 4.92)	1.37 (0.73 – 2.56)
5 - 11 years	3.08 (1.47 – 6.49)	2.42 (1.30 – 4.51)
12 - 17 years	5.35 (2.54 – 11.24)	8.15 (4.38 – 15.17)
**Ethnicity**		
White	1.00 (Reference)	1.00 (Reference)
Asian	6.59 (5.92 – 7.33)	6.24 (5.71 – 6.82)
Black	3.67 (3.21 – 4.19)	3.63 (3.26 – 4.05)
Others	3.47 (2.79 – 4.32)	3.79 (3.18 – 4.51)
Mixed	2.32 (1.88 – 2.86)	2.31 (1.94 – 2.75)
**Townsend index**		
1 (least deprived)	1.00 (Reference)	1.00 (Reference)
2	1.03 (0.88 – 1.21)	0.97 (0.85 – 1.11)
3	1.14 (0.98 – 1.33)	1.12 (0.98 – 1.27)
4	1.32 (1.14 – 1.53)	1.31 (1.16 – 1.49)
5 (most deprived)	1.33 (1.13 – 1.56)	1.41 (1.24 – 1.61)
**Country**		
England	1.00 (Reference)	1.00 (Reference)
Wales	0.28 (0.18 – 0.42)	0.31 (0.21 – 0.45)
Scotland	0.31 (0.23 – 0.40)	0.23 (0.17 – 0.30)
Northern Ireland	0.37 (0.23 – 0.61)	0.22 (0.13 – 0.38)
**Year**		
2008	1.00 (Reference)	1.00 (Reference)
2009	2.06 (1.30 – 3.26)	3.03 (1.98 – 4.66)
2010	2.88 (1.87 – 4.45)	4.68 (3.11 – 7.04)
2011	4.70 (3.11 – 7.11)	7.87 (5.30 – 11.68)
2012	9.62 (6.46 – 14.34)	15.19 (10.32 – 22.37)
2013	14.37 (9.68 – 21.32)	20.84 (14.18 – 30.63)
2014	17.62 (11.87 – 26.16)	26.00 (17.69 – 38.22)
2015	18.02 (12.10 – 26.85)	29.35 (19.92 – 43.25)
2016	20.07 (13.43 – 29.98)	31.74 (21.49 – 46.88)

Abbreviations: CI, confidence interval; IRR = incidence rate ratio.

[a]Adjusted for all variables listed in the table. The multilevel models included the general practice as a random effect.

For peer review only

Supplementary Figure S2

Time trends in vitamin D supplementation prescribing in children by product types (n=11,743)

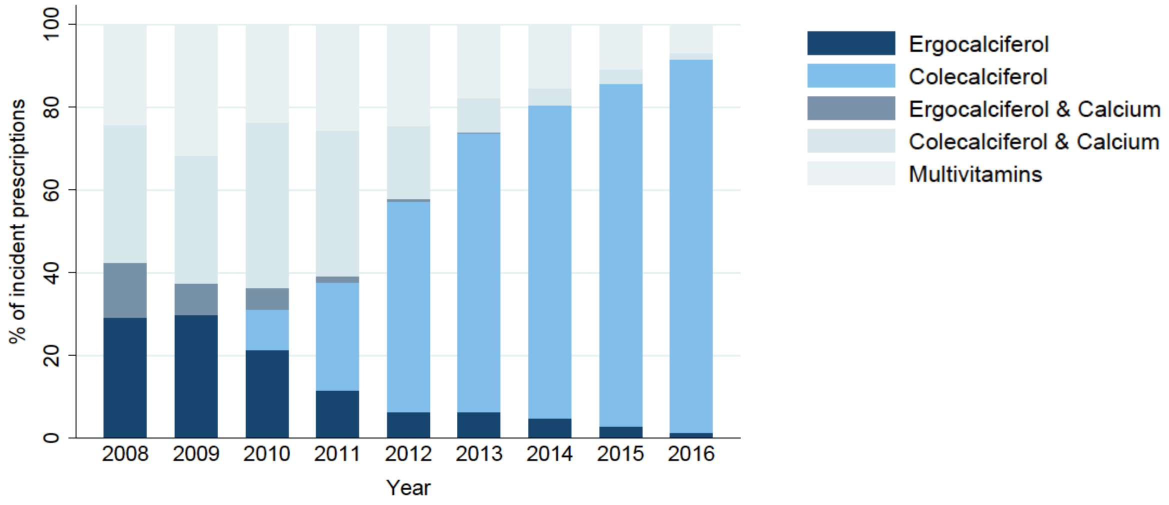

peer review only

Supplementary Table S6

Doses of vitamin D supplementation prescribed stratified by age group

	**Age group**			
	**Up to 6 months (n = 29)**	**6 months – 4 years (n = 1,670)**	**5 - 11 years (n = 3,504)**	**12 - 17 years (n = 6,540)**
Equivalent daily dose, n (%)	25 (86.2%)	1,460 (87.4%)	3,193 (91.1%)	5,929 (90.7%)
≤400 IU/day	8	707	1,110	640
401 – 1,000 IU/day	1	178	625	2,012
1,001 – 3,000 IU/day	15	170	437	1,073
3,001 – 6,000 IU/day	1	377	869	1,082
6,001 – 10,000 IU/day	0	25	129	1,016
>10,000 IU/day	0	3	23	106
Stoss therapy, n (%)	0	24 (1.7%)	94 (2.7%)	437 (6.7%)
<150,000 IU	0	10	32	86
150,000 – 300,000 IU	0	14	61	290
>300,000 IU	0	0	1	61
Undetermined, n (%)	4 (13.8%)	186 (11.1%)	217 (6.2%)	174 (2.7%)

For peer review only

Supplementary Table S7

Vitamin D supplementation dosing frequencies observed in the study cohort (n=11,743)

**Dosing Frequencies**	**n (%)**
Once daily	7,376 (62.8)
Once every 7 days	1,337 (11.4)
Twice a day	1,012 (8.6)
Undetermined	581(5)
Stoss	555 (4.7)
Once 2 times a week	267 (2.3)
Once every 14 days	227 (1.9)
Once 3 times a week	157 (1.3)
Once every 2 days	93 (0.8)
Once every 28 days	41 (0.4)
3 times a day	35 (0.3)
Once every 42 days	19 (0.2)
Others	43 (0.4)

For peer review only

For peer review only - http://bmjopen.bmj.com/site/about/guidelines.xhtml

The RECORD statement – checklist of items, extended from the STROBE statement, that should be reported in observational studies using routinely collected health data.

	Item No.	STROBE items	Location in manuscript where items are reported	RECORD items	Location in manuscript where items are reported
**Title and abstract**					
	1	(a) Indicate the study's design with a commonly used term in the title or the abstract (b) Provide in the abstract an informative and balanced summary of what was done and what was found		RECORD 1.1: The type of data used should be specified in the title or abstract. When possible, the name of the databases used should be included.<br><br>RECORD 1.2: If applicable, the geographic region and timeframe within which the study took place should be reported in the title or abstract.<br><br>RECORD 1.3: If linkage between databases was conducted for the study, this should be clearly stated in the title or abstract.	1,2<br><br>1,2<br><br>N/A
**Introduction**					
Background rationale	2	Explain the scientific background and rationale for the investigation being reported			4,5
Objectives	3	State specific objectives, including any prespecified hypotheses			4,5
**Methods**					
Study Design	4	Present key elements of study design early in the paper			5,6
Setting	5	Describe the setting, locations, and relevant dates, including periods of recruitment, exposure, follow-up, and data collection			5,6

For peer review only

Participants	6	*(a) Cohort study* - Give the eligibility criteria, and the sources and methods of selection of participants. Describe methods of follow-up<br>*Case-control study* - Give the eligibility criteria, and the sources and methods of case ascertainment and control selection. Give the rationale for the choice of cases and controls<br>*Cross-sectional study* - Give the eligibility criteria, and the sources and methods of selection of participants<br><br>*(b) Cohort study* - For matched studies, give matching criteria and number of exposed and unexposed<br>*Case-control study* - For matched studies, give matching criteria and the number of controls per case		RECORD 6.1: The methods of study population selection (such as codes or algorithms used to identify subjects) should be listed in detail. If this is not possible, an explanation should be provided.<br><br>RECORD 6.2: Any validation studies of the codes or algorithms used to select the population should be referenced. If validation was conducted for this study and not published elsewhere, detailed methods and results should be provided.<br><br>RECORD 6.3: If the study involved linkage of databases, consider use of a flow diagram or other graphical display to demonstrate the data linkage process, including the number of individuals with linked data at each stage.	6, 7, Data sharing, Supplementary<br><br>N/A<br><br>N/A
Variables	7	Clearly define all outcomes, exposures, predictors, potential confounders, and effect modifiers. Give diagnostic criteria, if applicable.		RECORD 7.1: A complete list of codes and algorithms used to classify exposures, outcomes, confounders, and effect modifiers should be provided. If these cannot be reported, an explanation should be provided.	6,7,8 Supplementary
Data sources/ measurement	8	For each variable of interest, give sources of data and details of methods of assessment (measurement).<br>Describe comparability of assessment methods if there is more than one group			6,7,8

For peer review only

Bias	9	Describe any efforts to address potential sources of bias			6,7,8
Study size	10	Explain how the study size was arrived at			5,6, Supplementary
Quantitative variables	11	Explain how quantitative variables were handled in the analyses. If applicable, describe which groupings were chosen, and why			6,7,8,9
Statistical methods	12	(a) Describe all statistical methods, including those used to control for confounding<br>(b) Describe any methods used to examine subgroups and interactions<br>(c) Explain how missing data were addressed<br>(d) *Cohort study* - If applicable, explain how loss to follow-up was addressed<br>*Case-control study* - If applicable, explain how matching of cases and controls was addressed<br>*Cross-sectional study* - If applicable, describe analytical methods taking account of sampling strategy<br>(e) Describe any sensitivity analyses			9,10
Data access and cleaning methods		..		RECORD 12.1: Authors should describe the extent to which the investigators had access to the database population used to create the study population.	18

For peer review only

				RECORD 12.2: Authors should provide information on the data cleaning methods used in the study.	
Linkage		..		RECORD 12.3: State whether the study included person-level, institutional-level, or other data linkage across two or more databases. The methods of linkage and methods of linkage quality evaluation should be provided.	N/A
**Results**					
Participants	13	(a) Report the numbers of individuals at each stage of the study (*e.g.*, numbers potentially eligible, examined for eligibility, confirmed eligible, included in the study, completing follow-up, and analysed)<br>(b) Give reasons for non-participation at each stage.<br>(c) Consider use of a flow diagram		RECORD 13.1: Describe in detail the selection of the persons included in the study (*i.e.,* study population selection) including filtering based on data quality, data availability and linkage. The selection of included persons can be described in the text and/or by means of the study flow diagram.	5,6
Descriptive data	14	(a) Give characteristics of study participants (*e.g.*, demographic, clinical, social) and information on exposures and potential confounders<br>(b) Indicate the number of participants with missing data for each variable of interest<br>(c) *Cohort study* - summarise follow-up time (*e.g.*, average and total amount)			10<br>Table 1
Outcome data	15	*Cohort study* - Report numbers of outcome events or summary measures over time<br>*Case-control study* - Report numbers in each exposure			10,11,12,13<br>Table 2<br>Figure 1<br>Figure 2<br>Figure 3

For peer review only

		category, or summary measures of exposure<br>*Cross-sectional study* - Report numbers of outcome events or summary measures			
Main results	16	(a) Give unadjusted estimates and, if applicable, confounder-adjusted estimates and their precision (e.g., 95% confidence interval). Make clear which confounders were adjusted for and why they were included<br>(b) Report category boundaries when continuous variables were categorized<br>(c) If relevant, consider translating estimates of relative risk into absolute risk for a meaningful time period			10,11,12,13
Other analyses	17	Report other analyses done—e.g., analyses of subgroups and interactions, and sensitivity analyses			10,11,12,13
**Discussion**					
Key results	18	Summarise key results with reference to study objectives			13
Limitations	19	Discuss limitations of the study, taking into account sources of potential bias or imprecision. Discuss both direction and magnitude of any potential bias		RECORD 19.1: Discuss the implications of using data that were not created or collected to answer the specific research question(s). Include discussion of misclassification bias, unmeasured confounding, missing data, and changing eligibility over time, as they pertain to the study being reported.	16
Interpretation	20	Give a cautious overall interpretation of results considering objectives,			13, 14, 15

For peer review only

		limitations, multiplicity of analyses, results from similar studies, and other relevant evidence			
Generalisability	21	Discuss the generalisability (external validity) of the study results			16
**Other Information**					
Funding	22	Give the source of funding and the role of the funders for the present study and, if applicable, for the original study on which the present article is based			18
Accessibility of protocol, raw data, and programming code		..		RECORD 22.1: Authors should provide information on how to access any supplemental information such as the study protocol, raw data, or programming code.	18

*Reference: Benchimol EI, Smeeth L, Guttmann A, Harron K, Moher D, Petersen I, Sørensen HT, von Elm E, Langan SM, the RECORD Working Committee. The REporting of studies Conducted using Observational Routinely-collected health Data (RECORD) Statement. *PLoS Medicine* 2015; in press.

*Checklist is protected under Creative Commons Attribution (CC BY) license.

For peer review only
